# Genome-Wide Characterization of Host Transcriptional and Epigenetic Alterations During HIV Infection of T Lymphocytes

**DOI:** 10.3389/fimmu.2020.02131

**Published:** 2020-09-10

**Authors:** Xi Zeng, Joseph Chi-Ching Tsui, Mai Shi, Jie Peng, Cyanne Ye Cao, Lea Ling-Yu Kan, Carol Po-Ying Lau, Yonghao Liang, Lingyi Wang, Li Liu, Zhiwei Chen, Stephen Kwok-Wing Tsui

**Affiliations:** ^1^Agricultural Bioinformatics Key Laboratory of Hubei Province, Hubei Engineering Technology Research Center of Agricultural Big Data, College of Informatics, Huazhong Agricultural University, Wuhan, China; ^2^School of Biomedical Sciences, The Chinese University of Hong Kong, Hong Kong, China; ^3^Department of Orthopaedics and Traumatology, The Chinese University of Hong Kong, Hong Kong, China; ^4^Acquired Immunodeficiency Syndrome (AIDS) Institute, The University of Hong Kong, Hong Kong, China; ^5^Hong Kong Bioinformatics Centre, The Chinese University of Hong Kong, Hong Kong, China; ^6^Centre for Microbial Genomics and Proteomics, The Chinese University of Hong Kong, Hong Kong, China

**Keywords:** HIV infection, gene expression, DNA methylation, genome, apoptosis

## Abstract

**Background and methods:** Host genomic alterations are closely related to dysfunction of CD4^+^ T lymphocytes in the HIV–host interplay. However, the roles of aberrant DNA methylation and gene expression in the response to HIV infection are not fully understood. We investigated the genome-wide DNA methylation and transcriptomic profiles in two HIV-infected T lymphocyte cell lines using high-throughput sequencing.

**Results:** Based on DNA methylation data, we identified 3,060 hypomethylated differentially methylated regions (DMRs) and 2,659 hypermethylated DMRs in HIV-infected cells. Transcription-factor-binding motifs were significantly associated with methylation alterations, suggesting that DNA methylation modulates gene expression by affecting the binding to transcription factors during HIV infection. In support of this hypothesis, genes with promoters overlapping with DMRs were enriched in the biological function related to transcription factor activities. Furthermore, the analysis of gene expression data identified 1,633 upregulated genes and 2,142 downregulated genes on average in HIV-infected cells. These differentially expressed genes (DEGs) were significantly enriched in apoptosis-related pathways. Our results suggest alternative splicing as an additional mechanism that may contribute to T-cell apoptosis during HIV infection. We also demonstrated a genome-scale correlation between DNA methylation and gene expression in HIV-infected cells. We identified 831 genes with alterations in both DNA methylation and gene expression, which were enriched in apoptosis. Our results were validated using various experimental methods. In addition, consistent with our *in silico* results, a luciferase assay showed that the activity of the *PDX1* and *SMAD3* promoters was significantly decreased in the presence of HIV proteins, indicating the potential of these genes as genetic markers of HIV infection.

**Conclusions:** Our results suggest important roles for DNA methylation and gene expression regulation in T-cell apoptosis during HIV infection. We propose a list of novel genes related to these processes for further investigation. This study also provides a comprehensive characterization of changes occurring at the transcriptional and epigenetic levels in T cells in response to HIV infection.

## Introduction

The human immunodeficiency virus (HIV) is a lentivirus belonging to the retrovirus family that infects human immune cells. HIV infection is the cause of the acquired immune deficiency syndrome (AIDS), which remains one of the major threats to human health worldwide. Around 37.9 million people were living with HIV/AIDS worldwide and 770,000 were killed by HIV-related illness in 2018 (1).

DNA methylation in the human genome can influence the transcription and replication of HIV-1 (2). HIV can also trigger changes in DNA methylation in the host genome, which are identified frequently in immune-related genes, thus explaining how the virus evades the host immune system. For example, Abdel-Hameed et al. demonstrated that DNA methylation of the *FOXP3* gene was negatively correlated with the level of its protein product. Infection of T cells with HIV-1 led to aberrant DNA methylation in the promoter of *FOXP3*, which is a biomarker of regulatory T cell (Tregs) (3). Moreover, DNA methylation of *IFN-*γ (4), *GNE* (5), *IGFBP6*, and *SATB2* (6) also reportedly regulated gene expression in HIV-1-infected T cell.

CD4^+^ T cells are the main and early target during HIV infection (3). Depletion of CD4^+^ T cells is one of the key features of HIV infection, which is mainly attributed to apoptosis (7). Many studies have reported the critical roles of DNA methylation in apoptosis (8–12). For example, Jin et al. reported that *HIPK2* induced cell apoptosis by activating *SMAD3* and the Wnt–Notch pathway in the kidneys of HIV-transgenic mice (8), whereas galectin-3 induced cell death in HIV-1-infected macrophages in a caspase-independent manner related to endonuclease G location in cells (10). In turn, *SATB2*, which is involved in the apoptosis of osteoblast-like MG63 cells, but not of T cells, exhibited promoter hypermethylation, and reduced expression in patients with HIV/AIDS (13). *TRAF1* was associated with Vpu-induced apoptosis in HIV-infected T cells (14). Therefore, several researchers started to pay attention to the role of DNA methylation in T-cell apoptosis during HIV infection. In another study, HIV-induced T-cell depletion could be rescued by 5-azacytidine (5azaC), which is a demethylation agent (15). However, few studies have focused on the association between DNA methylation and T-cell apoptosis during HIV infection, especially from the genome-wide transcriptome perspective. Zhang et al. reported an epigenome-wide comparison between HIV-infected and uninfected individuals; however, those authors used total DNA from blood and did not extract CD4^+^ T cells (16).

To explore the interaction between DNA methylation and gene expression in the host genome during HIV infection at the whole-genome scale, especially in terms of T-cell apoptosis, we infected the MT-2 and Jurkat cell lines with HIV-1. We performed both RNA sequencing (RNA-seq) and methylated DNA immunoprecipitation sequencing (MeDIP-seq) to characterize the genome-wide alterations in the transcriptome and methylome between HIV-infected and uninfected T-cell lines. We found that the cross talk between DNA methylation and gene expression was closely related to cell apoptosis during HIV-1 infection. Functional assays were used to verify the effect of DNA methylation on promoters, and siRNA knockdown was further used to assess the effect of the discovered genes on apoptosis. Human primary peripheral blood mononuclear cells (PBMCs) were also investigated in this study. Our data improved the understanding of the molecular mechanism and genetic events underlying the infection of human T cells by HIV-1.

## Materials and Methods

### HIV Infection and Extraction of Genomic DNA From Cells

HIV-1 pNL4-3 was generated by transfection of the pNL4-3 plasmid into HEK 293T cells (which are human embryonic kidney cells 293 containing the SV40 large T-antigen). We infected PBMCs from two normal individuals, as well as two T-cell lines, [i.e., the MT-2 (which is an HTVL-1 transformed human T-cell leukemia cell line) and the Jurkat (which is a T lymphoblastoid derived from acute T-cell leukemia) cell lines, with HIV-1 pNL4-3 at a multiplicity of infection (MOI) of 1 for 5 days]. On average, around 24% Jurkat cells, 39% MT-2 cells, and 23% CD4^+^ T cells in PBMC were infected. The HIV-1 pNL4-3 strain was chosen because it is a representative T-cell-tropic HIV strain that is commonly used in the laboratory. Moreover, pNL4-3 would elicit enhanced methylation compared with the R5-tropic virus. Because X4-tropic (NL4-3) HIV infection in human regulatory T cells (Tregs) could remarkedly increase the expression of DNMT3b, which is a DNA methyltransferase responsible for *de-novo* methylation. This phenomenon was not observed in R5-tropic HIV infected Tregs. Notably, the suppressive function of Tregs was defective when infected by X4-tropic HIV but not by R5-tropic HIV, which could be explained by an increase in Foxp3 methylation in the X4-tropic HIV-infected compared with non-infected Tregs (17, 18). Three independent infection experiments were performed in the P3 facility at the University of Hong Kong. Five days post-infection, the infected cells were harvested for further study. Viral replication was monitored using a p24 ELISA kit. The effect of HIV infection on methylation-related proteins, including DNMT-2s, was determined by western blot analysis. Genomic DNA was extracted from HIV-infected and non-infected cells using the Blood & Cell Culture DNA Mini Kit (Qiagen, Hilden, Germany) according to the manufacturer's protocol, and frozen at −80°C until the DNA methylation analysis.

### Preparation of a Methylated DNA Immunoprecipitation Sequencing Library and Sequencing

MeDIP-seq was used to profile the methylome landscape of HIV-infected and non-infected cell lines. Genomic DNA was sheared into random fragments, and libraries were prepared using the Paired-End DNA Sample Kit (Illumina) according to the manufacturer's instructions. Adaptor-ligated DNA was immunoprecipitated using a specific anti-5-methylcytosine monoclonal antibody. The methylated fraction and the input DNA were purified with ZYMO DNA Clean & Concentrator-5 columns, and were amplified by adaptor-mediated PCR. Amplification quality and quantity were analyzed on an Agilent 2100 Bio-analyzer. Ultra-high-throughput 100 bp paired-end sequencing was performed using the Illumina HiSeq 2000 platform according to the manufacturer's protocols.

### Analysis of Methylated DNA Immunoprecipitation Sequencing Data

The short reads were aligned to the Ensembl GRCh37.75 human reference genome using bowtie2 under default parameters, to obtain a bam file in paired-end alignment mode (19). Bowtie2 builds an index for the genome based on the Burrows–Wheeler Transform algorithm, and bam files were obtained after bowtie2 alignment. The alignment results were sorted based on the aligned positions on the reference genome using samtools sort.

### Detection of Differentially Methylated Regions

The bam files obtained after sorting and removing PCR duplicates were further processed using the R package MEDIPS (20). MEDIPS was used to calculate methylation levels and call DMRs between HIV^+^ and control samples. DMRs were defined as the genomic regions with significantly different methylation levels between HIV-infected samples and uninfected control samples. To find DMRs, we applied a genome-wide sliding-window approach to identify methylation changes. FDR < 0.1 was selected as the threshold for DMR identification (enrichment above the background).

### Annotation of DMRs

To annotate DMRs, the genomic location of transcription start sites (TSSs), exons, gene bodies, and CpG islands on Ensembl GRCh37.75 were used, which were retrieved from BioMart. A DMR was assigned to a functional genomic region (for example a promoter, gene body, or CpG island) if the DMR intersected with a functional genomic region by at least 1 bp. The promoter region was defined as the 1 kb region flanking each side of the TSS, and the intergenic region was defined as the region other than the gene body and promoter region.

### Bisulfite Treatment, Methylation-Specific PCR (MSP), and Bisulfite-Sequencing PCR

Six DMGs (*PDX1, LEF1, TBX3, CPT1A, GATA3*, and *NTRK2*) were selected for bisulfite validation. Three/two bisulfite modified primers were designed to amplify these methylated regions. gDNA from HIV-infected and uninfected Jurkat cells was bisulfite treated using the EZ DNA methylation lightning kit (ZymoResearch), which converts non-CpG-bound cytosines to uracil. Methylation-specific PCR was performed to transcribe uracil to thymine and amplify the targeted region using bisulfite modified and unmodified primers. This was followed by gel electrophoresis to validate amplicon size and ensure the complete conversion of non-CpG-bound cytosines in the gDNA. After obtaining band with a correct size, gel purification was carried out accordingly using the GFX™ PCR DNA and Gel Band Purification kit (Illustra), and purified DNA concentration was measured on a NanoDrop instrument.

Reactions were set up to ligate the purified PCR products into the pCR2.1 vector using the TA Cloning kit (Thermo Fischer Scientific) and transformed into competent *E. coli*. Transformants were spread on an LB-agar plate containing ampicillin and treated with X-gal for clonal selection, in which white colonies represent successful transformation. Colonies were inoculated and grown in a shaking incubator, followed by PCR to validate band size, and sent to Beijing Genomics Institute (BGI) for automatic sequencing.

### Extraction of RNA and Analysis of RNA-seq Data

Total RNA was extracted using a QIAamp RNA Blood Mini kit (Qiagen, Hilden, Germany) and frozen at −80°C until RNA-seq analysis. Ultra-high-throughput 100 bp paired-end sequencing was performed using the Illumina HiSeq 2000 platform according to the manufacturer's protocols. The expression levels of all genes were calculated and DEGs between HIV-infected and uninfected cells were identified using the tophat2-cufflinks pipeline (21). DEXSeq (22) was used to check for the presence of alternative splicing events in HIV^+^ samples. A strict threshold (dispersion < −0.1 and adjust_*p*-value < 0.1) was set for DEXSeq, to ensure the high confidence of the results.

### Gene Function Annotation and Pathway Analyses

The list of DEGs (fold change > 5) was input into DAVID, IPA, and GSEA for function enrichment analysis (23–25). Benjamini < 0.01 was set as the threshold for selecting significantly enriched clusters (KEGG pathway, GO term, disease, and biological functions) for DAVID. The threshold for IPA and GSEA results was *p* < 0.05. The gene function annotation and enrichment analysis was performed using DAVID (24). In this study, analyses of Canonical pathways and Diseases and Biological Functions enrichment were also performed using IPA (25). Function clusters are referred to GO terms or KEGG pathways in this study.

### Quantitative Reverse Transcription PCR

To measure the extent of gene expression, quantitative reverse transcription PCR (RT–qPCR) was carried out for selected genes in HIV-infected Jurkat cells. The procedure involved the use of the Power SYBR Green PCR master mix (ThermoFisher Scientific), primers, and the complementary DNA obtained from the reverse transcription of RNA. A total of eight genes were tested in triplicate for experimental work and analysis: *SMAD3, PDX1, LGALS3, TRAF1, GATA3, RAN, HSP90AB1*, and *HSPA5*. Fluorescence readings from samples were quantified using an Applied Biosystems Viia7 Real-Time PCR system. Data analysis was conducted in Microsoft Excel, and the housekeeping *GAPDH* gene was used for data normalization. Statistical significance of the differences in gene expression between HIV-infected and uninfected Jurkat cells was calculated using an unpaired *t*-test, and error bars were added based on the standard deviation. Significance was set at a *P* < 0.05. GraphPad Prism was used to create graphs and perform statistical calculations. RT–qPCR data were analyzed using the double delta Ct method to calculate the fold changes. The primers used in this experiment are listed in Data Sheet 2: Table S1.

### PCR of the *PDX1* and *SMAD3* Promoters

Oligonucleotide primers were designed and synthesized to amplify a 1,000 bp fragment from the *PDX1* and *SMAD3* promoter regions. The forward primer for *PDX1* was 5′-CGACGCGTGCGGAGAAGCATTTTTCA−3′ and contained the restriction site *Mlu*l, while the reverse primer was 5′-GAAGATCTAGCGAGAGGGGCGGGGCG−3′ and contained the restriction site *Bgl*II. The forward primer for *SMAD3* was 5′-CGACGCGTCCAAAGTCAAGATCAAGA−3′ and contained the restriction site *Mlu*l, while the reverse primer was 5′-CCGCTCGAGGCTCACCTGTAAAATGGG−3′ and contained the restriction site *Xho*l. Genomic DNA was extracted from Jurkat cells using the DNA Mini purification kit (QIAamp) and was used as the PCR template. PCR was performed using Q5 High-Fidelity DNA Polymerase M0491 (NEB). The PCR reaction components (in 25 μl) consisted of 5 μl of 5 × Q5 Reaction buffer, 0.5 μl of 10 mM dNTPs, 2.5 μl of primer mix, 100 ng of template DNA, 0.25 μl of Q5 High-Fidelity DNA Polymerase, and a variable amount of nuclease-free water. The PCR products were analyzed on a 1% DNA agarose gel containing 1 × Gel red. The bands were examined under ultraviolet light and sliced for further DNA recycling. The DNA fragments from the *PDX1* and *SMAD3* promotor regions were then purified using a GFX PCR DNA and Gel Band Purification Kit (Illustra) and stored at −20°C.

### Dual Luciferase Assay

A dual luciferase reporter assay system (Promega, USA) was used to investigate whether Tat and Rev proteins from HIV affect the expression of the putative target genes *PDX1* and *SMAD3*. the promoters of *PDX1* and *SMAD3* were fused to the *fLUC* promoter by cloning into pGL3-basic as a reporter. The sequences of Tat and Rev were cloned into the pcDNA3.1+ vector, to express the respective proteins in cell lines. The luciferase activity of the samples with co-transfection of the pcDNA3.1+ empty vector and the promoter vector were used to assess the basal activity of the promoter in the cell lines. The comparison of the promoter activities with and without Tat or Rev was performed using Holm–Sidak's multiple comparisons test.

### Culture of HEK293T Cells, MT-2, and Jurkat Cell Lines

HEK293T (human embryonic kidney fibroblast) cells were maintained in Dulbecco's Modified Essential Medium High Glucose (Gibco-BRL, Grand Island, NY) supplemented with 10% fetal bovine serum (FBS) and 100 mg/ml antibiotics (penicillin and streptomycin). All cells were cultured under 5% CO_2_ at 37°C. Jurkat and MT-2 cells were cultured in RPMI 1640 medium supplemented with 10% fetal bovine serum (FBS) and 1% penicillin / streptomycin and incubated in a humidified incubator at 37°C under an atmosphere of 5% CO_2_.

### Transfection

Lipofection was carried out using Lipofectamine3000 (Invitrogen) according to the manufacturer's protocol. HEK293T cells were seeded at 8 × 10^4^ cells per well in 24-well plates 24 h before transfection. Vectors containing the HIV protein sequence (pcDNA3.1+-Tat, pcDNA3.1+-Rev, or control pcDNA3.1+ empty vector) were co-transfected with the firefly luciferase constructs (pGL3-PDX1, pGL3-SMAD3, or the pGL3 empty vector control) at a 1:1 ratio (200 ng each). For each transfection reaction, the control plasmid (pRLSV40) containing Renilla luciferase was mixed with the firefly luciferase construct at a 1:200 molar ratio.

### Luciferase Activity Assay

Firefly and Renilla luciferase activities were measured sequentially from a single-cell lysate on a GloMax® 20/20 Luminometer (Promega, USA) using the dual luciferase assay system according to the manufacturer's protocol. Promoter activities were expressed as a ratio of firefly luciferase (Fluc) activity to Renilla luciferase (Rluc) activity. Data were collected from six experiments and the standard deviation was calculated.

## Results

### Differentially Methylated Regions (DMRs) Were Enriched in Specific Genomic Regions

**Figure 1:**
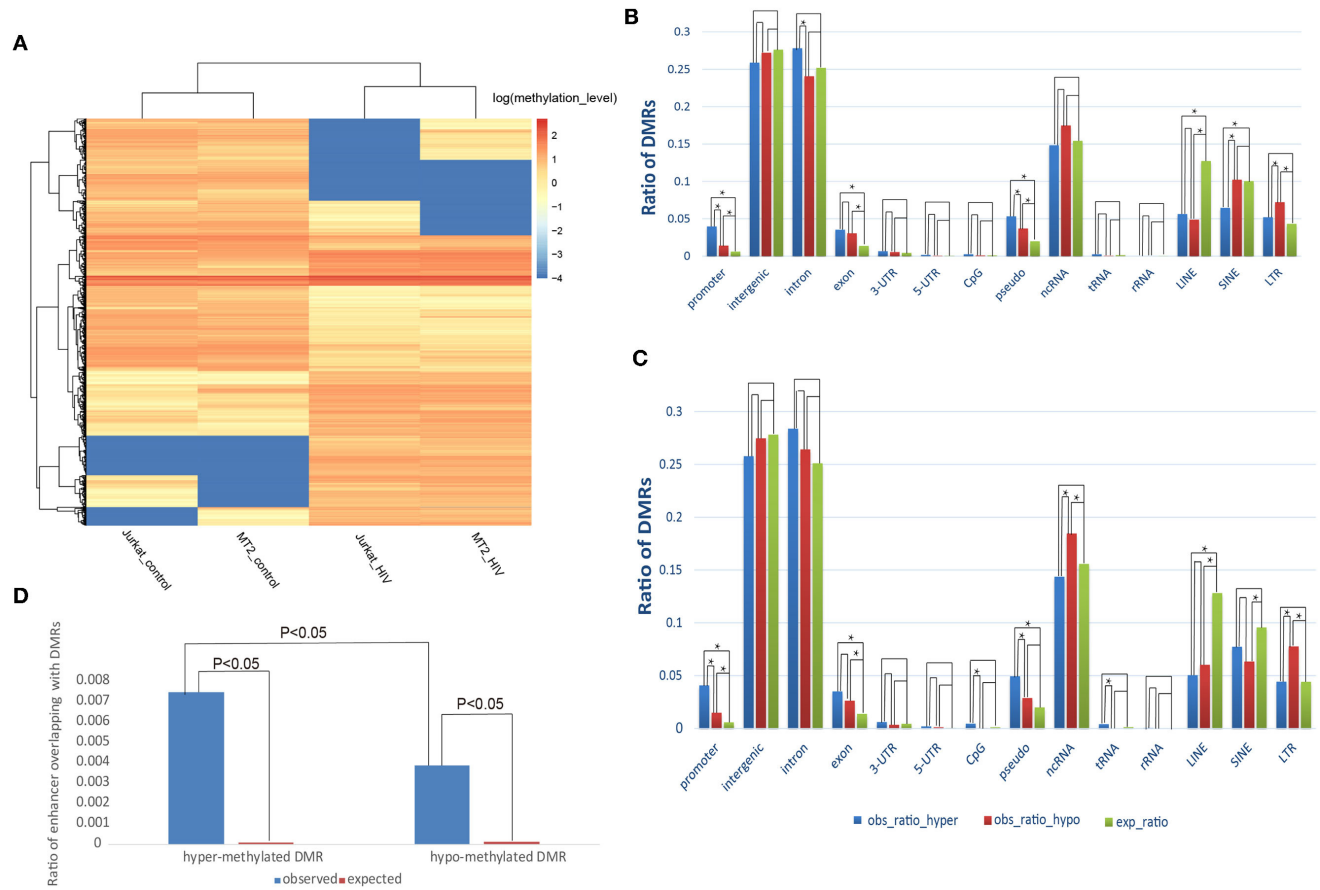
Distribution of the DMRs. **(A)** Heatmap displaying the methylation levels of DMRs in control and HIV^+^ T cells. **(A)** An unsupervised hierarchical clustering analysis (Euclidean distance, complete linkage method) identified two main clusters: control (Jurkat_control and HIV_control) and HIV^+^ (Jurkat_HIV and MT-2_HIV). Log-transformed methylation levels were displayed as a range from low (blue) to high (red). **(B)** Comparison of the observed and expected distribution of DMRs in the MT-2 cell line. **(C)** Comparison of the observed and expected distribution of DMRs in the Jurkat cell line. “obs_ratio_HIV,” observed ratio of DMRs in HIV-infected samples; “obs_ratio_control,” observed ratio of DMRs in the control sample; “exp_ratio,” expected ratio in the genomic background. The asterisk indicates significant differences with *P* < 0.01 between the two bars connected by line segments. **(D)** Change of methylation in enhancers after HIV infection of the MT-2 cell line. Ratio of the overlap of enhancers with DMRs in the MT-2 cell line. The expected ratio was calculated based on a random distribution.

We identified 1,428 hypermethylated and 1,227 hypomethylated DMRs in HIV^+^ samples of the MT-2 cell line compared with the uninfected controls. While for Jurkat cell line, 1,231 hypermethylated and 1,833 hypomethylated DMRs were detected. The DMRs were mainly located in gene body and promoter regions, rather than intergenic regions, despite the fact that nearly half of the human genome consists of intergenic regions (Data Sheet 1: Figure S1), suggesting the important roles of DNA methylation in HIV infection. Moreover, DMRs could distinguish well the HIV^+^ samples from the controls, as suggested by the unsupervised hierarchical clustering (Figure 1A).

Next, we investigated whether the HIV-infection-associated DNA methylation reprograming preferentially occurred in specific genomic contexts. Compared with the random distribution, both hyper- and hypomethylated DMRs were enriched in promoters and exons (Figures 1B,C). Furthermore, the comparison of hyper- with hypomethylated DMRs revealed that hypermethylated DMRs were enriched to a greater extent in promoter regions (Fisher's exact test; *P* = 1.59E−08, MT-2 cell line; *P* = 1.02E−07, Jurkat cell line). This result suggests that, during HIV-1 infection, methylation changes are prone to occur in coding and *cis*-regulation regions.

Considering that aberrant methylation usually occurs in enhancers, we further explored whether the DMRs were enriched toward enhancers. We found that 0.74% of enhancers overlapped with a hypermethylated DMR, whereas only 0.38% overlapped with a hypomethylated DMR in the HIV^+^ MT-2 cell line. These two ratios were significantly higher than the random distribution (*P* < 0.05, chi-squared test), suggesting that methylation changes also tend to occur in enhancer regions. Moreover, similar to that observed for promoter regions, the ratio of enhancer overlapping with hypermethylated DMRs was significantly higher than that of hypomethylated DMRs, suggesting that enhancers tend to gain methylation after infection of T cells with HIV-1 (Figure 1D). In addition, long interspersed nuclear elements (LINEs), long terminal repeat regions (LTRs), and pseudo also showed a tendency toward enrichment of methylation alterations (Figures 1B,C; Data Sheet 3: Supplemental Text). The results presented above suggest that the methylation alterations that follow HIV-1 infection are not random events; rather, the DMRs showed preference for functional genome contexts.

### DMRs Are Enriched for Functional Transcription-Factor-Binding Motifs

We have demonstrated that methylation changes seem to be enriched in both distal enhancers and promoters in T cells in response to HIV infection. Given that enhancers and promoters play an important role in transcriptional *cis*-regulation (26), these findings suggest that DMRs contain *cis*-elements that could be bound by transcription factors, thus playing important regulatory roles in the immune response to HIV-1 infection. To verify our hypothesis, we searched for transcription-factor-binding motifs that were significantly enriched in the hyper- and hypomethylated DMRs. We identified 83 transcription factor binding motifs enriched in hypermethylated DMRs in the MT-2 cell line. The top 10 enriched motifs and their corresponding transcription factors are listed in Data Sheet 1: Figure S2. As methylation changes have been reported to be related to T-cell apoptosis, we specifically focused on apoptosis-associated genes. Notably, nine of the 83 enriched transcription-factor-binding motifs were related to apoptotic processes. The corresponding transcription factors that bound to these motifs were WT1, HIF1A, AHR, EGR1, IRF1, MEF2A, MEF2C, MEF2D, and FOXP1, five of which (WT1, HIF1A, EGR1, IRF1, and MEF2C) have been reported to be associated with HIV-1-induced apoptosis (27–31). Interestingly, these apoptosis-related motifs in hypermethylated DMRs were also enriched in the hypomethylated DMRs. This result suggests that the apoptosis of T cells during HIV infection is the result of aberrant DNA methylation at the binding sites of related transcription factors.

In the Jurkat cell line, the enriched transcription-factor-binding motifs were like those detected in MT-2 samples. Briefly, we found 53 enriched transcription-factor-binding motifs for the hypermethylated DMRs in HIV^+^ samples. Interestingly, 47 out of these factors also appeared among the 83 motifs that were detected in the MT-2 cell line. Moreover, 147 transcription-factor-binding motifs were enriched for the hypomethylated DMRs in HIV^+^ samples, 94 of which were shared with the hypomethylated DMRs in the MT-2 cell line. The top 10 enriched motifs for both methylated and demethylated DMRs are listed in Data Sheet 1: Figure S2.

### Genes With Promoters Overlapping DMRs Were Enriched in Function Categories Related to Transcription Factor Activity and Apoptosis

We considered a gene that overlapped with a DMR (either in the promoter or gene body) for at least 1 base pair (bp) as a differential methylated gene (DMG). We detected 336 and 300 DMGs with hypermethylated DMRs in their promoters in the MT-2 and Jurkat cell lines, respectively. Moreover, we found 219 and 245 DMGs with hypomethylated DMRs in the MT-2 and Jurkat cell lines, respectively. To check the homogeneity of the data obtained from the two cell lines, we verified the number of overlapped DMGs between them. The results showed that 160 DMGs with hypermethylated DMRs in their promoters were shared by both the Jurkat (160/336) and MT-2 (160/300) cell lines. In turn, 71 DMGs with hypomethylated DMRs in their promoters or gene bodies were shared between the two cell lines (71/219 in the MT-2 and 71/245 in the Jurkat cell line) (Data Sheet 1: Figure S3).

We used the Database for Annotation, Visualization, and Integrated Discovery (DAVID) to perform a function enrichment analysis (KEGG pathway analysis and GO term analysis) for DMGs with DMRs in their promoters. As a result, hyper-DMGs were enriched in transcription-factor-related GO terms for the MT-2 cell line (Data Sheet 2: Table S2), among which the top three enriched GO terms were “Protein binding” (*P* = 5.47E−04), “ATPase activity” (*P* = 0.001), and “DNA binding” (*P* = 0.001). For the Jurkat cell line, the top three enriched GO terms were “ATPase activity” (*P* = 6.29E−04), “Transcription factor activity sequence-specific DNA binding” (*P* = 9.17E-04), and “Protein binding” (*P* = 0.001) (Data Sheet 2: Table S3), suggesting that hypermethylation affects transcription factor binding during HIV infection, which is consistent with our previous results. Notably, we also found that a canonical pathway, [i.e., “Induction of Apoptosis by HIV-1,” was enriched for hyper-DMGs (*P* = 0.00054), including the *CYCS, NFKB1, NFKBIA*, and *TNF, TRAF1* genes]. This result suggests that hypermethylation of the promoters of apoptosis-related genes might be a key regulator that leads to T-cell depletion during HIV infection. More specifically, taking *TRAF1* as an example, Saxena et al. showed that TRAF1 degradation could lead to the release of an apoptosis-inducing factor (16). Furthermore, consistent with this finding, our RNA-seq data showed that *TRAF1* was significantly downregulated after HIV-1 infection, suggesting a correlation between gene expression and promoter methylation. To our knowledge, this is the first report to show that DNA methylation plays a role in the TRAF1-related apoptosis in HIV-infected T cells. In addition, the “Cytokine production and biological process” GO term was enriched for the hyper-DMGs in the HIV^+^ Jurkat cell line (*P* = 0.0018). Conversely, for hypo-DMGs (Data Sheet 2: Tables S4, S5), the functional cluster “Transcription factor binding” was also among the top three enriched GO terms (*P* = 4.73E−05, including the *CDKN2A, HEY1, DACT2, HTT, GATA3, HEY2*, and *LEF1* genes).

### Validation of Methylation Alterations Using Bisulfite Sequencing

We performed bisulfite sequencing to validate the DMRs detected between HIV-infected and uninfected Jurkat cells. For this, we selected six DMRs (Table 1) that were located within 1,000 bp upstream of transcription start sites (TSSs). All six corresponding DMGs were functionally related to cell apoptosis and displayed a correlation between their gene expression level and promoter methylation. Primers were designed to capture the DNA sequences of the DMR regions (Data Sheet 2: Table S6). Encouragingly, all six selected DMRs were successfully validated by bisulfite sequencing. An example that illustrates the consistency between the MeDIP-seq and bisulfite sequencing results is given in Data Sheet 1: Figure S4.

**Table 1:** DMRs selected for bisulfite sequencing.

**Gene name**	**Chromosome of DMR**	**Start of DMR**	**End of DMR**	**hyper-/hypo-methylated in HIV^**+**^ sample**	**Region where DMR was located**
*PDX1*	chr13	28493501	28493900	Hyper	promoter
*GATA3*	chr10	8101301	8101600	Hypo	promoter
*LEF1*	chr4	109087301	109087400	Hypo	promoter
*TBX3*	chr12	115121601	115121700	Hyper	promoter
*CPT1A*	chr11	68607601	68607800	Hypo	promoter
*NTRK2*	chr9	87284101	87284300	Hyper	promoter

### Identification of Differentially Expressed Genes (DEGs) Between HIV-Infected and Uninfected Samples

Previous studies have found that the expression of genes related to the immune response and cell death undergo dramatic changes in T cells after infection with HIV-1 (32). To identify novel genes with altered expression levels, as well as to determine the effect of differential DNA methylation on gene expression after HIV-1 infection, in T cells, we profiled the transcriptome of the two cell lines using RNA-seq analysis of HIV^+^ samples and uninfected controls for the two cell lines. We identified an average of 1,633 upregulated and 2,142 downregulated genes using the two sample sets.

Next, we explored the functional gene clusters involved in HIV infection using DAVID. Regarding DEGs that were upregulated after HIV infection, “Primary immunodeficiency” was among the top three enriched functional clusters for both MT-2 and Jurkat samples (Benjamini FDR = 2.73E−10 in the MT-2; Benjamini FDR = 2.75E−04 in the Jurkat cell line) (Data Sheet 2: Tables S7, S8), suggesting that HIV effectively infected the T cells in our experiments and consequently caused immunodeficiency. Moreover, a GSEA analysis showed that the genes that were upregulated in HIV^+^ Jurkat cells in our study significantly overlapped with the downregulated genes when comparing naive T cells with central memory T cells in another study (*P* = 0.0096) (Data Sheet 2: Table S9), confirming that HIV infection affects the central memory T cells through transcriptional alterations (33). Regarding the downregulated genes, the GO term “Immune response” was among the top three enriched function clusters in both sample sets (Benjamini FDR = 3.51E−13 in MT-2 cells; Benjamini FDR = 3.98E−11 in Jurkat cells) (Data Sheet 2: Tables S10, S11), suggesting that the infected cells had initiated the immune defense against HIV, as expected, which also proved the credibility of our data. Notably, we found that the downregulated DEGs were significantly enriched in three apoptosis-related GO terms, including “Apoptotic process” (Benjamini FDR = 2.54E−06 in MT-2 cells; Benjamini FDR = 2.83E−06 in Jurkat cells), “Regulation of apoptotic process” (Benjamini FDR = 2.08E−07 in MT-2 cells; Benjamini FDR = 4.34E−05 in Jurkat cells), and “Positive regulation of apoptotic process” (Benjamini FDR = 2.75E−05 in Jurkat cells) (Data Sheet 2: Tables S10, S11). This suggests that HIV infection modulates the apoptosis of T cells by reducing the expression of several key regulators, which was consistent with the findings reported by previous studies (3, 34, 35). Many of the genes involved in this process were reported for the first time as participating in the apoptotic process in HIV-1 infection, which warrants further investigation.

**Figure 2:**
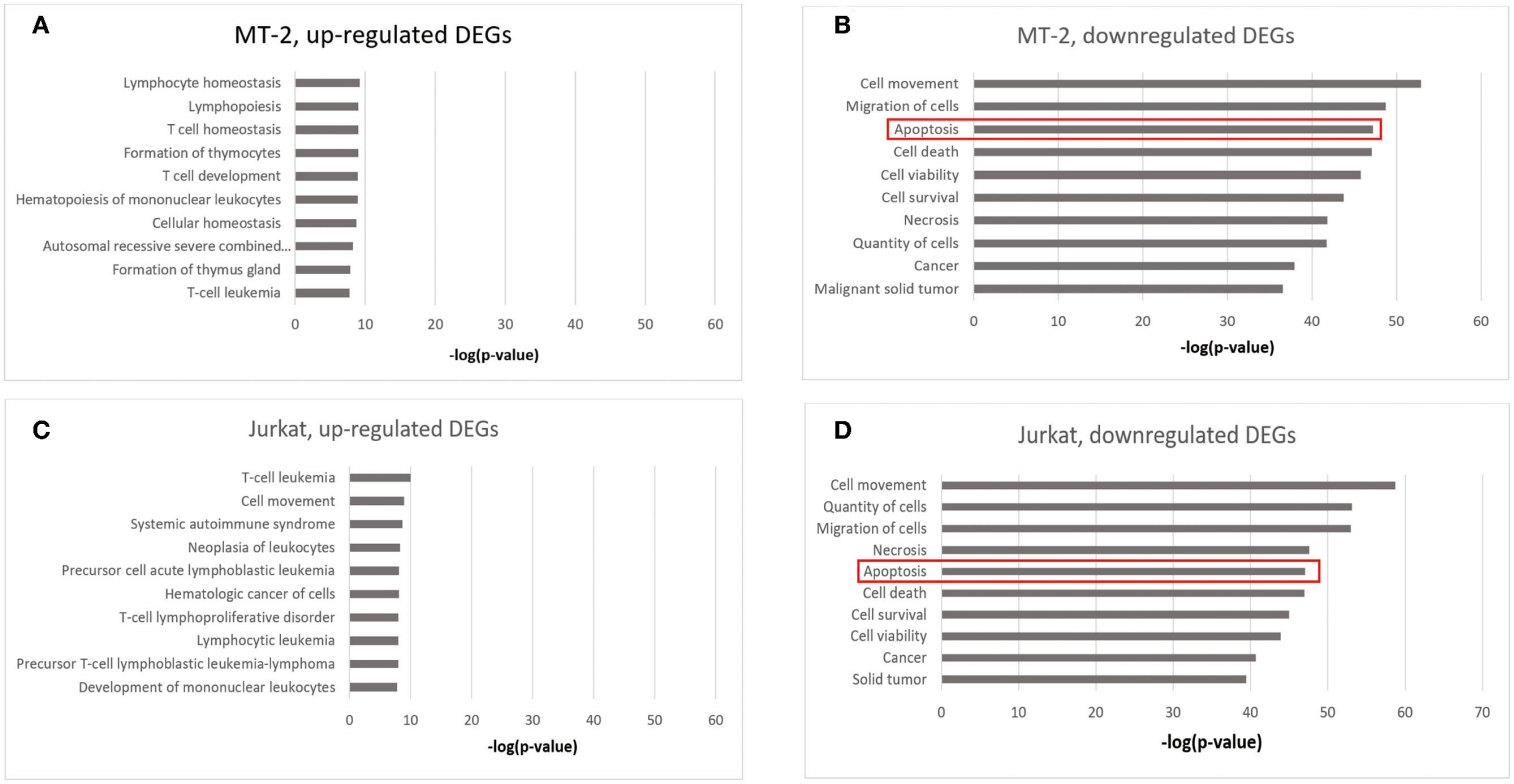
Enrichment analysis of disease and biological functions among the DEGs using IPA. **(A)** DEGs upregulated in HIV^+^ samples from the MT-2 cell line; **(B)** DEGs downregulated in HIV^+^ samples from the MT-2 cell line; **(C)** DEGs upregulated in HIV^+^ samples from the Jurkat cell line; **(D)** DEGs downregulated in HIV^+^ samples from the Jurkat cell line. **P < 0.01; ***P < 0.001.

Notably, when performing disease and biological function enrichment analyses using another tool [i.e., Ingenuity Pathway Analysis (IPA)], we found that the downregulated genes in both cell lines were enriched in the “apoptosis” biological function, and that 345 apoptosis-related genes overlapped between the MT-2 (449 genes) and Jurkat (409 genes) cell lines. The high consistency indicates the high confidence level of our data (Figure 2; Data Sheet 2: Table S12). Regarding the canonical pathway analysis, the top three enriched pathways for downregulated genes were “T helper cell1 (Th1) and T helper cell (Th2) Activation Pathway” (*P* = 4.16E−10 in Jurkat cells), “Th1 Pathway”(*P* = 2.78E−8 in Jurkat cells), and “Th2 Pathway” (*P* = 4.14E−8 in Jurkat cells), all of which are closely related to the function of CD4^+^ T cells (Data Sheet 1: Figure S5), thus supporting the credibility of our data. To assess the presence of transcriptional regulators of the DEGs after HIV-1 infection, we pooled the upregulated and downregulated DEGs together for upstream regulator prediction. In both cell lines, *TNF* (*P* = 3.25E−53 in MT-2 cells; *P* = 2.52E−46 in Jurkat cells) was predicted to be the upstream regulator responsible for the differential expression of the genes (Data Sheet 2: Tables S13, S14). It has been reported that TNF activation affects the replication of HIV-1 (36). Moreover, *TNF* was reported to be involved in apoptosis through the STING signaling pathway (37). Given that *TNF* was also downregulated and exhibited hypermethylation on its promoter region in our data, we assumed that *TNF* was inhibited by HIV-1 to facilitate its replication during the infection of T cells.

Subsequently, we investigated the process of alternative splicing during HIV infection. In the Jurkat cell line, we identified 282 genes that contained differentially expressed exons on their transcripts, among which only 110 genes were differentially expressed. Therefore, the remaining 172 genes were alternatively spliced without expression changes. To investigate further the cellular functions affected by these changes, we used these 282 genes to perform a functional enrichment analysis, and found that the term “apoptotic process” was enriched in this sample (*P* = 2.20E−03, Data Sheet 2: Table S15). These results suggest alternative splicing as an additional mechanism that may contribute to T-cell apoptosis during HIV infection. Additional details of the alternative splicing results are provided in Data Sheet 3: Supplemental Text and Data Sheet 1: Figure S6.

### Experimental Validation of the RNA-seq Results

**Figure 3:**
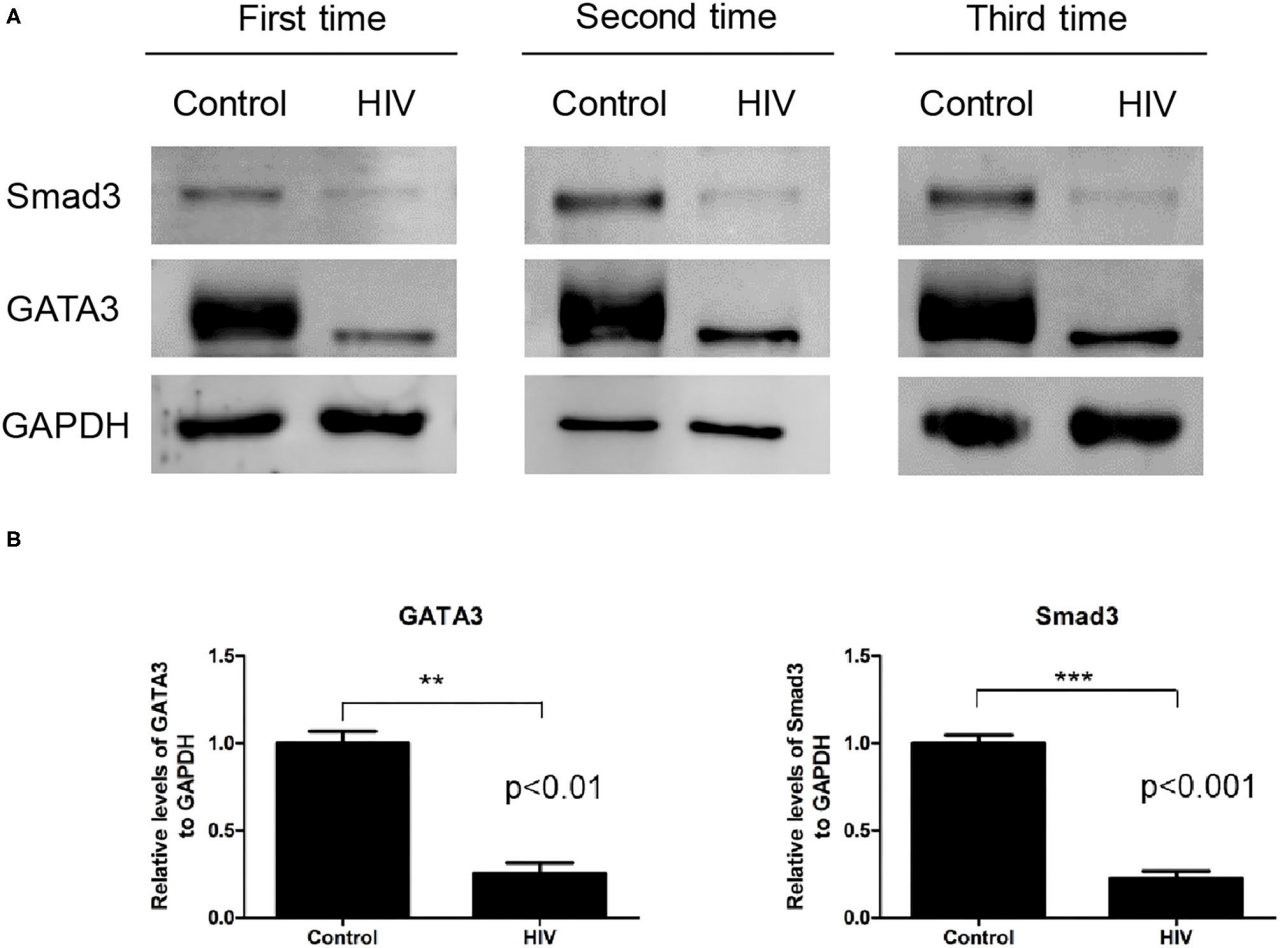
Levels of the Smad3 and GATA3 proteins in Jurkat cells infected by HIV and in the mock control. **(A)** Western blot analysis of Smad3 and GATA3 on human Jurkat T cells infected by HIV and in the mock-infection control. The results of all three replicates are shown. GAPDH was used as the normalization control. **(B)** Relative expression levels of the Smad3 (ab28379) and GATA3 (ab199428) proteins against that of GAPDH, as determined by immunoblotting. Compared with the control sample, the HIV sample exhibited a significantly lower expression of Smad3 (*P* < 0.001) and GATA3 (*P* < 0.01) (data were presented as the mean ± standard error of the mean. Statistical significance was set at *P* < 0.05).

We selected eight DEGs to perform quantitative reverse transcription PCR (RT–qPCR) validation: *SMAD3, PDX1, LGALS3, TRAF1, GATA3, RAN, HSP90AB1*, and *HSPA5*. These genes were related to apoptosis and selected based on strong proapoptotic or antiapoptotic properties in both Jurkat and MT-2 cells, as assessed by the gene expression data. The RT–qPCR results yielded similar patterns to those obtained from our RNA-seq data for the selected genes (validation rate = 100%) (Data Sheet 1: Figure S7). These results increased the confidence of the DEG results presented above. Subsequently, we performed a western blot analysis to confirm the RT–qPCR results. We assessed the effect of HIV infection on the expression of the Smad3 and GATA3 proteins in Jurkat T cells and found that the two proteins were significantly downregulated after HIV infection (Figure 3).

### Correlation Between DNA Methylation and Gene Expression in HIV-Infected Cells

**Figure 4:**
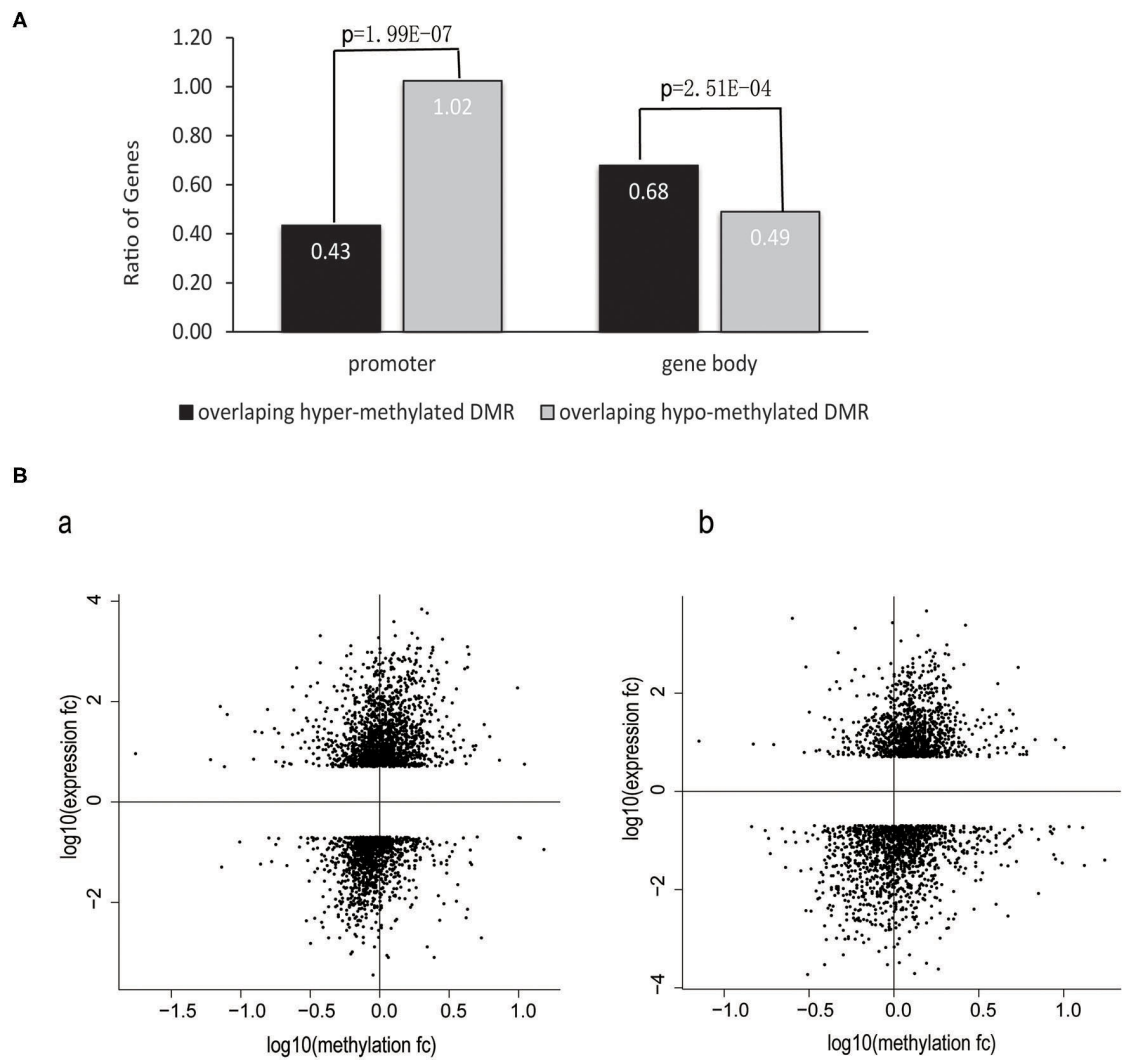
Correlation between changes in gene expression and DNA methylation in promoters and gene bodies. **(A)** The ratio of the number of upregulated genes divided by the number of downregulated genes in terms of the different annotation categories of DMRs. The chi-squared test was used in this analysis. The data obtained from the MT-2 and Jurkat cell lines were combined. **(B)** Gene-body DNA methylation was positively correlated with gene expression levels. Scatter plot showing the fold change in the methylation level of gene bodies against the fold change of gene expression levels for all DEGs between HIV^+^ and control T cells. Gene-body regions were defined as the genomic region starting from the transcription start site to the last base of the gene. Gene expression was correlated positively with gene-body methylation in the (a) MT-2 cell line (Spearman's rank correlation *r* = 0.30, *P* < 2.2e−16) and (b) Jurkat cell line (Spearman's rank correlation *r* = 0.28, *P* < 2.2e−16). Genes exhibiting increased gene expression and gene-body methylation are located in the upper-right quadrant, whereas other genes that became transcriptionally silenced by reduced methylation in HIV^+^ samples are located in the lower-left quadrant.

DNA methylation in the promoter of a gene is a repressive epigenetic modification that has been shown to affect transcription negatively (38–40). Such regulation mechanisms have also been observed in HIV-infected T cells (6, 41, 42). However, few studies have focused on the whole genome to investigate the global correlation between DNA methylation and gene expression in HIV-infected cells. To address this issue, we calculated the odds ratio, which was defined as the number of upregulated genes divided by the number of downregulated genes (number_of_upregulated_genes/number_of_downregulated_genes). DMGs were divided into two categories based on the location of DMRs: overlapped with promoters or gene bodies. The ratio was calculated, respectively for each category of genes. The results showed that the ratio was significantly lower in the category of genes with promoters that overlapped with hypermethylated DMRs vs. those that overlapped with hypomethylated DMRs (0.43 vs. 1.02, *P* = 1.99E−07, chi-squared test); (i.e., a higher proportion of genes were downregulated among the category of genes with promoters that were hypermethylated than among the category of genes with hypomethylated promoters, suggesting that hypermethylation in promoters is more likely to decrease the gene expression level). Conversely, genes that overlapped with hypermethylated DMRs in the gene body tended to be upregulated (0.68 vs. 0.49, *P* = 2.51E−04, chi-squared test), suggesting that DNA methylation in gene bodies is associated with the upregulation of gene expression (Figure 4A).

To investigate further the correlations between gene expression and DNA methylation for the DEGs in T cells during the process of HIV infection, we performed a statistical correlation test between the fold changes in DNA methylation and in gene expression. DNA methylation changes in gene bodies exhibited a weak but significantly positive correlation with gene expression changes in both the MT-2 (Spearman's rank correlation *r* = 0.3, *P* < 2.2e−16) and Jurkat (Spearman's rank correlation *r* = 0.28, *P* < 2.2e−16) cell lines (Figure 4B). This positive correlation between DNA methylation and gene expression supports previous reports of the role of gene-body methylation in various processes (43, 44). Our study demonstrated for the first time the whole-genome-scale correlation between DNA methylation and gene expression in HIV-infected cells.

### Overlap Between DEGs and DMGs Was Enriched in Apoptosis Related Biological Functions

We further investigated the intersection of the expression and methylation data sets to examine the overlap between DEGs and DMGs. As a result, we identified 831 genes that were shared by DEGs and DMGs (406 genes in the MT-2 sample and 425 genes in the Jurkat sample).

**Figure 5:**
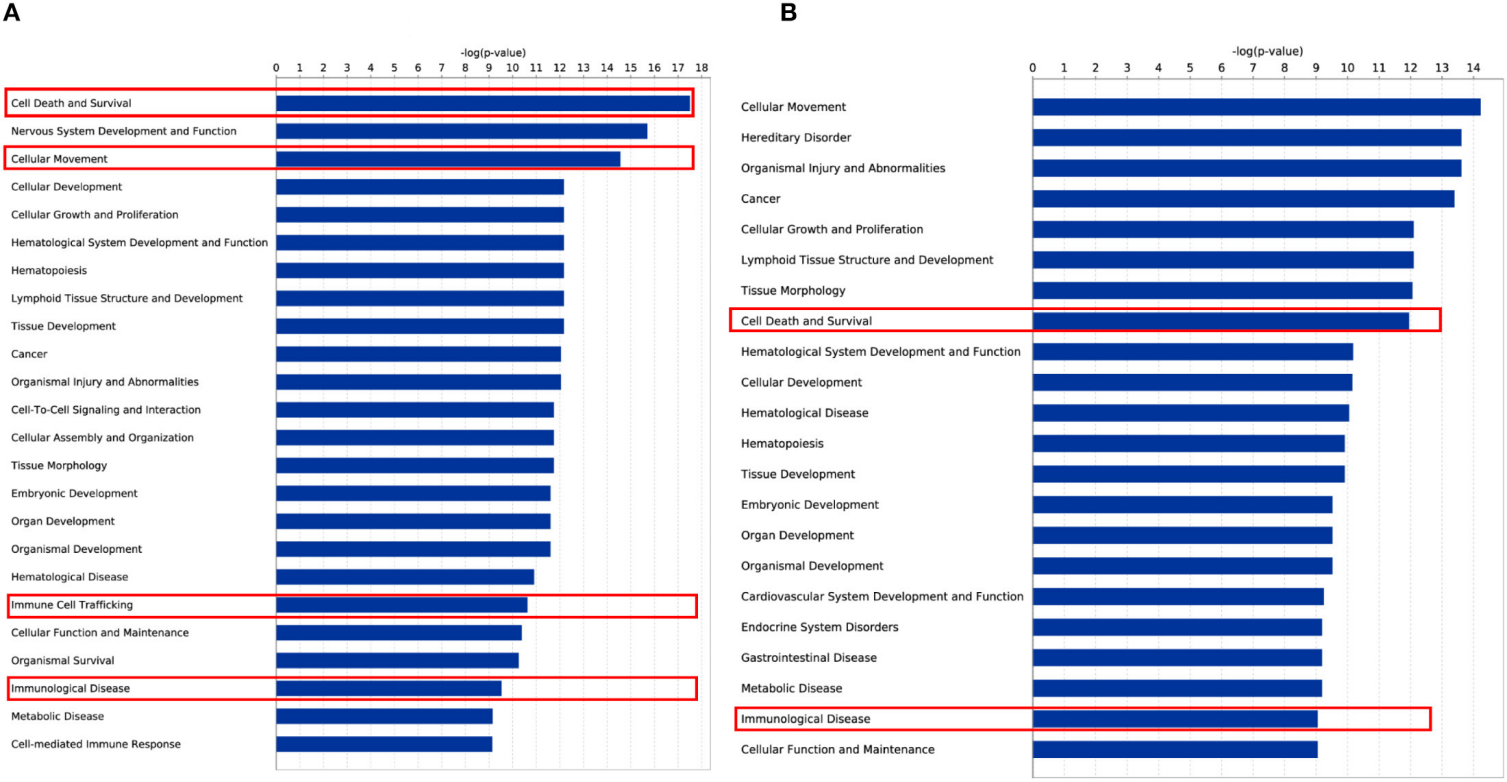
Disease and biological function enrichment for overlap of DEG and DMG. **(A)** Disease and biological function enrichment for overlap of DEG and DMG in the MT-2 cell line. **(B)** Disease and biological function enrichment for overlap of DEG and DMG in the Jurkat cell line.

To check the functional relevance of the DEGs controlled by DNA methylation, the overlap between DEGs and DMGs was input into IPA for disease and biological function enrichment analysis. “Cell Death and Survival,” “Cellular Movement,” “Cellular Growth and Proliferation,” “Immunological Diseases,” “Immune Cell Trafficking,” and “Cellular Development” were significantly enriched for both cell lines (Figure 5). Notably, the “apoptosis” clusters were significantly enriched in the “Cell Death and Survival” category. This suggests that HIV-1 affects T-cell movement, growth, and development and induces apoptosis by altering DNA methylation to regulate gene expression. Moreover, in both MT-2 (*P* = 2.11E−08) and Jurkat (*P* = 3.24E−08) cells, the TGFB1 growth factor is predicted to be one of the top 3 upstream regulators of the shared genes of DEGs and DMGs (Data Sheet 2: Tables S16, S17). It has been reported that long-term overproduction of the anti-inflammatory growth factor TGFB1 is a major cause of immunosuppression in HIV infection (45). The interactions between TGFB1 and DEGs/DMGs are provided in Data Sheet 1: Figures S8, S9. Here, we proposed that *TGFB1* might play an important role in the regulation of DNA methylation and gene expression in HIV infection despite its absence among the DEGs and DMGs.

Based on the collective results presented above, we proposed a list of apoptosis-related genes with high confidence for future research. The expression of the genes included on this list was either negatively regulated by DNA methylation in promoter regions or positively regulated by DNA methylation in gene-body regions. Most of the genes on the list exhibited similar patterns of epigenetic and transcriptional alterations in the two HIV-infected samples (Table 2). To the best of our knowledge, all of the listed genes were reported for the first time to be involved in T-cell apoptosis during HIV infection *via* methylation and transcription regulatory mechanisms. A detailed description and discussion of these genes is provided in Data Sheet 3: Supplemental Text.

**Table 2:** Candidate genes related to apoptosis for future research.

**Gene**	**Gene expression in HIV^**+**^ MT-2 cell**	**Gene expression in HIV^**+**^ Jurkat cell**	**DNA methylation in HIV^**+**^ MT-2 cell**	**DNA methylation in HIV^**+**^ Jurkat cell**	**Description**
*LEF1*	Up	Up	hyper-DMR in genebody	hypo-DMR in promoter	lymphoid enhancer binding factor 1
*CDKN2A*	Down	Down	hypo-DMR in genebody		cyclin dependent kinase inhibitor 2A
*HIPK2*	Down	Down	hypo-DMR in genebody	hypo-DMR in genebody	homeodomain interacting protein kinase 2
*GATA3*	Up	Up	hypo-DMRin promoter	hypo-DMR in promoter	GATA binding protein 3
*SMAD3*	Down	Down	hypo-DMR in genebody	hypo-DMR in genebody; hyper-DMR in promoter	SMAD family member 3
*APP*	Down	Down	hypo-DMR in genebody	hypo-DMR in genebody	amyloid beta precursor protein
*LGALS3*	Down	Down	hyper-DMR in promoter		galectin 3
*MGMT*	Up	Up	hyper-DMR in genebody	hyper-DMR in genebody	O-6-methylguanine-DNA methyltransferase
*RYR2*	Down	Down	hypo-DMR in genebody	hypo-DMR in genebody	ryanodine receptor 2
*NLRP3*	Down	Down	hypo-DMR in genebody	hypo-DMR in genebody	NLR family pyrin domain containing 3
*TRAF1*	Down	Down	hyper-DMR in promoter		TNF receptor associated factor 1
*TNF*	Down	Down	hyper-DMR in promoter		Tumor necrosis factor
*TBX3*	Down	Down	hyper-DMR in promoter	hyper-DMR in promoter	T-Box Transcription Factor
*CPT1A*	Up	Up		hypo-DMR in promoter	Carnitine Palmitoyltransferase 1A
*PDX1*	Down	Down	hyper-DMR in promoter	hyper-DMR in promoter	Insulin Promoter Factor 1
*NTRK2*	Down	Down		hyper-DMR in promoter	Neurotrophic Receptor Tyrosine Kinase 2

“*Up” means upregulated; “Down” means downregulated*.

### Demethylation Treatment to Confirm the Effect of Methylation on Gene Expression

To confirm the effect of DNA methylation on the expression of selected genes from Table 2, we treated the HIV-infected T cells with 5-azaC, which has a broad-spectrum genome-wide demethylation effect. We then assessed the gene expression and the methylation of the promoter regions of these genes. We found that many genes were upregulated by DNA demethylation. The promoter of *LGALS3* was hypomethylated by 16.86-fold after 5-azaC treatment, while its expression level was upregulated by 211-fold. Similarly, the methylation level on the promoter of *TRAF1* was decreased by 7.92-fold, while the expression of *TRAF1* was increased by 19.58-fold. Similar effects could also be observed for *NLRP3, APP, TBX3*, and *NTRK2* (Data Sheet 2: Table S18).

### Effects of HIV Proteins on PDX1 and SMAD3

**Figure 6:**
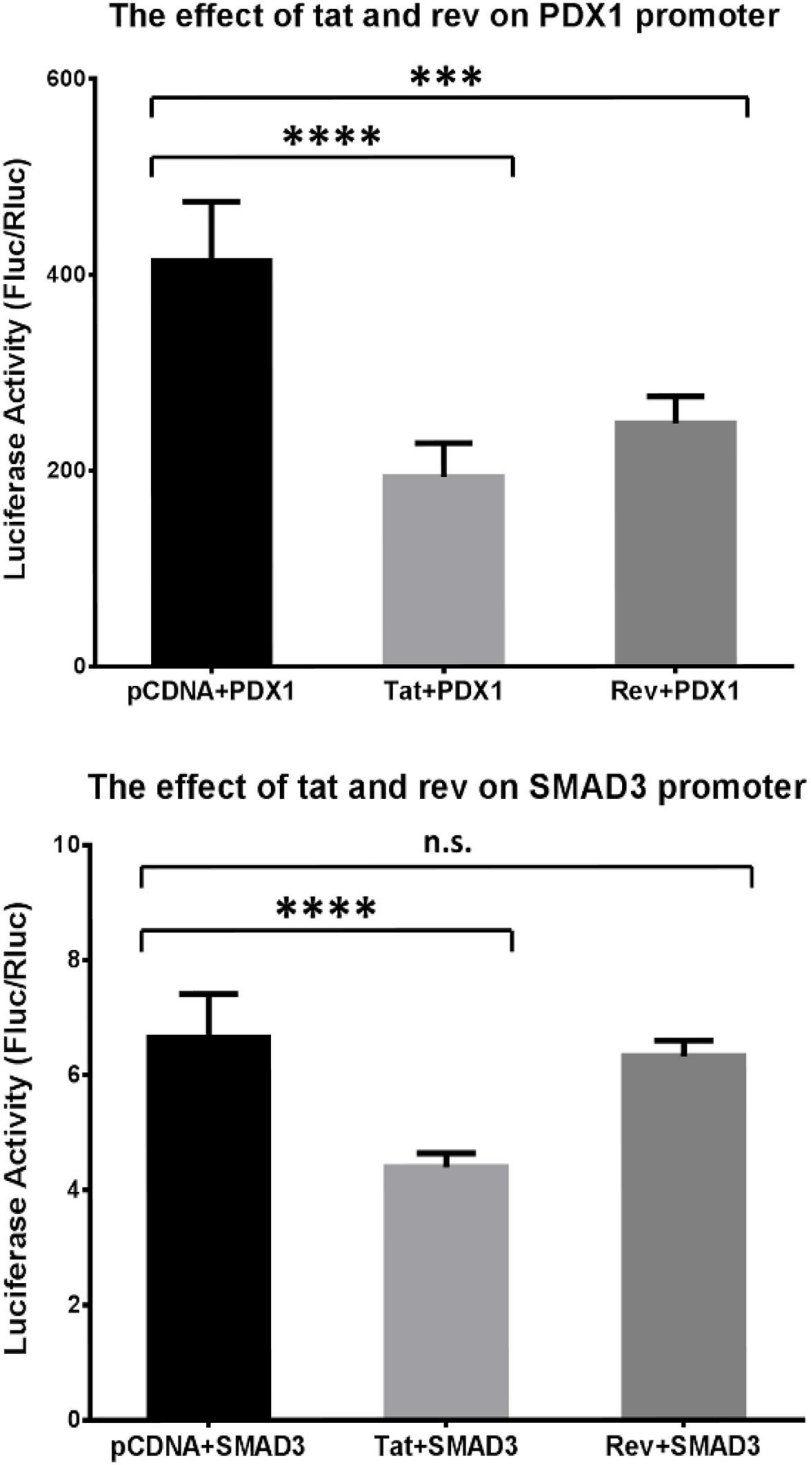
Analysis of the effect of Tat and Rev on *PDX1* and *SMAD3* promoter activity. Results of the promoter gene reporter assay. The luciferase activity was calculated based on the Fluc:Rluc ratio. The promoter vector (*PDX1* or *SMAD3*) was transfected into HEK293T cells, together with the empty pCDNA3.1+ vector, a Tat-expressing vector (Tat), and a Rev-expressing vector (Rev). The experiments were repeated six times. Holm–Sidak's multiple comparisons test. ***P < 0.001, ****P < 0.0001. N.s., non-significant.

We then asked whether the methylation changes in the host genes were induced by the HIV-1 protein. We selected *PDX1* and *SMAD3* from Table 2 for the luciferase assay. The activity of the *PDX1* promoter was significantly decreased in the presence of Tat or Rev, while the promoter activity of SMAD3 was significantly decreased in the presence of Tat (Figure 6). Based on these findings, we speculated that these two HIV proteins specifically regulated DNA methylation in the promoter and, therefore, affected the expression of these two host genes. Although SMAD3 can regulate the HIV LTR in human astrocytes (46), and MH2 domain of SMAD3 can decrease the levels of the Tat-induced activation of MCP-1 and several other cytokines and chemokines in astrocytic cells (47), the effect of these two HIV proteins on the transcriptional regulation of *PDX1* and *SMAD3* through promoter methylation has not been reported.

### Effects of the Candidate Genes on T-cell Apoptosis

To determine the effect of methylation-regulated genes on T-cell apoptosis, *CPT1A, LEF1, GATA3, RCOR2*, and *ADM* were selected for siRNA knockdown. The treatment of Jurkat cells separately with a specific siRNA to these five genes and with a control siRNA, we found that GATA3 promoted cell viability and reduced the number of cells in the G_2_ phase, while CPT1A also had a modest effect on cell viability (Data Sheet 1: Figure S10A). Next, an annexin V assay was used to assess the effects of the candidate genes on apoptosis. We found that knockdown of CPT1A led to a significant reduction in early and late apoptotic cells, while RCOR2 only reduced the number of late apoptotic cells (Data Sheet 1: Figures S10B, S11). It is possible that the apoptotic process triggered by HIV in T cells is very complicated, thus implying that individual genes have a limited effect on apoptosis.

### Further Investigations Using Primary Peripheral Blood Mononuclear Cells (PBMCs)

To investigate further the applicability of our results to human primary cells, PBMCs from normal individuals were infected with HIV. The detection of DEGs in uninfected and HIV-infected cells showed that *CPT1A, TNF*, and *LEF1*, which were among the promising genes on the candidate gene list, also showed differential expression with a similar trend (Data Sheet 2: Table S19). This suggests that these genes are important for HIV infection not only in cultured cell lines, but also in primary human cells.

## Discussion

Although impressive achievements have been obtained regarding the roles of epigenetic regulation in the T cell response after HIV-1 infection, few studies have focused on the genome-wide methylation profiles in T cells before and after HIV-1 infection. In this study, we investigated the differential methylation profiles, as well as the differential gene expression profiles, between HIV-infected and uninfected cells at the whole-genome scale. The results of our analysis were confirmed by experimental validation, including RT–qPCR, BS-seq, and western blotting. Treatment with a demethylation agent was also used to confirm the effect of methylation on gene expression. As a result, candidate genes implicated in the process of HIV infection were identified. Their interaction with HIV proteins was further proven by luciferase assay and their effects on apoptosis were assessed by siRNA knockdown.

Most of the DMRs identified here were located in gene-body regions. However, in a previous study, we performed whole-genome-scale methylation profiling in a pair of twins, one of whom was HIV^+^, and found that most of the DMRs were located in promoter regions (6), which might be attributed to the different nature of cell lines and clinical samples. The enrichment of DMRs in promoters and enhancers suggests that methylation alterations may affect transcription-factor binding, as many transcription-factor-binding sites (TFBSs) were enriched in the DMRs that overlapped with promoters and enhancers. In support of our results, epigenetic changes in enhancer regions of the human genome have been linked with HIV infection. Bachelerie et al. reported that NF-κB induction could render HIV enhancer activity sustainable in monocytes (48). This finding of DMR enrichment toward TFBSs was strongly supported by the function enrichment analysis of DMGs and DEGs, in which DMGs with both hyper- and hypomethylated DMRs were enriched in the transcription-factor-related function clusters, such as “protein binding,” “transcription factor activity,” and “transcription factor binding.” Consistent with our results, recent studies have shown that methylation of TFBS leads to the dysregulation of transcription by blocking the binding sites of transcription factors (49, 50). PU.1 motifs were over-represented in genes with significantly different methylation levels during the methylation that occurs in monocyte-to-osteoclast differentiation, indicating the role of methylation in the regulation of gene expression by affecting TFBS binding (51).

The DMGs were significantly enriched in several function clusters including KEGG pathways and GO terms for both the MT-2 and Jurkat cell lines, among which the most prominent function clusters for hypermethylated genes were “viral carcinogenesis” in the KEGG pathway analysis and “ATPase activity” in the GO term analysis. In many types of cancers related to viral protein induction, such as liver cancer and cervical cancer, lysine acetyltransferase and lysine methyltransferase interact with cellular DNMT-2s, leading to the dysregulation of methylation in the genome of tumor cells. This may explain why DMGs in our data were enriched in viral carcinogenesis in the KEGG pathway analysis. The “ATPase activity” GO term was enriched among DMGs in our study. Previous studies also reported the association between HIV infection and ATPase activity. Schachter et al. showed that increasing ATPase activity or decreasing extracellular concentrations of ATP contributed to HIV-1 infection (52). Zhang et al. performed a genome-wide comparison of DNA methylation between HIV-1-infected and uninfected patients (16). The differences of the results of Zhang et al. and our study could be explained by the fact that those authors measured the methylation levels in total blood cells, while here we measured the methylation levels in pure CD4^+^ T cells.

RNA-seq was performed to investigate the altered expression profiles on the whole-genome scale after infection of T cells with HIV-1. Function clusters including apoptosis-related pathways, primary immunodeficiency, and immune response were significantly regulated after HIV-1 infection. The DEGs involved in the “immune response” function cluster included *SUSD2, TLR1, TNFSF14, TNFSF13, TNFSF12, HLA-DMB, TLR6*, and *HLA-DMA*, which warrant future investigation. For example, the surface level of LIGHT and TNFSF could be enhanced by the expression of the Nef HIV-1 protein. The enhancement was dependent on the sequence of dileucine in the Nef protein (53, 54). *HLA-DMB* was reported as a candidate gene for Kaposi's sarcoma associated with AIDS complications through SNP screening (55). In the enrichment analysis of the disease and biological functions of DEGs, in addition to apoptosis, the “Cell movement” and “Migration of cells” functions were also identified, indicating that cell mobility (including immune cell trafficking) was significantly affected after the infection of T cells with HIV-1. We then used PBMCs to validate our results in primary cells and found that not all DEGs detected in cell lines were detected in PBMCs. This might be attributed to the fact that PBMCs are not pure T cells; rather, they consist of lymphocytes (T cells, B cells, and NK cells) and monocytes.

Regarding the cross talk between DNA methylation and gene expression, we found that, generally, gene expression was negatively correlated with DNA methylation in promoters and positively correlated with DNA methylation in gene bodies. Consistent with our result of activation of the “protein kinase A signaling pathway,” the HIV-1 Vpr protein was able to arrest the cell cycle and further cause the depletion of T cells after being phosphorylated by protein kinase A (56). However, the studies that focused on the role of methylation on protein kinase A signaling in HIV-1 induced T-cell death are limited. In addition, the “Induction of Apoptosis by HIV-1” canonical pathway was enriched in this analysis, thus supporting the key role of both DNA methylation and gene expression in HIV-1-induced cell death.

In our study, we investigate the NL4-3 HIV strain, a X4-tropic HIV-1 which is associated with faster disease progression. Nevertheless, in addition to this X4-tropic strain, there is also a R5-tropic strain for HIV-1. The prevalence of the two tropic strains are compatible (57). Therefore, our study cannot represent the entire HIV-1 isolates. Further investigation on the DNA methylation and transcriptome profiles in T cells infected with CCR5 tropic HIV-1 is desirable. Besides, we use cell lines to investigate the host transcriptional and epigenetic alterations during HIV Infection. However, cell lines may change in both genetic and phenotypic aspects after multiple passages and may not mimic the real situation of primary CD4+ T cells. Therefore, further study should be conducted on primary CD4+ T cells.

We analyzed the RNA-seq and MeDIP-seq data obtained from HIV-1-infected and uninfected T cells and identified functional genomic regions that are prone to differential methylation. The cellular processes and pathways affected by HIV-1 infection were also obtained at both the epigenetic and transcriptomic level. Importantly, we proposed a list of novel candidate genes that may be involved in the T-cell apoptosis induced by HIV infection and may provide insights regarding the changes that occur at both the transcriptomic and epigenetic level in T cells in response to HIV infection. Consistent with the sequencing results, the luciferase assay showed that the activity of the *PDX1* and *SMAD3* promoters was significantly decreased in the presence of Tat or Rev. This could be viewed as one of the major contributions of this study. Further investigation aimed at clarifying the functional significance of this correlation between DNA methylation and gene expression is desirable. These candidate genes may further contribute to the improvement of the diagnosis and treatment of AIDS in the future. In summary, this study provided a resource about the dynamic changes of gene expression and DNA methylation that occur during the infection of T lymphocytes with HIV, which will not only promote the understanding of this molecular process, but also aid in the discovery of new biomarkers that can be used for therapy and diagnosis.

## Data Availability Statement

The original contributions presented in the study are publicly available. This data can be found here: https://www.ncbi.nlm.nih.gov/; Accession ID: PRJNA601273, SRP241980.

## Ethics Statement

This study obtained approval from the Joint Chinese University of Hong Kong-New Territories East Cluster Clinical Research Ethics Committee. The ethics approval for the collection of PBMC has been obtained from the Institutional Review Board of the University of Hong Kong/Hospital Authority Hong Kong West Cluster (HKU/HA HKW IRB). Written informed consent to participate in this study was provided by the participants' legal guardian/next of kin.

## Author Contributions

XZ analyzed the data of the present study and was a major contributor in writing the manuscript. ST, XZ, and MS reviewed and edited the manuscript. JT, LK, CC, LL, JP, CL, YL, and LW performed the wet lab experiments. ST and ZC made a conceptualization for the project. All authors read and approved the final manuscript.

## Conflict of Interest

The authors declare that the research was conducted in the absence of any commercial or financial relationships that could be construed as a potential conflict of interest.

## References

[1] Organization WH Summary of Global HIV Epidemic. (2018). Global Health Observatory (GHO) data (2019).

[2] Huan T Joehanes R Song C Peng F Guo Y Mendelson M. Genome-wide identification of DNA methylation QTLs in whole blood highlights pathways for cardiovascular disease. Nat Commun. (2019) 10:4267. 10.1038/s41467-019-12228-z 31537805 PMC6753136

[3] Maartens G Celum C Lewin SR. HIV infection: epidemiology, pathogenesis, treatment, and prevention. Lancet. (2014) 384:258–71. 10.1016/S0140-6736(14)60164-1 24907868

[4] Dong J Chang HD Ivascu C Qian Y Rezai S Okhrimenko A. Loss of methylation at the IFNG promoter and CNS-1 is associated with the development of functional IFN-gamma memory in human CD4(+) T lymphocytes (vol 43, pg 793, 2013). Eur J Immunol. (2014) 44:3144. 10.1002/eji.201242858 23255246

[5] Minarovits J Patho-Epigenetics of Infectious Disease. Cham: Springer (2015). p. 879 10.1007/978-3-319-24738-0 26659266

[6] Zhang YF Li SK Yang KY Liu MH Lee N Tang X. Whole genome methylation array reveals the down-regulation of IGFBP6 and SATB2 by HIV-1. Sci Rep. (2015) 5:10806. 10.1038/srep10806 26039376 PMC4454074

[7] Doitsh G Galloway NL Geng X Yang Z Monroe KM Zepeda O. Corrigendum: cell death by pyroptosis drives CD4 T-cell depletion in HIV-1 infection. Nature. (2017) 544:124. 10.1038/nature22066 28329768

[8] Jin YM Ratnam K Chuang PY Fan Y Zhong YF Dai Y. A systems approach identifies HIPK2 as a key regulator of kidney fibrosis. Nat Med. (2012) 18:580–8. 10.1038/nm.2685 22406746 PMC3321097

[9] Hervouet E Cheray M Vallette FM Cartron PF. DNA methylation and apoptosis resistance in cancer cells. Cells. (2013) 2:545–73. 10.3390/cells2030545 24709797 PMC3972670

[10] Xue J Fu CY Cong Z Peng LJ Peng ZY Chen T. Galectin-3 promotes caspase-independent cell death of HIV-1-infected macrophages. Febs J. (2017) 284:97–113. 10.1111/febs.13955 27981746

[11] Pan Y Liu G Zhou F Su B Li Y DNA methylation profiles in cancer diagnosis and therapeutics. Clin Exp Med. (2018) 18:1–14. 10.1007/s10238-017-0467-0 28752221

[12] Jorgensen BG Ro S. Role of DNA methylation in the development and differentiation of intestinal epithelial cells and smooth muscle cells. J Neurogastroenterol Motil. (2019) 25:377–86. 10.5056/jnm19077 31327220 PMC6657918

[13] Wei JD Lin YL Tsai CH Shieh HS Lin PI Ho WP. SATB2 participates in regulation of menadione-induced apoptotic insults to osteoblasts. J Orthop Res. (2012) 30:1058–66. 10.1002/jor.22046 22570222

[14] Akari H Bour S Kao S Adachi A Strebel K. The human immunodeficiency virus type 1 accessory protein Vpu induces apoptosis by suppressing the nuclear factor kappa B-dependent expression of antiapoptotic factors. J Exp Med. (2001) 194:1299–311. 10.1084/jem.194.9.1299 11696595 PMC2195969

[15] Chandel N Husain M Goel H Salhan D Lan XQ Malhotra A. VDR hypermethylation and HIV-induced T cell loss. J Leukocyte Biol. (2013) 93:623–31. 10.1189/jlb.0812383 23390308 PMC3597838

[16] Zhang X Justice AC Hu Y Wang Z Zhao H Wang G. Epigenome-wide differential DNA methylation between HIV-infected and uninfected individuals. Epigenetics. (2016) 11:750–60. 10.1080/15592294.2016.1221569 27672717 PMC5094631

[17] Pion M Jaramillo-Ruiz D Martinez A Munoz-Fernandez MA Correa-Rocha R. HIV infection of human regulatory T cells downregulates Foxp3 expression by increasing DNMT3b levels and DNA methylation in the FOXP3 gene. AIDS. (2013) 27:2019–29. 10.1097/QAD.0b013e32836253fd 24201117

[18] Hamidi T Singh AK Chen T. Genetic alterations of DNA methylation machinery in human diseases. Epigenomics. (2015) 7:247–65. 10.2217/epi.14.80 25942534

[19] Langmead B Salzberg SL. Fast gapped-read alignment with Bowtie 2. Nat Methods. (2012) 9:357–9. 10.1038/nmeth.1923 22388286 PMC3322381

[20] Lienhard M Grimm C Morkel M Herwig R Chavez L. MEDIPS: genome-wide differential coverage analysis of sequencing data derived from DNA enrichment experiments. Bioinformatics. (2014) 30:284–6. 10.1093/bioinformatics/btt650 24227674 PMC3892689

[21] Trapnell C Roberts A Goff L Pertea G Kim D Kelley DR. Differential gene and transcript expression analysis of RNA-seq experiments with TopHat and Cufflinks (vol 7, pg 562, 2012). Nat Protocols. (2014) 9:2513. 10.1038/nprot1014-2513a 22383036 PMC3334321

[22] Anders S Reyes A Huber W. Detecting differential usage of exons from RNA-seq data. Genome Res. (2012) 22:2008–17. 10.1101/gr.133744.111 22722343 PMC3460195

[23] Subramanian A Tamayo P Mootha VK Mukherjee S Ebert BL Gillette MA. Gene set enrichment analysis: a knowledge-based approach for interpreting genome-wide expression profiles. Proc Natl Acad Sci USA. (2005) 102:15545–50. 10.1073/pnas.0506580102 16199517 PMC1239896

[24] Huang da W Sherman BT Lempicki RA. Systematic and integrative analysis of large gene lists using DAVID bioinformatics resources. Nat Protoc. (2009) 4:44–57. 10.1038/nprot.2008.211 19131956

[25] Kramer A Green J Pollard JJr Tugendreich S. Causal analysis approaches in ingenuity pathway analysis. Bioinformatics. (2014) 30:523–30. 10.1093/bioinformatics/btt703 24336805 PMC3928520

[26] Dao LTM Galindo-Albarran AO Castro-Mondragon JA Andrieu-Soler C Medina-Rivera A Souaid C. Genome-wide characterization of mammalian promoters with distal enhancer functions. Nat Genet. (2017) 49:1073–81. 10.1038/ng.3884 28581502

[27] Barisoni L Bruggeman LA Mundel P D'Agati VD Klotman PE. HIV-1 induces renal epithelial dedifferentiation in a transgenic model of HIV-associated nephropathy. Kidney Int. (2000) 58:173–81. 10.1046/j.1523-1755.2000.00152.x 10886562

[28] Dabrowska A Kim N Aldovini A. Tat-induced FOXO3a is a key mediator of apoptosis in HIV-1-infected human CD4+ T lymphocytes. J Immunol. (2008) 181:8460–77. 10.4049/jimmunol.181.12.8460 19050264 PMC2665797

[29] Huang Y Walstrom A Zhang L Zhao Y Cui M Ye L. Type I interferons and interferon regulatory factors regulate TNF-related apoptosis-inducing ligand (TRAIL) in HIV-1-infected macrophages. PLoS ONE. (2009) 4:e5397. 10.1371/journal.pone.0005397 19404407 PMC2672636

[30] Long E Ilie M Hofman V Havet K Selva E Butori C. LANA-1, Bcl-2, Mcl-1 and HIF-1alpha protein expression in HIV-associated Kaposi sarcoma. Virchows Arch. (2009) 455:159–70. 10.1007/s00428-009-0791-1 19484260

[31] Yndart A Kaushik A Agudelo M Raymond A Atluri VS Saxena SK. Investigation of neuropathogenesis in HIV-1 clade B and C infection associated with IL-33 and ST2 regulation. ACS Chem Neurosci. (2015) 6:1600–12. 10.1021/acschemneuro.5b00156 26110635

[32] Siangphoe U Archer KJ. Gene expression in HIV-associated neurocognitive disorders: a meta-analysis. Jaids J Acquired Immune Deficiency Syndrome. (2015) 70:479–88. 10.1097/QAI.0000000000000800 26569176

[33] Abbas AR Wolslegel K Seshasayee D Modrusan Z Clark HF. Deconvolution of blood microarray data identifies cellular activation patterns in systemic lupus erythematosus. PLoS ONE. (2009) 4:e6098. 10.1371/journal.pone.0006098 19568420 PMC2699551

[34] Selliah N Finkel TH. Biochemical mechanisms of HIV induced T cell apoptosis. Cell Death Differentiation. (2001) 8:127–36. 10.1038/sj.cdd.4400822 11313714

[35] Cummins NW Badley AD. Mechanisms of HIV-associated lymphocyte apoptosis: 2010. Cell Death Dis. (2010) 1:77. 10.1038/cddis.2010.77 21368875 PMC3032328

[36] Pasquereau S Kumar A Herbein G. Targeting TNF and TNF receptor pathway in HIV-1 infection: from immune activation to viral reservoirs. Viruses. (2017) 9:40064. 10.3390/v9040064 28358311 PMC5408670

[37] Liu S Guan W. STING signaling promotes apoptosis, necrosis, and cell death: an overview and update. Mediators Inflamm. (2018) 2018:1202797. 10.1155/2018/1202797 30595664 PMC6286756

[38] Bird AP Wolffe AP. Methylation-induced repression—belts, braces, and chromatin. Cell. (1999) 99:451–4. 10.1016/S0092-8674(00)81532-9 10589672

[39] Wade PA. Methyl CpG-binding transcriptional proteins and repression. Bioessays. (2001) 23:1131–7. 10.1002/bies.10008 11746232

[40] Smith ZD Meissner A DNA methylation: roles in mammalian development. Nat Rev Genetics. (2013) 14:204–20. 10.1038/nrg3354 23400093

[41] Scharer CD Barwick BG Youngblood BA Ahmed R Boss JM. Global DNA methylation remodeling accompanies CD8 T cell effector function. J Immunol. (2013) 191:3419–29. 10.4049/jimmunol.1301395 23956425 PMC3800465

[42] Corley MJ Dye C D'Antoni ML Byron MM Yo KLA Lum-Jones A. Comparative DNA methylation profiling reveals an immunoepigenetic signature of HIV-related cognitive impairment. Sci Rep. (2016) 6:33310. 10.1038/srep33310 27629381 PMC5024304

[43] Jones PA. Functions of DNA methylation: islands, start sites, gene bodies and beyond. Nat Rev Genetics. (2012) 13:484–92. 10.1038/nrg3230 22641018

[44] Maunakea AK Chepelev I Cui KR Zhao KJ. Intragenic DNA methylation modulates alternative splicing by recruiting MeCP2 to promote exon recognition. Cell Res. (2013) 23:1256–69. 10.1038/cr.2013.110 23938295 PMC3817542

[45] Theron AJ Anderson R Rossouw TM Steel HC. The role of transforming growth factor beta-1 in the progression of HIV/AIDS and development of non-AIDS-defining fibrotic disorders. Front Immunol. (2017) 8:1461. 10.3389/fimmu.2017.01461 29163528 PMC5673850

[46] Coyle-Rink J Sweet T Abraham S Sawaya B Batuman O Khalili K. Interaction between TGFbeta signaling proteins and C/EBP controls basal and Tat-mediated transcription of HIV-1 LTR in astrocytes. Virology. (2002) 299:240–7. 10.1006/viro.2002.1439 12202226

[47] Eldeen MB Deshmane SL Simbiri K Khalili K Amini S Sawaya BE. MH2 domain of Smad3 reduces HIV-1 Tat-induction of cytokine secretion. J Neuroimmunol. (2006) 176:174–80. 10.1016/j.jneuroim.2006.04.004 16750572

[48] Bachelerie F Alcami J Arenzanaseisdedos F Virelizier JL. Hiv enhancer activity perpetuated by Nf-Kappa-B induction on infection of monocytes. Nature. (1991) 350:709–12. 10.1038/350709a0 2023633

[49] Cunliffe VT. Histone modifications in zebrafish development. Zebrafish. (2016) 135:361–85. 10.1016/bs.mcb.2016.05.005 27443936

[50] Zheng M Lin FL Hou FX Li GL Zhu CY Xu PY Association between promoter methylation of gene ERCC3 and benzene hematotoxicity. Int J Environ Res Public Health. (2017) 14:4080921 10.3390/ijerph14080921 PMC5580623 28813025

[51] de la Rica L Rodriguez-Ubreva J Garcia M Islam ABMMK Urquiza JM Hernando H. PU.1 target genes undergo Tet2-coupled demethylation and DNMT3b-mediated methylation in monocyte-to-osteoclast differentiation. Genome Biol. (2013) 14:r99. 10.1186/gb-2013-14-9-r99 24028770 PMC4054781

[52] Schachter J Delgado KV Barreto-de-Souza V Bou-Habib DC Persechini PM Meyer-Fernandes JR. Inhibition of ecto-ATPase activities impairs HIV-1 infection of macrophages. Immunobiology. (2015) 220:589–96. 10.1016/j.imbio.2014.12.004 25577295

[53] Anderson JL Hope TJ. HIV accessory proteins and surviving the host cell. Curr HIV/AIDS Rep. (2004) 1:47–53. 10.1007/s11904-004-0007-x 16091223

[54] Tokarev A Guatelli J. Misdirection of membrane trafficking by HIV-1 Vpu and Nef: keys to viral virulence and persistence. Cell Logist. (2011) 1:90–102. 10.4161/cl.1.3.16708 21922073 PMC3173656

[55] Aissani B Boehme AK Wiener HW Shrestha S Jacobson LP Kaslow RA. SNP screening of central MHC-identified HLA-DMB as a candidate susceptibility gene for HIV-related Kaposi's sarcoma. Genes Immunity. (2014) 15:424–9. 10.1038/gene.2014.42 25008864 PMC4174341

[56] Barnitz RA Wan F Tripuraneni V Bolton DL Lenardo MJ. Protein kinase A phosphorylation activates Vpr-induced cell cycle arrest during human immunodeficiency virus type 1 infection. J Virol. (2010) 84:6410–24. 10.1128/JVI.02273-09 20392842 PMC2903295

[57] Verhofstede C Nijhuis M Vandekerckhove L. Correlation of coreceptor usage and disease progression. Curr Opin HIV AIDS. (2012) 7:432–9. 10.1097/COH.0b013e328356f6f2 22871636

