# Supplementary material for: Genome-Wide Characterization of Host Transcriptional and Epigenetic Alterations During HIV Infection of T Lymphocytes
Source: Front Immunol. 2020 Sep 10;11:2131. doi: 10.3389/fimmu.2020.02131 (PMC7511662; doi:10.3389/fimmu.2020.02131)
Supplement: Supplementary file 1 [file Data_Sheet_1.PDF]

Table of Contents

Fig S1. Distribution of hyper- and hypomethylated DMRs in HIV+ samples...................... 2

Fig S2. DMRs were enriched for transcription-factor-binding sites..................................... 6

Fig S3. Overlap of DMGs between the MT-2 and Jurkat cell lines...................................... 7

Fig S4. Representative IGV results regarding methylation, together with validation by bisulfite sequencing.. 9

Fig S5. Enriched canonical pathways of the DEGs of the MT-2 and Jurkat cell lines. 14

Fig S6. Alternative splicing of LGALS1 in the Jurkat cell line.. 15

Fig S7. Expression of selected genes, as quantified by RT–qPCR in HIV+ Jurkat cells relative to the uninfected control... 17

Fig S8. Network of the upstream regulator TGFB1 in MT-2 cells..................................... 19

Fig S9. Network of the upstream regulator TGFB1 in Jurkat cells..................................... 20

Fig S10. Effect of siRNA knockdown on cell viability and apoptosis. 21

Fig S11. Western blot analysis after the knockdown of five target genes. 22

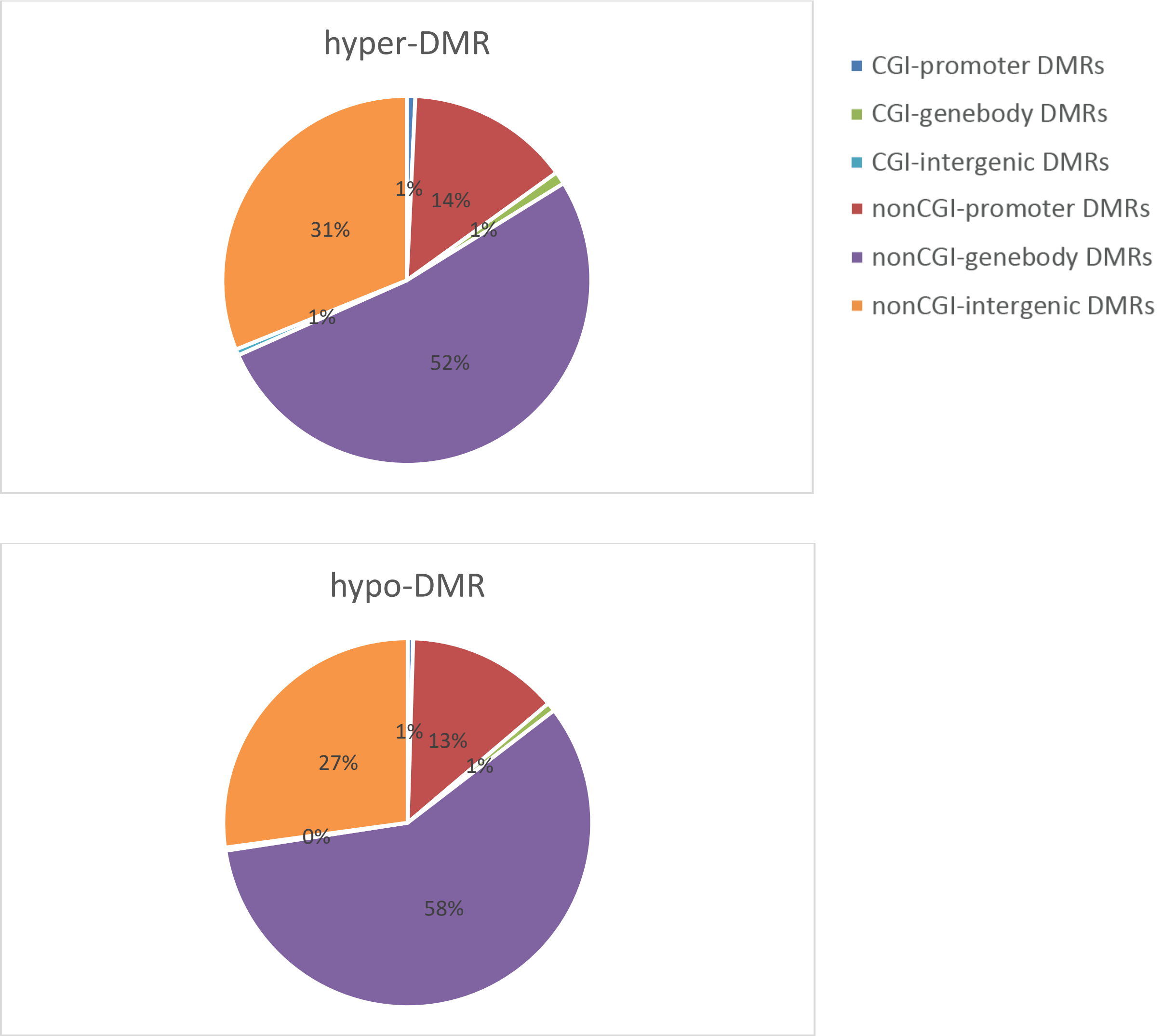

Fig S1. Distribution of hyper- and hypomethylated DMRs in HIV+ samples.

A

Motif	TF	Adjusted p-value	Functions
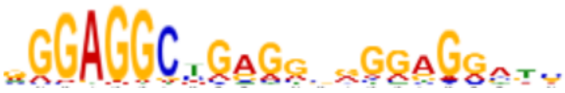	ZNF770	2.64E-19	Zinc Finger Protein 770, nucleic acid binding and RNA polymerase II transcription factor activity,
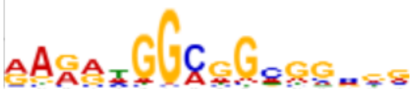	TAF1	1.24E-15	TATA-box binding protein associated factor 1,protein polyubiquitination,RNA polymerase II core promoter
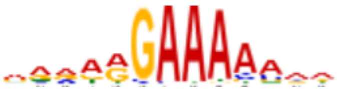	PRDM6	7.48E-15	PR/SET domain 6,nucleic acid bindingtranscription, DNA-templated
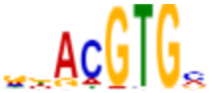	HIF1A	1.48E-14	hypoxia inducible factor 1 alpha subunit, angiogenesis,nucleoplasm,transcription factor activity
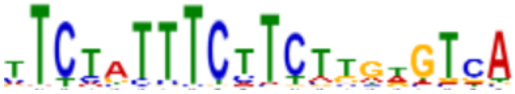	ZFP28	6.68E-14	transcription,nucleic acid binding
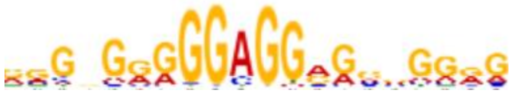	WT1	2.35E-13	Wilms tumor 1,negative regulation of transcription from RNA polymerase II promoter
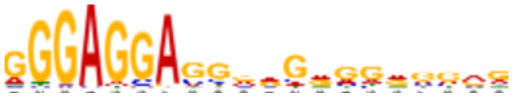	MAZ	8.38E-12	MYC associated zinc finger protein,regulation of transcription
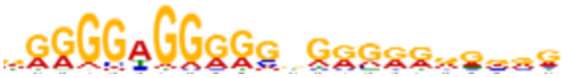	ZN263	8.72E-12	Zinc Finger Protein 263, nucleic acid binding and transcription factor activity
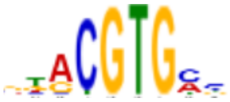	ARNT	8.34E-11	aryl hydrocarbon receptor nuclear translocator,response to hypoxia,DNA binding
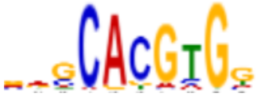	MAX	8.89E-11	MYC Associated Factor X,ranscription factor activity, sequence-specific DNA binding

B

Motif	TF	Adjusted p-value	Functions
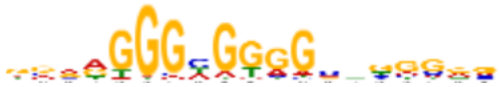	KLF6	2.18E-14	Kruppel like factor 6, transcription, B cell differentiation, Prostate cancer, somatic, nucleic acid binding
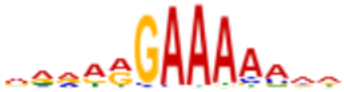	PRDM6	3.28E-13	PR/SET domain 6, transcription, nucleic acid binding,
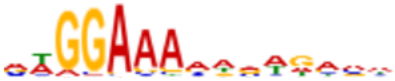	NFAC1	7.65E-12	Nuclear Factor Of Activated T-Cells 1, integral for the development and function of the immune system
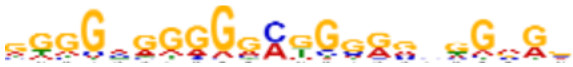	PATZ1	6.82E-10	POZ/BTB and AT hook containing zinc finger 1, transcription, T cell differentiation
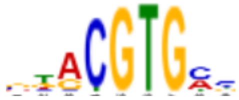	ARNT	1.19E-09	aryl hydrocarbon receptor nuclear translocator, response to hypoxia, DNA binding, transcription factor activity
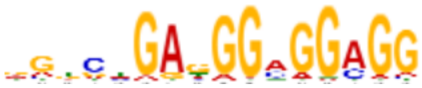	ZSC22	2.29E-09	Zinc Finger And SCAN Domain Containing 22, transcription factor activity, sequence-specific DNA binding
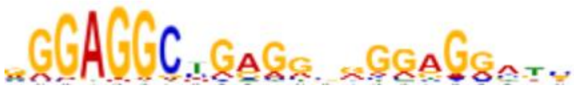	ZNF770	7.30E-09	Zinc Finger Protein 770, nucleic acid binding and RNA polymerase II transcription factor activity
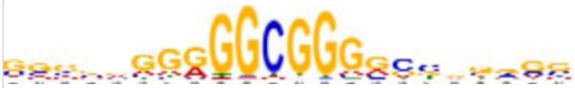	SP2	1.57E-08	Sp2 transcription, transcription,RNA polymerase II transcription factor activity
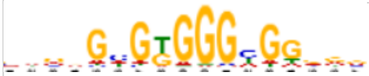	EGR1	1.58E-08	early growth response,negative regulation of transcription from RNA polymerase II promoter
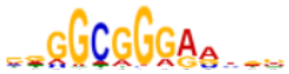	E2F7	2.77E-08	E2F transcription factor 7, negative regulation of transcription from RNA polymerase II promoter

C

Motif	TF	Adjusted p-value	Functions
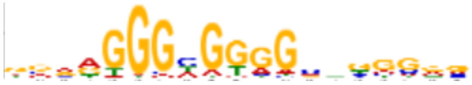	KLF6	6.13E-11	Kruppel like factor 6, transcription, B cell differentiation, Prostate cancer, somatic, nucleic acid binding
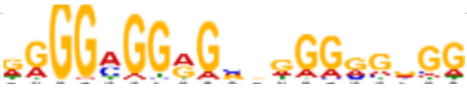	KLF15	3.81E-10	Kruppel Like Factor 15, ,transcriptional activator activity
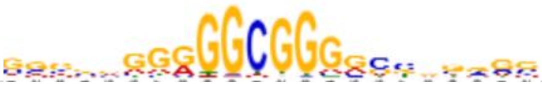	SP2	5.00E-09	Sp2 transcription factor, transcription,RNA polymerase II transcription factor activity
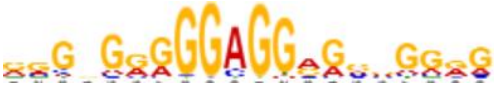	WT1	2.84E-08	Wilms Tumor 1, negative regulation of transcription from RNA polymerase II promoter,transcriptional activator
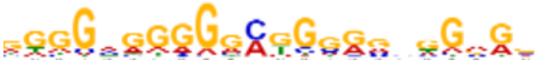	PATZ1	1.04E-07	POZ/BTB and AT hook containing zinc finger , transcription, T cell differentiation
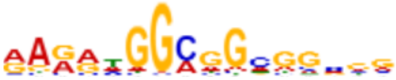	TAF1	5.93E-07	TATA-box binding protein associated factor 1,protein polyubiquitination
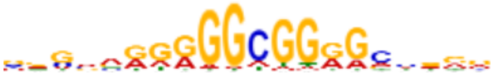	SP4	7.25E-07	Sp4 transcription factor, transcription
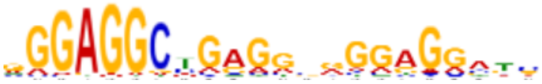	ZNF770	1.23E-06	Zinc Finger Protein 770, nucleic acid binding and RNA polymerase II transcription factor activity
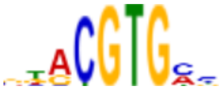	ARNT	1.95E-06	aryl hydrocarbon receptor nuclear translocator, response to hypoxia, DNA binding, transcription factor activity
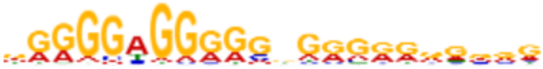	MAZ	3.04E-06	MYC associated zinc finger protein,regulation of transcription

D

Motif	TF	Adjusted p-value	Functions
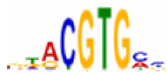	ARNT	1.51E-26	aryl hydrocarbon receptor nuclear translocator, response to hypoxia,transcription factor activity
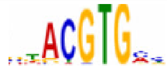	EPAS1	3.46E-22	endothelial PAS domain protein 1, angiogenesis,nucleus,~transcriptional activator activity
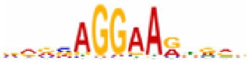	ETV5	4.76E-22	ETS Variant 5, transcription factor activity, associated with ETV5 include Sertoli Cell-Only Syndrome
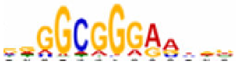	E2F7	7.26E-21	negative regulation of transcription from RNA polymerase II promoter
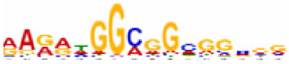	TAF1	1.02E-21	TATA-box binding protein associated factor ,transcription factor activity
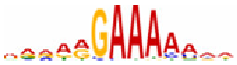	PRDM6	1.30E-20	PR/SET domain 6, transcription,nucleic acid binding
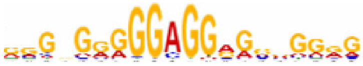	WT1	1.46E-20	Wilms Tumor 1, negative regulation of transcription from RNA polymerase II promoter
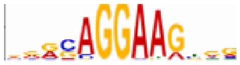	ERG	3.82E-19	early growth response,transcription regulatory region sequence-specific DNA binding
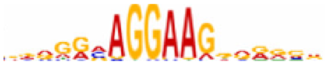	FLI1	9.07E-19	Fli-1 Proto-Oncogene, Transcriptional misregulation in cancer and NF-kappaB Signaling.
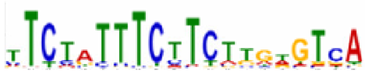	ZFP28	1.46E-18	ZFP28 zinc finger protein, transcription, DNA-templated,nucleic acid binding

Fig S2. DMRs were enriched for transcription-factor-binding sites.

Transcription factor binding motifs significantly enriched in: (a) hypermethylated DMRs for MT-2 cell line; (b) hypomethylated DMRs for MT-2 cell line; (c) hypermethylated DMRs for Jurkat cell line; (d) hypomethylated DMRs for Jurkat cell line.

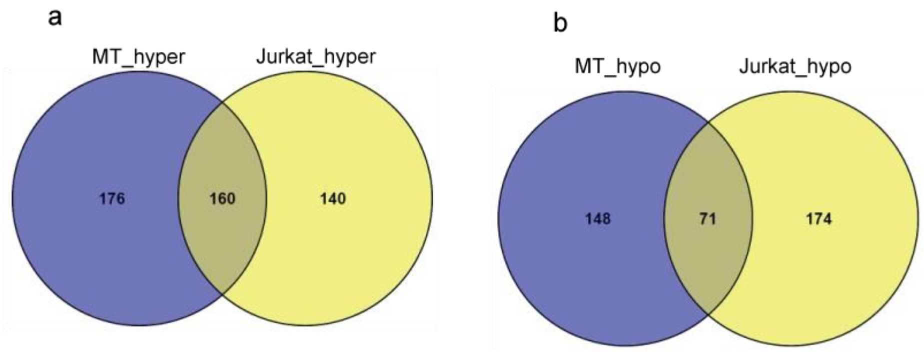

Fig S3. Overlap of DMGs between the MT-2 and Jurkat cell lines.

(a) The number of overlapping genes with hyper-methylated DMRs in gene bodies or promoters between MT-2 and Jurkat cell lines; (b) The number of overlapping genes with hyper-methylated DMRs in gene bodies or promoters between MT-2 and Jurkat cell lines.

A

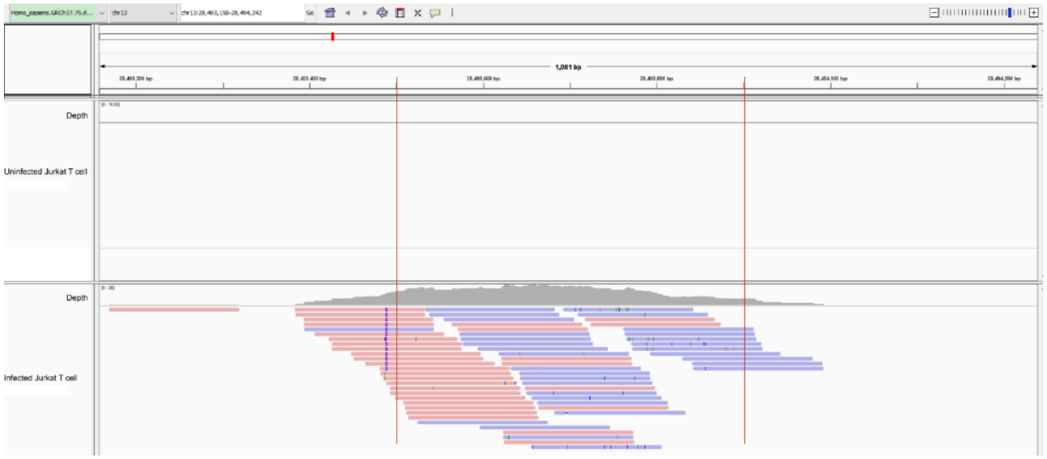

B

HIV infected Jurkat T cells

```
1   AAGAATTTAAAAAAGTTTTTGTGAATGTTTTAGAAGTTATCGTTTATATTTTAGAAGTATTTGTAGTATATTTATAAGTAAAAATATATAACGAATGTTAGAGTTTCGTGTGTTTTTTAA
2   AAGAATTTAAAAAAGTTTTTGTGAATGTTTTAGAAGTTATCGTTTATATTTTAGAAGTATTTGTAGTATATTTATAAGTAAAAATATATAACGAATGTTAGAGTTTCGTGTGTTTTTTAA
3   AAGAATTTAAAAAAGTTTTTGTGAATGTTTTAGAAGTTATCGTTTATATTTTAGAAGTATTTGTAGTATATTTATAAGTAAAAATATATAACGAATGTTAGAGTTTTGTGTGTTTTTTAA
4   AAGAATTTAAAAAAGTTTTTGTGAATGTTTTAGAAGTTATCGTTTATATTTTAGAAGTATTTGTAGTATATTTATAAGTAAAAATATATAACGAATGTTAGAGTTTCGTGTGTTTTTTAA
5   AAGAATTTAAAAAAGTTTTTGTGAATGTTTTAGAAGTTATCGTTTATATTTTAGAAGTATTTGTAGTATATTTATAAGTAAAAATATATAACGAATGTTAGAGTTTCGTGTGTTTTTTAA
6   AAGAATTTAAAAAAGTTTTTGTGAATGTTTTAGAAGTTATCGTTTATATTTTAGAAGTATTTGTAGTATATTTGTAAGTAAAAATATATAACGAATGTTAGAGTTTCGTGTGTTTTTTAA
7   AAGAATTTAAAAAAGTTTTTGTGAATGTTTTAGAAGTTATCGTTTATATTTTAGAAGTATTTGTAGTATATTTATAAGTAAAAATATATAACGAATGTTAGAGTTTCGTGTGTTTTTTAA
8   AAGAATTTAAAAAAGTTTTTGTGAATGTTTTAGAAGTTATTGTTTATATTTTAGAAGTATTTGTAGTGTATTTATAAGTAAAAATATATAATGAATGTTAGAGTTTTGTGTGTTCTTTAA
9   AAGAATTTAAAAAAGTTTTTGTGAATGTTTTAGAAGTTATCGTTTATATTTTAGAAGTATTTGTAGTATATTTATAAGTAAAAATATATAACGAATGTTAGAGTTTCGTGTGTTTTTTAA
10  AAGAATTTAAAAAAGTTTTTGTGAATGTTTTAGAAGTTATCGTTTATATTTTAGAAGTATTTGTAGTATATTTATAAGTAAAAATATATAACGAATGTTAGAGTTTCGTGTGTTTTTTAA
```

```
1   TCGATATTTTTGTGGCTGTGAATAAATTTTATAAATAAAATAGAATTAAATGTTTTTGATTTAGAGAGTTGGGTTTGTAAATTTTTTTTTTTATTGTA
2   TCGATATTTTTGTGGTTGTGAATAAATTTTATAAATAAAATAGAATTAAATGTTTTTGATTTAGAGAGTTGGGTTTGTAAATTTTTTTTTTTATCGTA
3   TCGATATTTTTGTGGTTGTGAATAAATTTTATAAATAAAATAGAATTAAGTGTTTTTGATTTAGAGAGTTGGGTTTGTAAATTTTTTTTTTTATCGTA
4   TCGATATTTTTGTGGTTGTGAATAAATTTTATAAATAAAATAGAATTAAATGTTTTTGATTTAGAGAGTTGGGTTTGTAAATTTTTTTTTTTATTGTA
5   TCGATATTTTTGTGGTTGTGAATAAATTTTATAAATAAAATAGAATTAAATGTTTTTGATTTAGAGAGTTGGGTTTGTAAATTTTTTTTTTTATCGTA
6   TCGATATTTTTGTGGTTGTGAATAAATTTTATAAATAAAATAGAATTAAATGTTTTTGATTTAGAGAGTTGGGTTTGTAAATTTTTTTTTTTATCGTA
```

```
7   TCGATATTTTTGTGGTTGTGAATAAATTTTATAAATAAAATAGAATTAAATGTTTTTGATTTAGAGAGTTGGGTTTGTAAATTTTTTTTTTTATCGTA
8   TCGATATTTTTGTGGTTGTGAATAAATTTTATAAATAAAATAGAATTAAATGTTTTTGATTTAGAGAGTTGGGTTTGTAAATTTTTTTTTTTATCGTA
9   TCGATATTTTTGTGGCTGTGAATAAATTTTATAAATAAAATAGAATTAAATGTTTTTGATTTAGAGAGTTGGGTTTGTAAATTTTTTTTTTTATCGTA
10  TCGATATTTTTGTGGTTGTGAATAAATTTTATAAATAAAATAGAATTAAATGTTTTTGATTTAGAGAGTTGGGTTTGTAAATTTTTTTTTTTATCGTA
```

Control (Uninfected Jurkat T cells)

```
1   AAGAATTTAAAAAAGTTTTTGTGAATGTTTTAGAAGTTATTGTTTATATTTTAGAAGTATTTGTAGTATATTTATAAGTAAAAATATATAATGAATGTTAGAGTTTTGTGTGTTTTTTAA
2   AAGAATTTAAAAAAGTTTTTGTGAATGTTTTAGAAGTTATTGTTTATATTTTAGAAGTATTTGTAGTATATTTATAAGTAAAAATATATAATGAATGTTAGAGTTTTGTGTGTTTTTTAA
3   AAGAATTTAAAAAAGTTTTTGTGAATGTTTTAGAAGTTATTGTTTATATTTTAGAAGTATTTGTAGTATATTTATAAGTAAAAATATATAATGAATGTTAGAGTTTTGTGTGTTTTTTAA
4   AAGAATTTAAAAAAGTTTTTGTGAATGTTTTAGAAGTTATTGTTTATATTTTAGAAGTATTTGTAGTATATTTATAAGTAAAAATATATAACGAATGTTAGAGTTTTGTGTGTTTCTTAA
5   AAGAATTTAAAAAAGTTTTTGTGAATGTTTTAGAAGTTATTGTTTATATTTTAGAAGTATTTGTAGTATATTTATAAGTAAAAATATATAATGAATGTTAGAGTTTTGTGTGTTTTTTAA
6   AAGAATTTAAAAAAGTTTTTGTGAATGTTTTAGAAGTTATTGTTTATATTTTAGAAGTATTTGTAGTATATTTATAAGTAAAAGTATATAATGAATGTTAGAGTTTTGTGTGTTTTTTAA
7   AAGAATTTAAAAAAGTTTTTGTGAATGTTTTAGAAGTTATTGTTTATATTTTAGAAGTATTTGTAGTATATTTATAAGTAAAAATATATAATGAATGTTAGAGTTTCGTGTGTTTTTTAA
8   AAGAATTTAAAAAAGTTTTTGTGAATGTTTTAGAAGTTATTGTTTATATCTTAGAAGTATTTGTAGTATATTTATAAGTAAAAATATATAATGAATGTTAGAGTTTTGTGTGTTTTTTAA
9   AAGAATTTAAAAAAGTTTTTGTGAATGTTTTAGAAGTTATTGTTTATATTTTAGAAGTATTTGTAGTATATTTATAAGTAAAAATATATAATGAATGTTAGAGTTTTGTGTGTTTTTTAA

1   TTGATATTTTTGTGGTTGTGAATAAATTTTATAAATAAAATAGAATTAAATGTTTTTGATTTAGAGAGTTGGGTTTGTAAATTTTTTTTTTTATCGTA
2   TTGATATTTTTGTGGTTGTGAATAAATTTTATAAATAAAATAGAATTAAATGTTTTTGATTTAGAGAGTTGGGTTTGTAAATTTTTTTTTTTATTGTA
3   TTGATATTTTTGTGGTTGTGAATAAATTTCATAAATAAAATAGAATTAAATGTTTTTGATTTAGAGAGTTGGGTTTGTAAATTTTTTTTTTTATCGTA
4   TTGATATTTTTGTGGTTGTGAATAAATTTTATAAATAAAATAGTATTAAATGTTTTTGATTTAGAGAGTTGGGTTTGTAAATTTTTTTTTTTATTGTA
5   TTGATATTTTTGTGGTTGTGAATAAATTTTATAAATAAAATAGAATTAAATGTTTTTGATTTAGAGAGTTGGGTTTGTAAATTTTTTTTTTTATTGTA
6   TTGATATTTTTGTGGTTGTGAATAAATTTTATAAATAAAATAGAATTAAATGTTTTTGATTTAGAGAGTTGGGTTTGTAAATTTTTTTTTTTATCGTA
7   TTGATATTTTTGTGGTTGTGAATAAATTTTATAAATAAAATAGAATTAAATGTTTTTGATTTAGAGAGTTGGGTTTGTAAATTTTTTTTTTTATCGTA
8   TTGATATTTTTGTGGTTGTGAATAAATTTTATAAATAAAATAGAATTAAATGTTTTTGATTTAGAGAGTTGGGTTTGTAAATTTTTTTTTTTATCGTA
9   TTGATATTTTTGTGGTTGTGAATAAATTTTATAAATAAAATAGAATTAAATGTTTTTGATTTAGAGAGTTGGGTTTGTAAATTTTTTTTTTTATTGTA
```

Fig S4. Representative IGV results regarding methylation, together with validation by bisulfite sequencing.

(A) The IGV screen for the differentially methylated region (DMR) hyper-methylated in HIV-infected human Jurkat T cells on the promoter of PDX1. The region between the two red vertical lines is DMR. **(B)** The validation results of DMR hyper-methylated in HIV infected human Jurkat T cells on the promoter of PDX1 by bisulfite sequencing. Methylated cytosines are highlighted in red and nine clones were analyzed for this region.

A. Upregulated genes of MT-2 cell

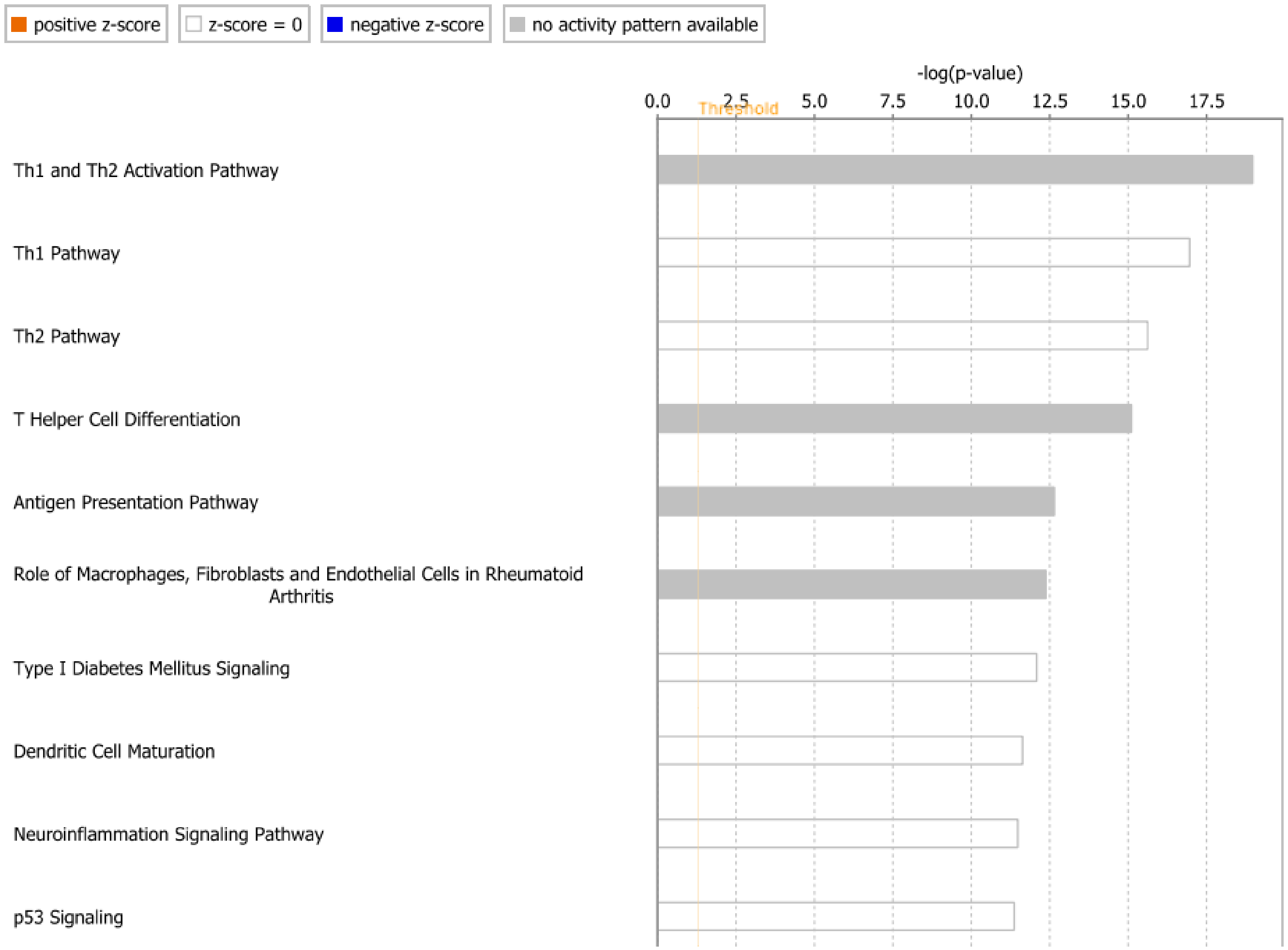

B. Downregulated genes of MT-2 cell

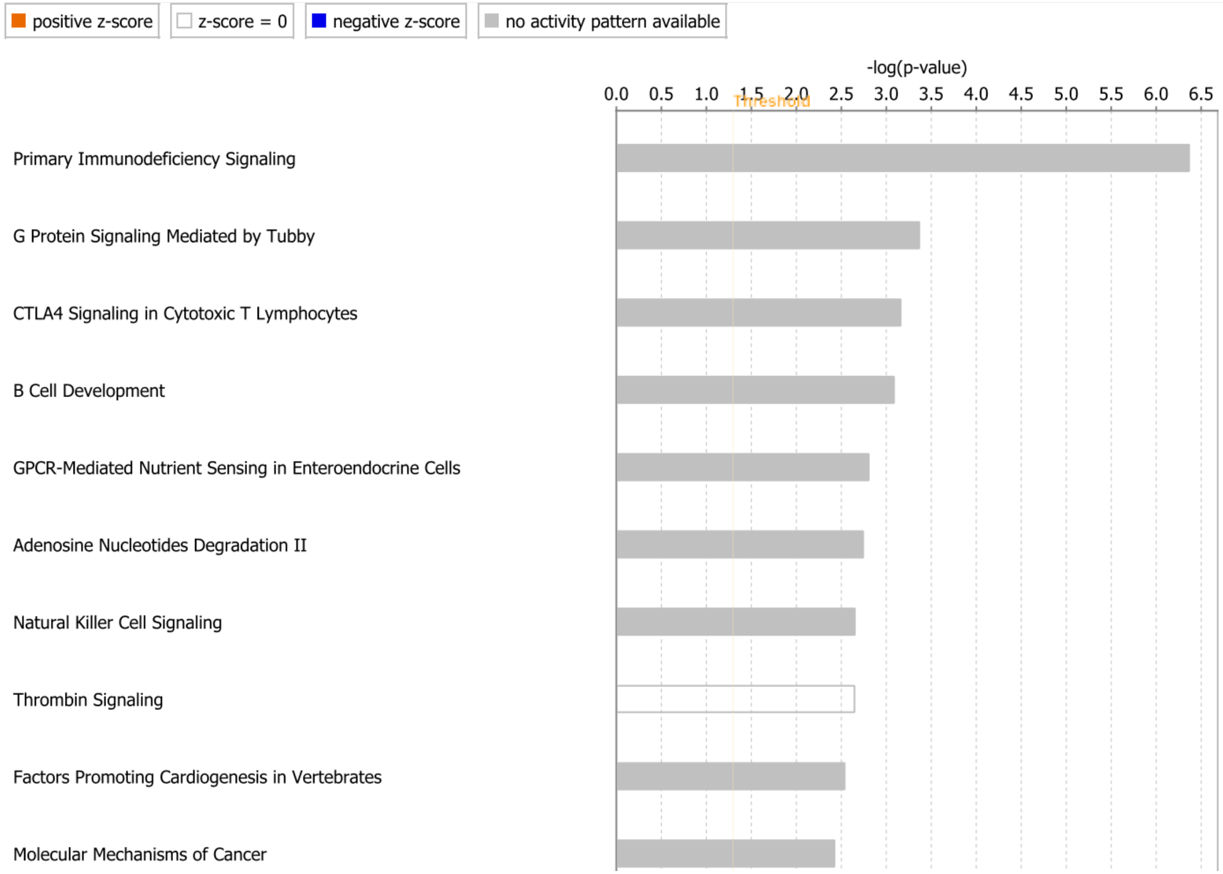

C. Upregulated genes of Jurkat cell

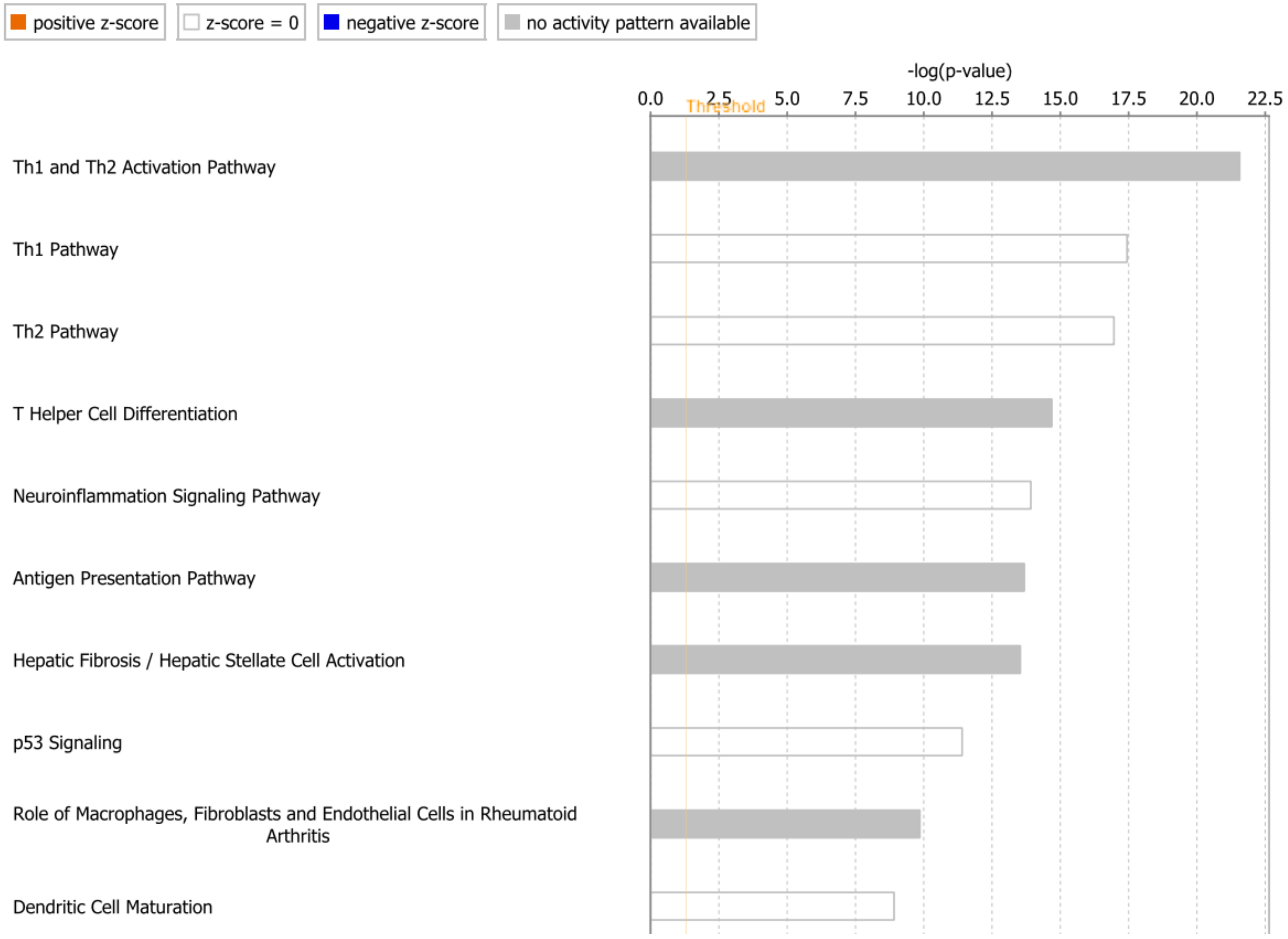

D. downregulated genes of Jurkat cell

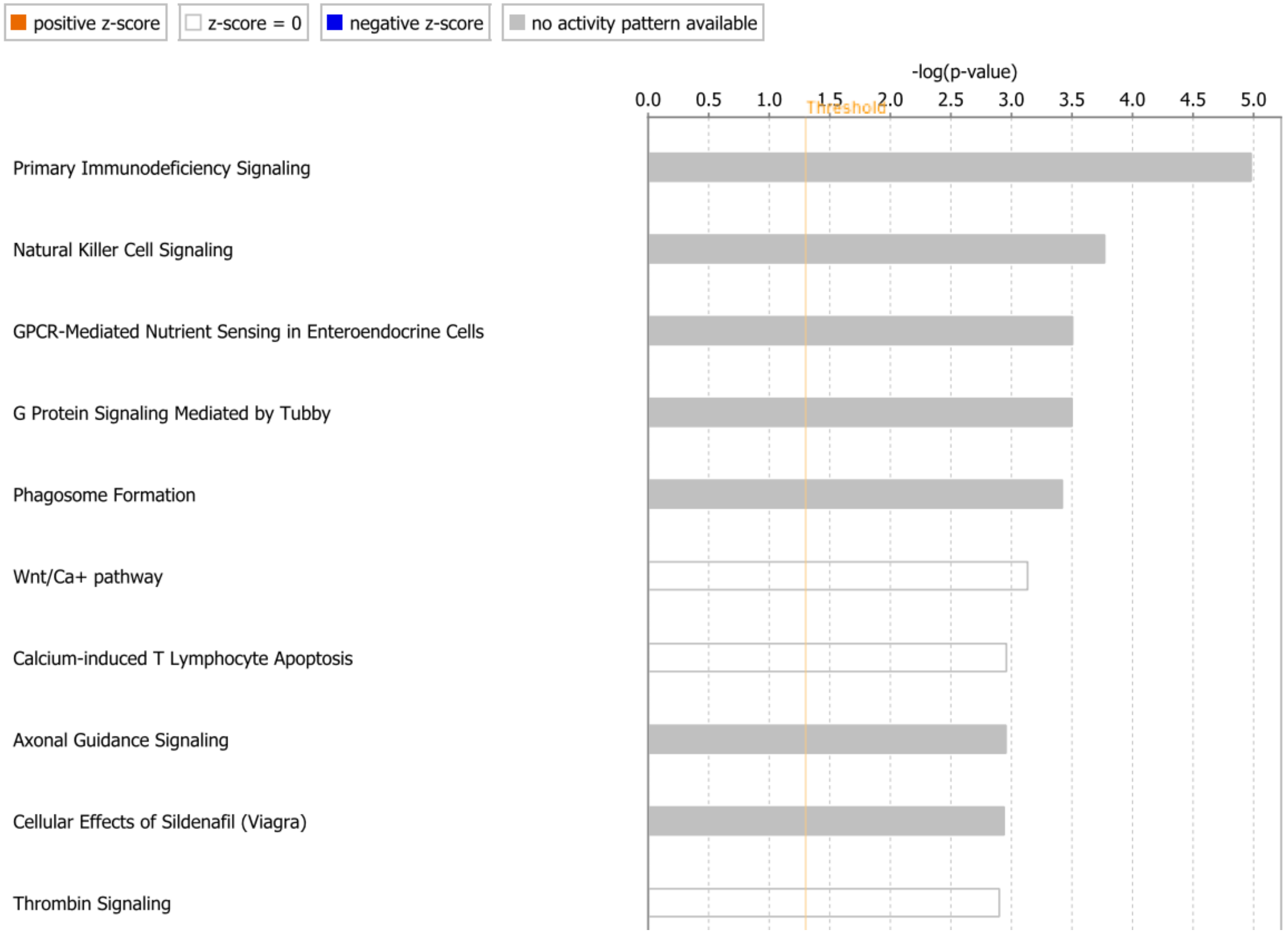

Fig S5. Enriched canonical pathways of the DEGs of the MT-2 and Jurkat cell lines.

(A) Upregulated genes of MT-2 cell; (B) Downregulated genes of MT-2 cell ; (C) Upregulated genes of Jurkat cell; (D) Downregulated genes of Jurkat cell

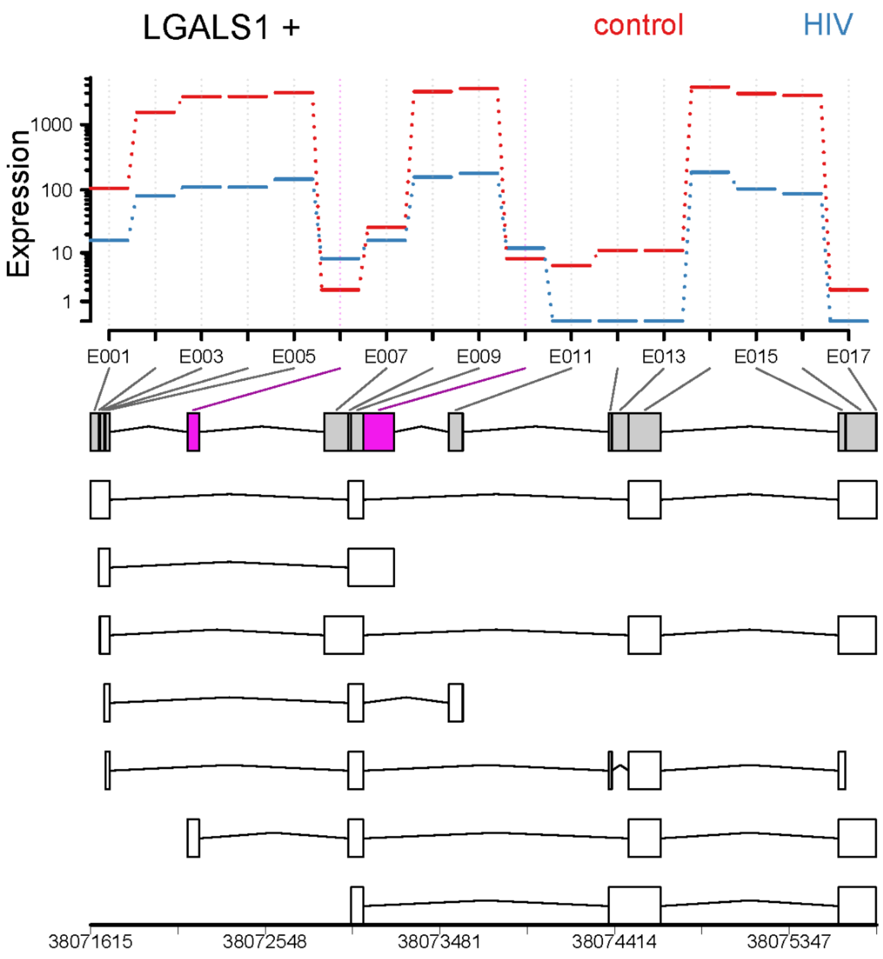

Fig S6. Alternative splicing of LGALS1 in the Jurkat cell line.

The exon in pink color were alternatively spliced between HIV-1 infected and uninfected cell.

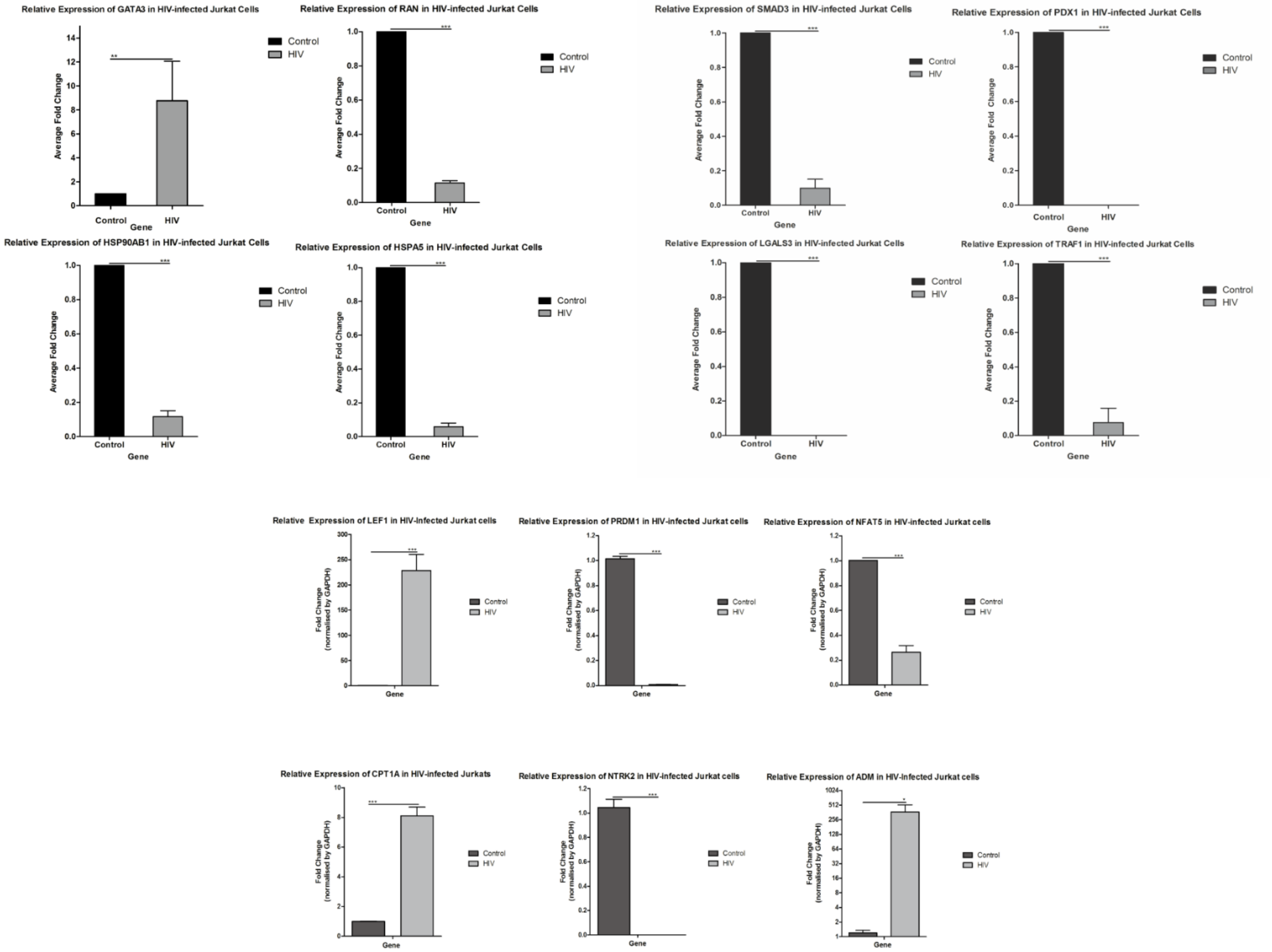

Relative Expression of GATA3 in HIV-infected Jurkat Cells
Average Fold Change
Control
HIV
Gene
**
Relative Expression of RAN in HIV-infected Jurkat Cells
***
Relative Expression of SMAD3 in HIV-infected Jurkat Cells
***
Relative Expression of PDX1 in HIV-infected Jurkat Cells
***
Relative Expression of HSP90AB1 in HIV-infected Jurkat Cells
***
Relative Expression of HSPA5 in HIV-infected Jurkat Cells
***
Relative Expression of LGALS3 in HIV-infected Jurkat Cells
***
Relative Expression of TRAF1 in HIV-infected Jurkat Cells
***
Relative Expression of LEF1 in HIV-Infected Jurkat cells
Fold Change (normalised by GAPDH)
***
Relative Expression of PRDM1 in HIV-infected Jurkat cells
***
Relative Expression of NFAT5 in HIV-infected Jurkat cells
***
Relative Expression of CPT1A in HIV-infected Jurkats
***
Relative Expression of NTRK2 in HIV-infected Jurkat cells
***
Relative Expression of ADM in HIV-infected Jurkat cells
*

Fig S7. Expression of selected genes, as quantified by RT–qPCR in HIV+ Jurkat cells relative to the uninfected control.

GATA3: GATA binding protein 3; *RAN*: Ras-related Nuclear Protein; *HSP90AB1*: Heat Shock Protein 90 Alpha Family Class B Member 1; *HSPA5*: Heat Shock Protein Family A; *SMAD3*: SMAD family member 3; *PDX1*: Pancreatic and Duodenal Homeobox 1; *LGALS3*: Galectin 3; *TRAF1*: TNF Receptor Associated Factor 1. Statistical significance ***: $p \leq 0.001$, **: $p \leq 0.01$, *: $p \leq 0.05$.

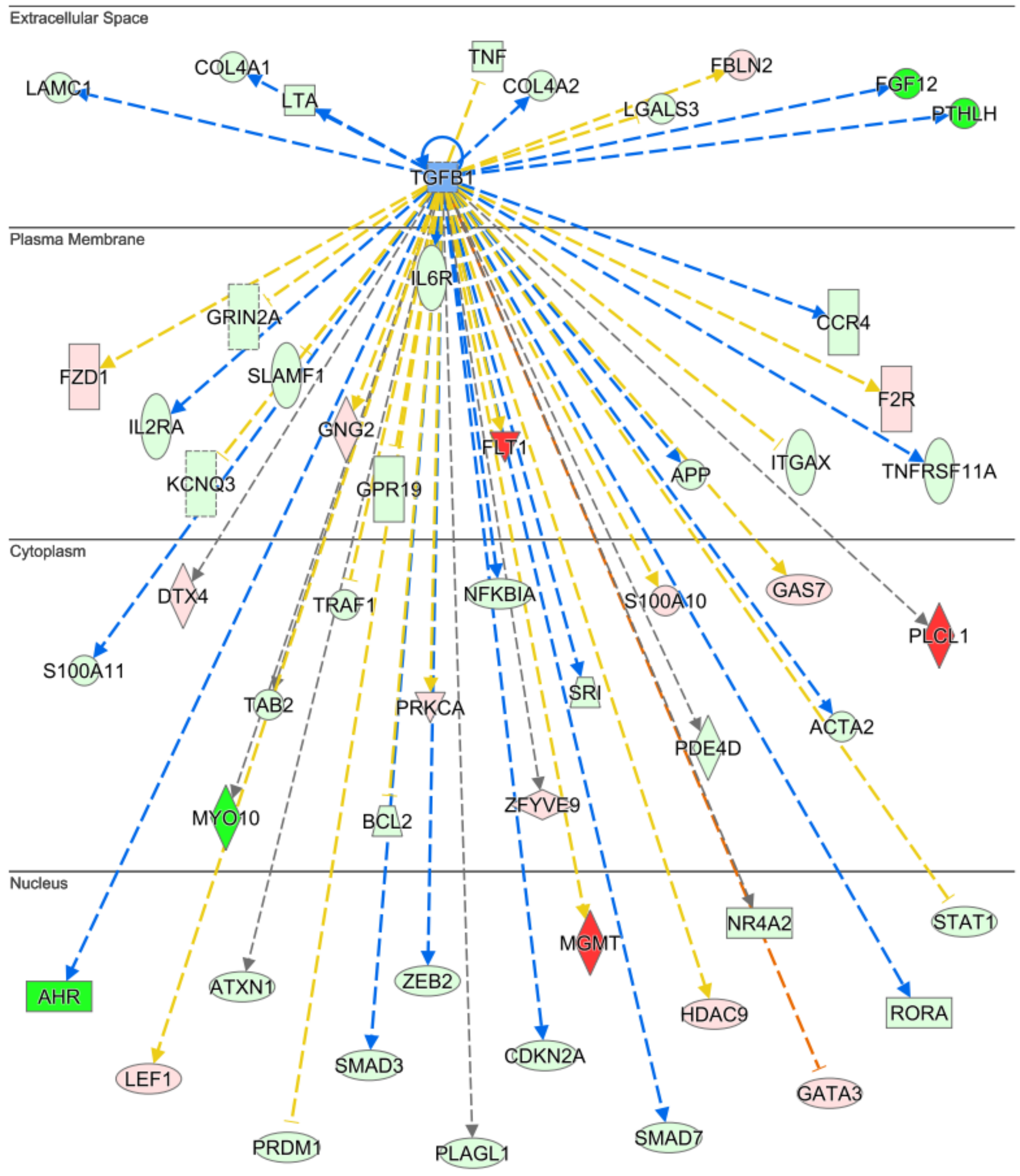
Extracellular Space
LAMC1
COL4A1
LTA
TNF
COL4A2
FBLN2
LGALS3
FGF12
PTHLH
TGFB1
Plasma Membrane
IL6R
GRIN2A
CCR4
FZD1
SLAMF1
F2R
IL2RA
GNG2
FLT1
KCNQ3
GPR19
APP
ITGAX
TNFRSF11A
Cytoplasm
DTX4
TRAF1
NFKBIA
S100A10
GAS7
PLCL1
S100A11
TAB2
PRKCA
SRI
PDE4D
ACTA2
MYO10
BCL2
ZFYVE9
Nucleus
NR4A2
STAT1
MGMT
AHR
ATXN1
ZEB2
HDAC9
RORA
LEF1
SMAD3
CDKN2A
GATA3
PRDM1
PLAGL1
SMAD7

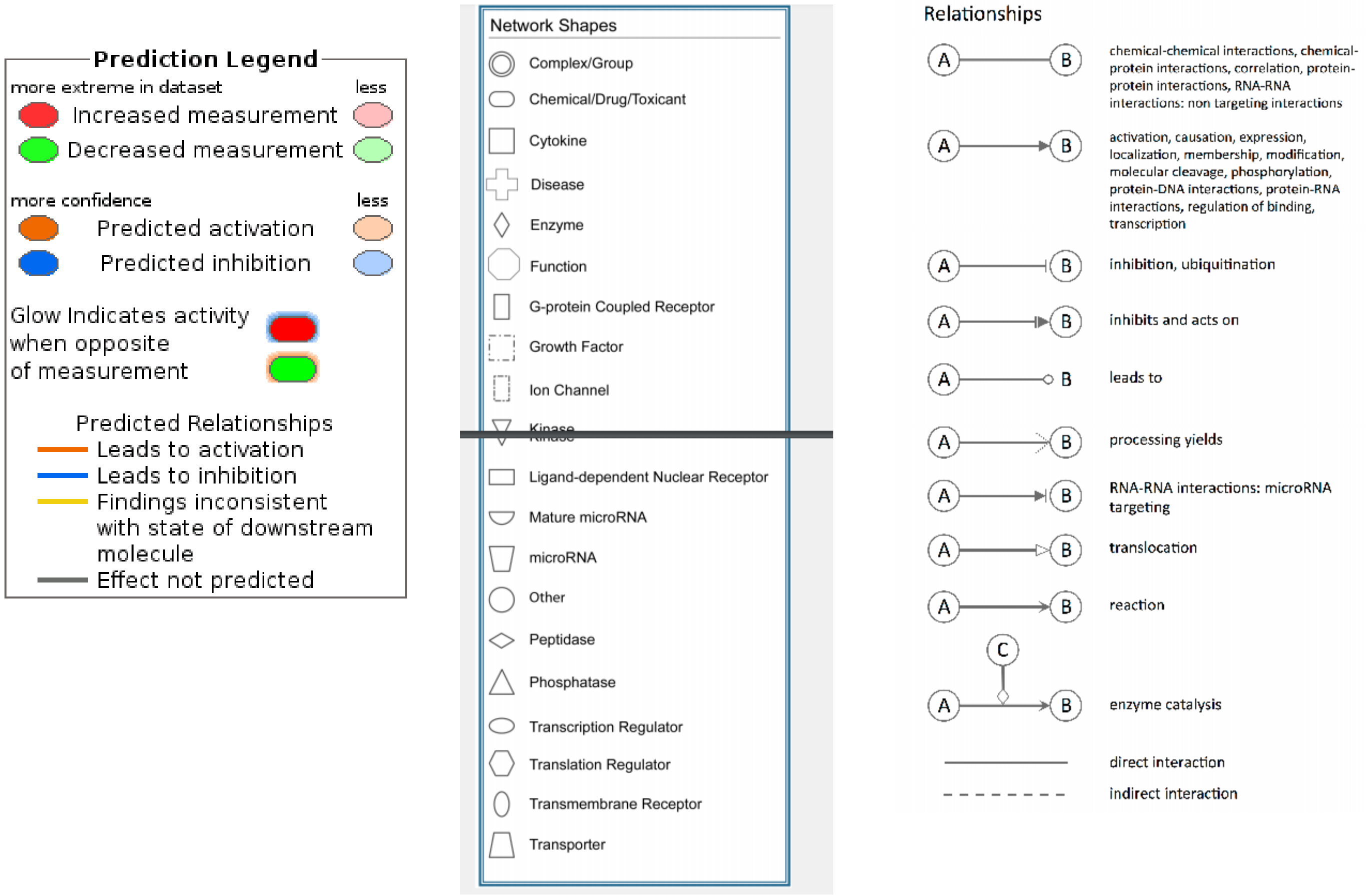

Fig S8. Network of the upstream regulator TGFB1 in MT-2 cells.

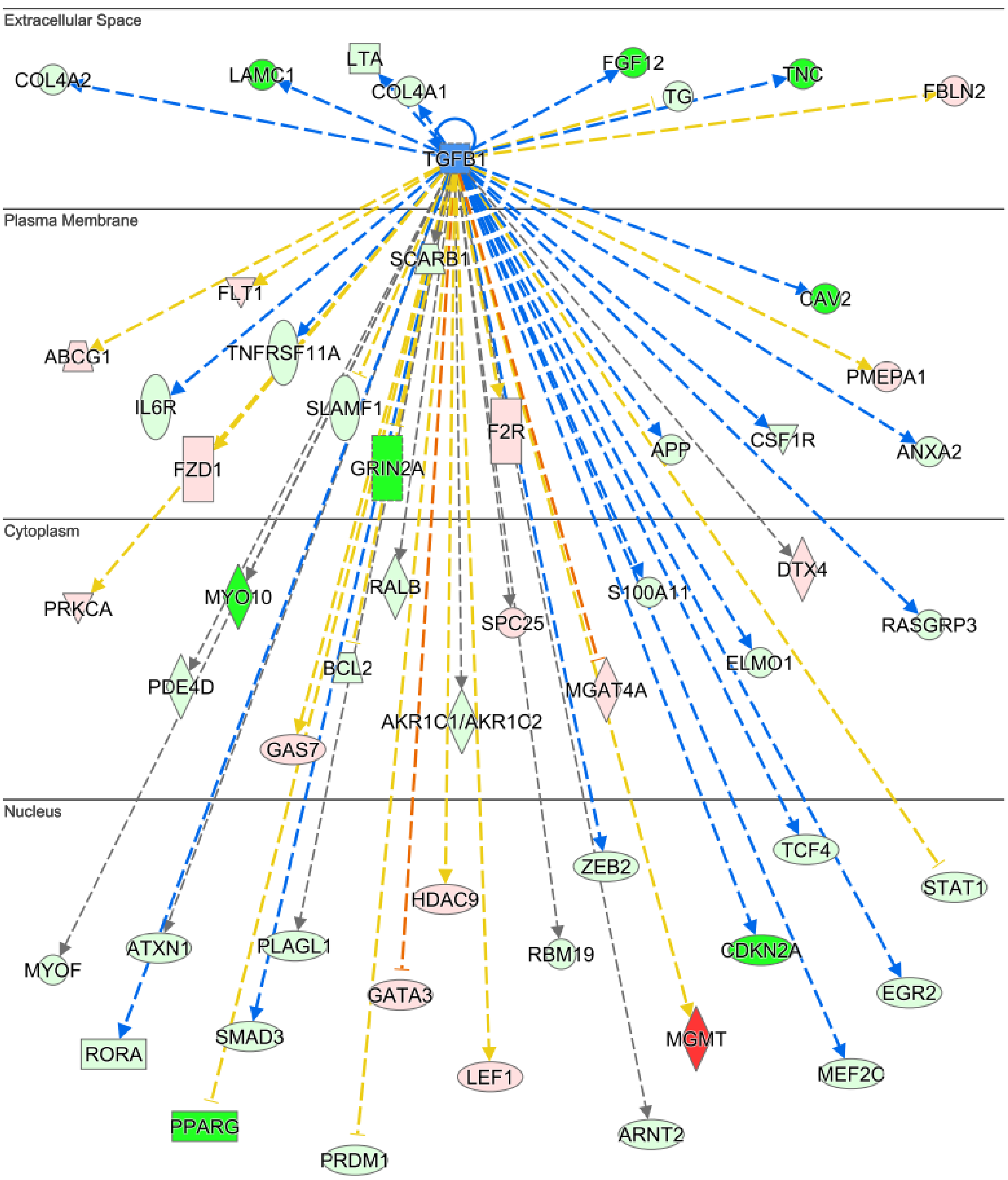

Fig S9. Network of the upstream regulator TGFB1 in Jurkat cells.

A

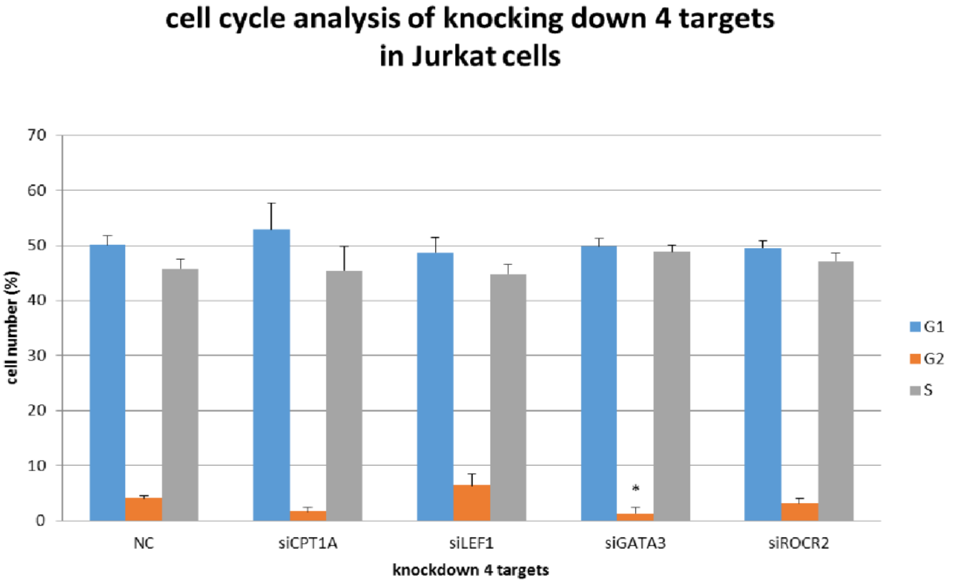

B

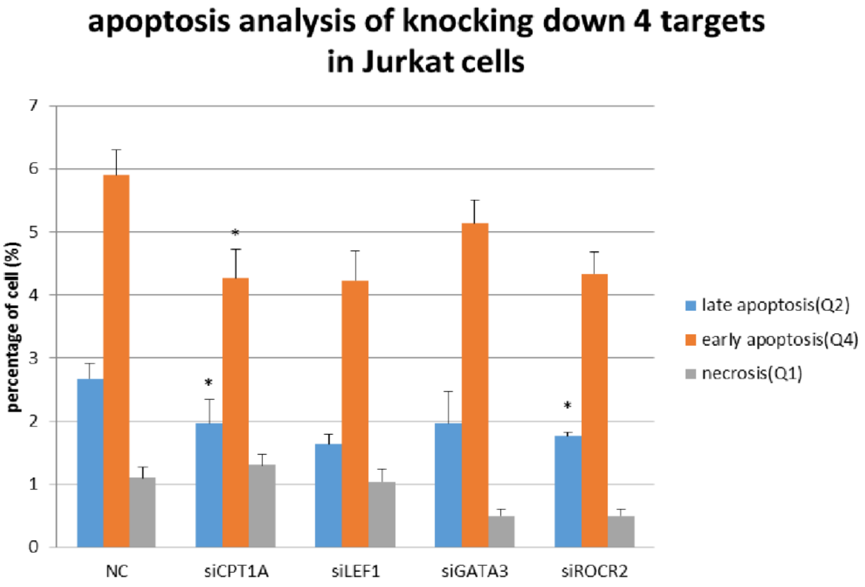

Fig S10. Effect of siRNA knockdown on cell viability and apoptosis.

(A) The effect of SiRNA knockdown on cell viability in Jurkat cell. Cell cycle distributions of knocking down 4 targets in Jurkat cells were verified by propidium iodide staining and analyzed by flow cytometry after 48 hours of siRNA knockdown. Three independent experiments were performed and * represents for p-value less than 0.05. **(B) The effect of siRNA knockdown on apoptosis in Jurkat cell.** Flow cytometry analyses of annexin V-propidium iodide double staining siRNA knockdown Jurkat cells. After 48 hour of siRNA knockdown, Jurkat cells were labelled with Annexin V -FITC at room temperature with 5μg/ml propidium iodide (PI). Annexin V positive –PI negative events (Q4) are early apoptotic cells,

double positive events (Q2) showing late apoptotic cells and double negative events showing necrosis cells (Q1). Three independent experiments were performed and * represents for p-value less than 0.05.

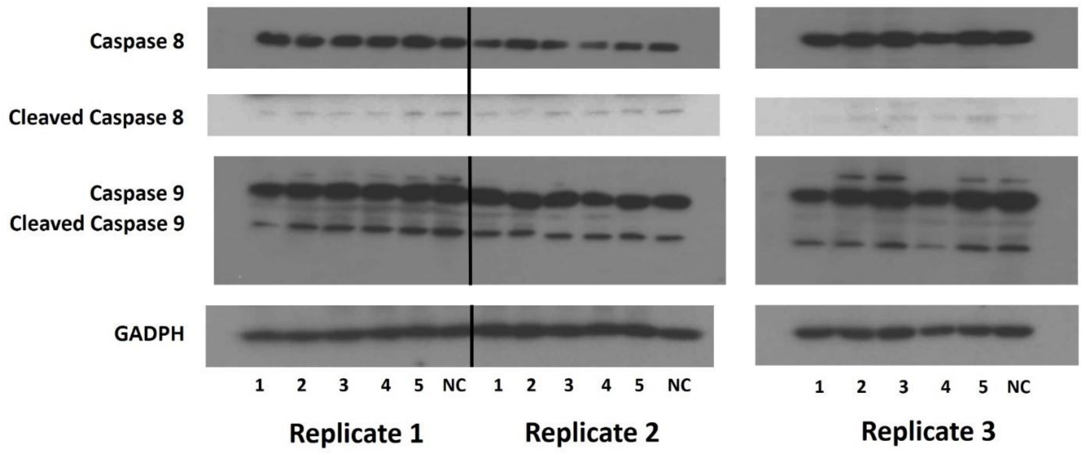

Fig S11. Western blot analysis after the knockdown of five target genes.

After 48 hours transfection, the protein was collected using RIPA (with proteinase inhibitor) for western blot. Caspase 8 (CST #9746) and caspase 9 (CST #9502) were detected using specific antibodies. Note that 1, 2, 3, 4 and 5 stands for ADM, CPT1A, GATA3, LEF1 and RCOR2, respectively, and "NC" stands for negative siRNA control.
