# Supplementary material for: Genome-Wide Characterization of Host Transcriptional and Epigenetic Alterations During HIV Infection of T Lymphocytes
Source: Front Immunol. 2020 Sep 10;11:2131. doi: 10.3389/fimmu.2020.02131 (PMC7511662; doi:10.3389/fimmu.2020.02131)
Supplement: Supplementary file 2 [file Data_Sheet_2.PDF]

Table of Contents

Table S1. Primer sequences used for RT–qPCR 3

Table S2. Functional enrichment of DMGs with hypermethylated DMRs in promoter regions in HIV+ samples of the MT-2 cell line. 4

Table S3. Functional enrichment of DMGs with hypermethylated DMRs in promoter regions in HIV+ samples of the Jurkat cell line. 5

Table S4. Functional enrichment of DMGs with hypomethylated DMRs in promoter regions in HIV+ samples of the MT-2 cell line. 6

Table S5. Functional enrichment of DMGs with hypomethylated DMRs in promoter regions in HIV+ samples of the Jurkat cell line. 6

Table S6. Primers used for capturing DMR sequences. 7

Table S7. Functional enrichment analysis of the upregulated DEGs in the MT-2 cell line. 7

Table S8. Functional enrichment analysis of the upregulated DEGs in the Jurkat cell line. 9

Table S9. GSEA Enrichment analysis in GSEA of Immune genes for the upregulated DEGs in the Jurkat cell line 14

Table S10. Functional enrichment analysis of the downregulated DEGs in the MT-2 cell line. 16

Table S11. Functional enrichment analysis of the downregulated DEGs in the Jurkat cell line. 22

Table S12. Downregulated genes enriched in the apoptosis pathway for the MT-2 and Jurkat cell lines. 27

Table S13. Upstream regulator prediction using the DEGs of HIV+ samples of the MT-2 cell line. 30

Table S14. Upstream regulator prediction using the DEGs of HIV+ samples of the Jurkat cell line 32

Table S15. DAVID functional enrichment for genes with differentially expressed exons in the Jurkat cell line. 33

Table S16. Upstream regulator prediction using overlapped genes between DEGs and DMGs in HIV+ samples of the MT-2 cell line 33

Table S17. Upstream regulator prediction using overlapped genes between DEGs and DMGs in HIV+ samples of the Jurkat cell line 35

Table S18. Demethylation treatment using 5-azac..36

Table S19. Expression levels of *CPT1A*, *TNF*, and *LEF1* in HIV infected and uninfected PBMC samples 36

Table S1. Primer sequences used for RT–qPCR

Gene	Forward Sequence (5’-3’)	Reverse Sequence (5’-3’)
SMAD3	CATCGAGCCCCAGAGCAATA	GTGGTTCATCTGGTGGTCACT
PDX1	CCTTTCCCATGGATGAAGTC	TTCAACATGACAGCCAGCTC
LGALS3	CCTTTGCCTGGGGGAGTGGTG	TGAAGCGTGGGTTAAAGTGGAAGG
TRAF1	AAGGGAGCTAGCCAGAGGAC	GTCCTGCCATCCTAACCAGA
GATA3	TGGGCTCTACTACAAGCTTCACAATAT	TTGCTAGACATTTTTCGGTTTCTG
RAN	ATGGTGGTACTGGAAAAACGAC	GGGATGTTTTCACACACTCGTA
HSP90AB1	TCCGGCGCAGTGTTGGGAC	TCCATGGTGCACTTCCTCAGGC
HSPA5	GGGAACGTCTGATTGGCGAT	CGTCAAAGACCGTGTTCTCG
GAPDH	CAAGGGCATCCTGGGCTAC	TTGAAGTCAGAGGAGACCACCTG

Table S2. Functional enrichment of DMGs with hypermethylated DMRs in promoter regions in HIV+ samples of the MT-2 cell line.

Category	Term	PValue	Genes	Fold Enrichment	Benjamini
KEGG_PATHWAY	hsa05203:Viral carcinogenesis	2.65E-05	TRAF1, GTF2E2, HIST1H2BN, ACTN4, HIST1H2BE, C3, HIST1H2BG, CCR3, PIK3CD, NFKBIA, NFKB1, YWHAE	4.87	0.005
KEGG_PATHWAY	hsa05168:Herpes simplex infection	2.92E-04	TRAF1, SRSF1, TNF, SRSF7, C3, CYCS, TAP1, NFKBIA, NFKB1, TAB2	4.55	0.027
GOTERM_MF_DIRECT	GO:0005515~protein binding	5.47E-04	ATP1B1, RPL13, SRP68, RADIL, ZNRF1, TGFB2, SFSWAP, GTF2E2, CRY2, KLHL6, ZSCAN9, MAP3K8, IZUMO1, SPRED1, RAPGEF1, RAB21, RDM1, CUTA, ACTN4, PIK3CD, CTNNA1, PROX1, MOXD1, SPAG9, RIOK3, MIB2, CCR3, TNFAIP8, TFAP2B, NEK8, PRDM1, MYO18A, DST, TRAF1, SRI, OSTF1, BLM, AKAP12, RRAGA, NFKBIA, NAPA, RCC1, UBAC2, DUSP19, DOCK2, BLOC1S2, GORASP2, ZNF223, OTUD7B, THAP10, KLF6, DIXDC1, RAB8B, SMG6, LGALS3, ASXL1, NR4A2, PLGRKT, MYL12A, HNRNPA1, TXNDC11, EIF4E, SRSF7, HIVEP1, PTGES3, SRSF1, BCKDK, LRRC8B, SORL1, NFKB1, ZBTB38, FUBP1, ARHGAP5, CDK12, LOXL3, MKL2, MAGEH1, NFKBIZ, CYCS, CCDC47, ATP6V1H, LRP2BP, MXD3, ELMO1, DOK1, MAST2, ZFYVE27, JAM2, PPP1R15A, STON2, TNF, C3, WRNIP1, AZIN1, NR3C1, ZNF652, USP17L2, SET, CEP57, TAP1, RWDD2B, WIPF2, CAMK2D, CERK, TNRC6A, RTCB, HIST1H2BE, HIST1H2BG, SCRN1, FZD1, NUP155, SGTB, TAB2, YWHAE, HYI, COG4, PEX11B, RGS20, IRF8, WHSC1L1, ATP6V0A1, GGA1	1.23	0.171
GOTERM_MF_DIRECT	GO:0016887~ATPase activity	0.001075	KIFC1, ATP1B1, BLM, WRNIP1, ATP6V1H, ABCA4, DST, MYO18A, RNF213	4.37	0.169
KEGG_PATHWAY	hsa05120:Epithelial cell signaling in Helicobacter pylori infection	0.00113	ATP6V0E2, NFKBIA, ATP6V0A1, ATP6V1H, NFKB1, JAM2	7.46	0.052
GOTERM_MF_DIRECT	GO:0003677~DNA binding	0.001826	TCEB3CL2, BLM, WRNIP1, HIST1H2AE, NFKB1, NR3C1, RAG2, ZNF652, PLAGL1, GTF2E2, TCEB3CL, SET, CRY2, HIST1H2BN, ZNF223, OTUD7B, THAP10, RDM1, KLF6, HIST1H2BE, HIST1H2BG, ZNF354B, ASXL1, NR4A2, PROX1, MXD3, ZFP37, MSL3, TCEB3C, TFAP2B, HIVEP1, ZNF432, MYO18A	1.75	0.189
GOTERM_MF_DIRECT	GO:0046982~protein heterodimerization activity	0.002278	SRI, HIST1H2BE, HIST1H2BG, HIST1H2AE, NR4A2, RRAGA, NFKB1, PDX1, CTNNA1, YWHAE, TGFB2, HIST1H2BN, TFAP2B, JAM2	2.67	0.178
GOTERM_BP_DIRECT	GO:0006405~RNA export from nucleus	0.003389	SRSF1, EIF4E, SRSF7, NUP155, HNRNPA1	8.03	0.987
GOTERM_CC_DIRECT	GO:0070449~elongin complex	0.003864	TCEB3CL2, TCEB3CL, TCEB3C	31.15	0.620
KEGG_PATHWAY	hsa05322:Systemic lupus erythematosus	0.004986	TNF, HIST1H2BN, ACTN4, HIST1H2BE, C3, HIST1H2BG, HIST1H2AE	4.35	0.091
GOTERM_CC_DIRECT	GO:0016020~membrane	0.005546	STON2, SRI, KIFC1, ATP1B1, TNF, RPL13, WRNIP1, SORL1, NAPA, ZNRF1, ABCA4, RNF213, DOCK2, ARHGAP5, ACSL1, GORASP2, TAP1, CAMK2D, NIPAL2, LOXL3, CUTA, NGEF, OSBPL5, LGALS3, FADS1, SPECC1, CCDC47, NUP155, HNRNPA1, YWHAE, ELMO1, PEX11B, ATP6V0E2, RBM19, GGA1, MYO18A, PPP1R15A	1.57	0.501

Table S3. Functional enrichment of DMGs with hypermethylated DMRs in promoter regions in HIV+ samples of the Jurkat cell line.

Category	Term	PValue	Genes	Fold Enrichment	Benjamini
GOTERM_MF_DIRECT	GO:0016887~ATPase activity	6.29E-04	ATP1B1, BLM, MACF1, ATP6V1H, ABCA4, DST, RNF213, HSPA8, ABCA5	4.74	0.184
GOTERM_MF_DIRECT	GO:0003700~transcription factor activity, sequence-specific DNA binding	9.17E-04	IKZF4, KAT7, ERG, IKZF3, LMO4, NR3C2, ZNF790, ZNF649, SMAD3, NR3C1, PDX1, MSC, PROX1, BATF, PLAGL1, NR1D2, HOXC5, NFAT5, PRDM2, PRDM1, ZNF432, RBPJ	2.21	0.138
GOTERM_MF_DIRECT	GO:0005515~protein binding	0.001093	ATP1B1, TBK1, RPL13, LMO4, RPS27L, ZNRF1, TGFB2, BATF, MAP3K8, IZUMO1, SPRED1, DISC1, RAB21, GNL3, CUTA, ACTN4, POLG, PIK3CD, ZNF649, DEPDC1, CTNNA1, PROX1, TRAPPC12, SPAG9, MIB2, CCR3, TNFAIP8, NEK8, CLIP1, PRDM1, DST, PNMA1, ALDOA, BLM, RRAGA, RCC1, UBAC2, DUSP19, PTK2, DOCK2, MACF1, GORASP2, MTCH1, OTUD7B, PBRM1, THAP10, KAT7, IKZF3, RAB8B, TAF8, KCTD1, SMAD3, MYL12A, ADPRH, RNF8, POLD3, ZC2HC1A, EIF4E, HIVEP1, RBPJ, LRRC8B, SIRPB1, RTN3, WARS, TRIM5, DIP2A, ARHGAP5, CDK12, LOXL3, FANCA, MAGEH1, NFKBIZ, CCDC47, ATP6V1H, LRP2BP, ECT2, DOK1, EFHC1, BVES, DACT1, UBC, SRGAP3, JAM2, RAB3C, NR3C2, NR3C1, ATXN2L, SET, NR1D2, RWDD2B, TAP1, WIPF2, CAMK2D, NFAT5, CERK, HSPA8, RTCB, ERG, ATXN7L1, FZD1, NUP155, SGTB, HYI, MGST3, PEX11B, RGS20, SMDT1, RASSF1, ATP6V0A1, CHN2, JAK2, GGA1	1.23	0.111
KEGG_PATHWAY	hsa04670:Leukocyte transendothelial migration	0.001571	PTK2, ARHGAP5, ACTN4, PIK3CD, MYL12A, JAM2, CTNNA1	5.47	0.248
KEGG_PATHWAY	hsa04142:Lysosome	0.001787	LIPA, AP3M2, ATP6V0A1, ATP6V1H, MCOLN1, ACP2, GGA1	5.33	0.149
GOTERM_BP_DIRECT	GO:0001816~cytokine production	0.001892	BATF, LIPA, PIK3CD, NFAT5	15.99	0.901
GOTERM_MF_DIRECT	GO:0005096~GTPase activator activity	0.002467	DOCK2, RGS20, ARHGAP5, SRGAP3, CHN2, DEPDC1, SYNGAP1, ECT2, ARHGAP23, ADPRH	3.46	0.181
GOTERM_CC_DIRECT	GO:0005737~cytoplasm	0.003097	TBK1, LRRC8B, UBE2G1, RNF213, BATF, WARS, TRIM5, ARHGAP5, MAP3K8, MCOLN1, LOXL3, SPRED1, FANCA, MAGEH1, SERTAD2, ACTN4, EFCAB13, MICAL3, LRP2BP, RFTN1, ECT2, PROX1, DOK1, SPAG9, EFHC1, DACT1, MIB2, EEF1A1P5, TNFAIP8, UBC, NEK8, SRGAP3, CLIP1, PRDM1, DST, PNMA1, BLM, RRAGA, WARS2, NR3C1, RCC1, ALDH1A2, PTK2, FAM117A, ATXN2L, SET, MACF1, ANKRD37, CAMK2D, NFAT5, OTUD7B, RTCB, KAT7, ERG, IKZF3, UBE4B, SMAD3, LRRC49, RNF8, RGS20, EIF4E, RASSF1, PTP4A2, CHN2, RBM19, ATP6V0A1, JAK2, RBPJ, SYNGAP1	1.35	0.522
GOTERM_CC_DIRECT	GO:0005634~nucleus	0.00314	RPL13, RPS27L, BATF, WARS, TRIM5, DIP2A, CDK12, RAPGEF5, LOXL3, FANCA, DISC1, SERTAD2, GNL3, NFKBIZ, ACTN4, EFCAB13, SPECC1, MICAL3, ZNF790, ZNF649, DEPDC1, H2AFJ, CYB5RL, ECT2, PROX1, MSC, TRAPPC12, DOK1, DACT1, EEF1A1P5, UBC, PRDM2, PRDM1, ZNF432, DST, ALDOA, BLM, NR3C2, RRAGA, NR3C1, RCC1, PLAGL1, PTK2, SET, NR1D2, ANKRD37, NFAT5, CAMK2D, OTUD7B, AUTS2, HSPA8, RTCB, IKZF4, KAT7, ERG, IKZF3, TAF8, UBE4B, KCTD1, SMAD3, POLD3, RNF8, RGS20, R3HDM2, SMDT1, RASSF1, PTP4A2, RBM19, JAK2, HIVEP1, RBPJ	1.34	0.312
GOTERM_BP_DIRECT	GO:0033572~transferrin transport	0.005006	ATP6V0E2, ATP6V0A1, ATP6V1H, MCOLN1	11.42	0.870
GOTERM_CC_DIRECT	GO:0005765~lysosomal membrane	0.005414	SNX14, UBC, RRAGA, ATP6V1H, MCOLN1, ACP2, WDR41, HSPA8, ABCA5	3.36	0.350
GOTERM_MF_DIRECT	GO:0042803~protein homodimerization activity	0.008622	IKZF4, IKZF3, ACTN4, RRAGA, SMAD3, ECT2, TGFB2, RNF8, PEX11B, TRIM5, TAP1, CAMK2D, CLIP1, IZUMO1, SLC51A, DST	2.11	0.428

Table S4. Functional enrichment of DMGs with hypomethylated DMRs in promoter regions in HIV+ samples of the MT-2 cell line.

Category	Term	PValue	Genes	Fold Enrichment	Benjamini
GOTERM_BP_DIRECT	GO:0060391~positive regulation of SMAD protein import into nucleus	0.001731842	DAB2, RBPMS, BMPR1A	47.26	0.684
GOTERM_BP_DIRECT	GO:0003215~cardiac right ventricle morphogenesis	0.001731842	GATA3, HEY2, BMPR1A	47.26	0.684
GOTERM_BP_DIRECT	GO:0007411~axon guidance	0.007321372	ANK3, GATA3, NFASC, CNTN4, FEZ2	6.44	0.913
GOTERM_MF_DIRECT	GO:0042803~protein homodimerization activity	0.008153124	RBPMS, GRIK2, STK10, HEY2, ABCD3, MYO9B, PCYT1A, IL6R, BMPR1A, FOXP2	2.82	0.801

Table S5. Functional enrichment of DMGs with hypomethylated DMRs in promoter regions in HIV+ samples of the Jurkat cell line.

Category	Term	PValue	Genes	Fold Enrichment	Benjamini
GOTERM_MF_DIRECT	GO:0008134~transcription factor binding	4.37E-05	CDKN2A, HEY1, DACT2, HTT, GATA3, HEY2, LEF1, RORA, NLRP3, TCF7L2, NFIA	5.27	0.012
GOTERM_CC_DIRECT	GO:0005783~endoplasmic reticulum	5.50E-04	GABARAPL1, ATP10B, USP17L26, HTT, ARSG, GRIN2A, PDIA6, USP17L11, RHBDD1, NLRP3, CLDN14, STAU2, USP17L21, ACSBG1, PHEX, MGST1	2.79	0.086
GOTERM_BP_DIRECT	GO:0046825~regulation of protein export from nucleus	0.001378753	XPO1, CDKN2A, PTPN14	52.04	0.710
GOTERM_MF_DIRECT	GO:0005522~profilin binding	0.001833402	HTT, EVL, PCLO	45.38	0.219
GOTERM_BP_DIRECT	GO:0032753~positive regulation of interleukin-4 production	0.008846294	CD3E, GATA3, NLRP3	20.82	0.981
GOTERM_BP_DIRECT	GO:0045892~negative regulation of transcription, DNA-templated	0.009577122	DAB2, CDKN2A, ZNF217, HEY1, GATA3, HEY2, LEF1, SFMBT1, TCF7L2, FOXP2	2.78	0.944
GOTERM_BP_DIRECT	GO:0001676~long-chain fatty acid metabolic process	0.009732125	ACSL6, CPT1A, ACSBG1	19.83	0.888

Table S6. Primers used for capturing DMR sequences.

LEF1-Forward	TTTATTTTAATGGTAGAGGTGAGTA
LEF1-Reverse	CAAAAAACCTATATTTTAAATATTATTC
TBX3-Forward	TAYGTTTTAGGATAAGGAAGAGAGAGAGG
TBX3-Reverse	AAAAAAAATCTAATTTAAACCCCCTTCTACCAA
CPT1A-Forward	TTGAGGTAAAATTAATTTAAAGAATTAGTT
CPT1A-Reverse	TTCTATTTATACCAAAAAACACCCC
NTRK2-Forward	TTGTGTGTGTGTGTGTATTTATATTTAGG
NTRK2-Reverse	AATAACTTACCCTCCCTTTTAAACAAA
GATA3-Forward	ATTTTTTTTGGTTGTAGTTTAGTTAAGTAA
GATA3-Reverse	TATAAACACAAAATATATATACATAAACCT
PDX1-Forward	ATGTTTTTTTGTAAATTATATTAATTAATAAA
PDX1-Reverse	CAACTAAATTTACTACACACTCCTAAAC

Table S7. Functional enrichment analysis of the upregulated DEGs in the MT-2 cell line.

Category	Term	PValue	Genes	Fold Enrichment	Benjamini
KEGG_PATHWAY	hsa05340:Primary immunodeficiency	1.35E-07	PTPRC, CD19, CD3D, CD3E, CD40LG, LCK, RAG1, ZAP70, CD79A, RAG2, IL7R, RFXAP, ADA	6.84	0.000
KEGG_PATHWAY	hsa04640:Hematopoietic cell lineage	3.26E-06	IL4, CD3G, CD3D, CD3E, CD1C, CD1B, CD1A, ITGA3, KIT, IL7R, CD1E, CD1D, CD19, DNTT, GP1BB, ITGA5, CD5, CD7	3.79	0.000

GOTERM_CC_DIRECT	GO:0005622~intracellular	1.52E-05	ADCY4, HKR1, ADCY7, EFNA1, TLR3, RGL4, ZNF778, TRIM47, MAP3K9, ARL15, ZNF107, MAP2K6, ZNF43, ZNF595, ZNF45, BCR, DFFB, ZNF793, TRAT1, PITPNM2, SIRPG, ZNF439, TRIM36, CD40LG, RAB15, SLC40A1, UNC13B, DBN1, ZNF519, ZNF844, BBS5, RALGPS1, ADORA2B, ANAPC15, ASB13, ADCYAP1, RASAL1, OSBPL1A, PLCH2, NKX3-1, ZNF74, ZNF124, ZNF599, RHOBTB3, ZNF527, ZNF69, MDFI, VAV3, RGPD2, DOCK7, ZNF320, ZNF585A, ZNF585B, DOCK3, TRAF3IP2, HEATR3, PLCG2, PARP8, SYTL1, FGF5, ZFP14, PREX2, RHOU, ZNF682, TRIM6, ZNF726, RASL10B, ZNF681, GUCY1A3, ZNF540, ANO5, ARL5C, DOCK10, DOCK11, ANO10, PRKCA, MPP1, ARHGEF6, ZNF354B, ZNF91, PRKCH, ZNF549, SH2D3C, KSR2, PLXDC1, CAPN14, CAND2, GUCY1B3, ZNF480, KCNH2, GSTP1, ZNF485, TBC1D8, ZNF557, TRIM14, ABI3, SEC14L5, TRIM6-TRIM34, POLR2D, ZNF169, PLCL1, RAB44, STK32B, DGKD, RASGRP2, HSD17B6, TBC1D4, ZSCAN25, SCN5A, DGKQ, ZNF569, RCAN1, ZNF26, COTL1, CDC25A, CD19, GMFG, MMP23B, GFRA1,	1.46	0.008

			CHN2, EVI5L, ZNF570, CIT, ZNF571, SNTA1		

Table S8. Functional enrichment analysis of the upregulated DEGs in the Jurkat cell line.

Category	Term	PValue	Genes	Fold Enrichment	Benjamini
GOTERM_CC_DIRECT	GO:0005886~plasma membrane	7.86E-09	SLC9A9, ADCY4, SCN3A, EFNA1, SYT5, C6ORF25, UTRN, ADORA1, AQP3, S1PR3, ADTRP, KCNK7, OSCAR, PRRT1, DYNC2H1, CHRNA7, GNG2, IZUMO1, KLRD1, CHRNA3, MVB12B, GNG7, CDH23, ARC, CD3G, PRAME, EFNB3, CD3D, CD3E, EFNB1, CDHR1, VANGL2, PTPRS, LIG4, WNK2, PLAUR, SIRPG, SSTR3, DOC2A, MAGEE1, LPAR5, CX3CR1, ERVH48-1, TMEM184A, RAB15, MADCAM1, RAB13, UNC13B, DBN1, SH3GL2, ITGA2B, PALM, MME, RUNDC3A, CACNB4, CD72, PXN, ADA, EPHB2, CAMKV, SERINC5, MACF1, SLC29A4, DNAAF1, ZAP70, RAP1GAP2, CNKSR1, CELSR2, ATP11C, ABCG4, HOMER2, ABCG1, EPHA3, NOTCH3, TNKS1BP1, KIAA0922, CHRNB4, RGS9, TREML2, SYTL1, GPR162, MARCKSL1, KCNJ10, LY9, SIRPB1, KCNIP3, SLC23A1, SPG20, SYNJ2, GUCY1A3, KCNG3, FCGR3A, JPH1, FCGR3B,	1.32	0.000

			DPP4, IFNLR1, C10ORF54, GPR174, STX3, ATP4A, ZP3, SDK2, RPH3AL, ACKR2, ARRDC4, FLNC, ALOX15, DOK3, CCND3, SEMA4G, SLC26A7, LAX1, PLEKHH2, PLXDC1, EPB41L5, CYBRD1, CCNYL1, SORT1, GRIP2, TCHP, GUCY1B3, GNB3, KCNH2, ARL4C, GRASP, SEMA4A, RASD2, AVPR2, RAB3B, LIMS2, GPR61, FHL1, PI4KAP1, GCSAM, ZBTB16, GPR68, KIT, IL7R, ITM2B, ITM2C, GJC1, ITM2A, PLCL1, RAB44, KIRREL, FAT1, CLEC2B, ACSL6, CD28, FYB, SELL, CD1C, RTN4R, FZD1, CD1B, CD1A, CD1E, CD1D, FZD6, PROM1, P2RY10, CD19, DRP2, LIME1, GFRA1, TJP3, BAMBI, ABCC5, DNM1, ABCC6, LRP5, GPRIN1, ENAH, GABRB3, RAB9B, SLC7A8, IL17RE, TLR5, TLR7, VIPR2, IL17RA, RELL1, RELL2, NRCAM, PACSIN3, PDGFC, SLC4A4, INSR, SPN, SCAMP5, TUB, TMEM204, ACTN2, KIAA0754, IRS1, TRAT1, HCST, FNBP1L, RIPK3, DSP, EDA, SLC40A1, IGSF9, PTGDR2, C1ORF186, FXYD2, ITGAL, SLC38A3, ADORA2B, IFITM1, IFITM2, TMIGD2, ABCA1, FXYD6, LLGL2, RASAL1, PPP1R16B, SLC48A1, PLCH2, TTYH1, APOBR, CNR2, PLCH1, LRFN1, RHOBTB1, MFAP3L, LPL, CUBN, VAV3, TBC1D10C, MYO1B, KCNB1, TNFRSF13B, LIN7B, NIPA1, SHANK3, CACNA2D4, CORO1A, LAMP3, P2RX1, ITGA5, PLCG2, ANTXR2, CD79A, DIO1,		

			ANTXR1, SCN8A, PIP4K2A, FAM84B, CACNA1A, LRRC8C, LRRC8D, PREX2, MTSS1L, LGR6, RHOU, SKAP1, PDCD1, STXBP5L, ACVR1B, KCNQ4, GPC2, VN1R1, GPC3, GSN, RASL10B, SMAGP, TPO, DLG3, ESAM, LTB, ATP8B3, NSF, ANO9, RHOH, PRKCA, LAIR1, TRPM5, SIT1, COL23A1, PTGER3, PTGER4, SLA2, STXBP1, SPINT1, PRKCH, PCDH9, PRKCG, SLC9A3R2, TRPM2, CD84, ADRB1, CA9, CPAMD8, LCK, CTSG, PMEPA1, GSTP1, TNFRSF21, CD244, CAMK2G, CD247, APH1B, CYTH3, CDH4, SCARF1, TNFRSF1A, RGMA, HRH1, RGMB, GP1BB, RASGRP2, KRT1, SH2B2, GPNMB, CD5, SELPLG, CD7, RNF144A, PTPRC, GLRB, FLT1, FLT4, TSPAN14, ANXA1, CD99, ASIC1, USP6NL, SLC17A7, SLC6A8, IFIT5, KCNN2, TAS2R31, F2R		
GOTERM_CC_DIRECT	GO:0005887~integral component of plasma membrane	6.84E-07	MPZL1, CADM4, TSPAN2, GABRB3, EFNA1, KIAA1324, SLC7A8, TRGC2, TSPAN7, CD52, IL17RE, TLR5, CSPG5, AQP6, VIPR2, TLR7, ADORA1, AQP3, IL17RA, NRCAM, S1PR3, ST3GAL5, KCNK7, SLC4A4, INSR, SPN, PTPRK, TYRO3, TMEM150C, CD3G, EFNB3, CD3E, EFNB1, CDHR1, PTPRS, TRAT1, PLAUR, VSIG2, SSTR3, LRP12, CX3CR1, EDA, VAMP1, SLC40A1, PTGDR2, ITGA2B, PALM, ORAI1, SLC38A3, CALY, ADORA2B, MME, ABCA1, CD72, ABCA4, EPHB2, CD69, CNR2, SLC31A2, TNFRSF13B,	1.53	0.000

			ATP13A2, ABCG1, GUCY2D, EPHA3, SEMA6C, SLC4A11, P2RX1, ATP2A3, CHRNB4, CD79A, NKG7, LRRC8C, LRRC8D, SORL1, KCNJ10, LGR6, SIRPB1, ACVR1B, SLC23A1, GPC3, SMAGP, TPO, EVI2B, HHIP, PTGER3, SIT1, ATP4A, MPP1, PTGER4, ACKR2, TRPM2, CD84, ADRB1, SLC26A7, GPA33, AVPR2, TNFRSF21, APH1B, GPR68, CDH4, TNFRSF1A, HRH1, PRRG1, GP1BB, CLEC2B, FAT1, SLC18B1, HS6ST1, GPNMB, CD6, SELPLG, CD5, CD28, PTPRC, GLRB, FLT1, SELL, FLT4, TSPAN14, RTN4R, CD1C, CD99, CD1A, ASIC1, CD1E, FZD6, CD1D, PROM1, P2RY10, CD19, SLC6A8, ABCC5, F2R		
KEGG_PATHWAY	hsa05340:Primary immunodeficiency	1.09E-06	PTPRC, ORAI1, CD19, CD3D, CD3E, TNFRSF13B, LCK, ZAP70, CD79A, IL7R, RFXAP, ADA	6.40	0.000
KEGG_PATHWAY	hsa04640:Hematopoietic cell lineage	2.73E-06	CD3G, CD3D, CD3E, CD1C, MME, CD1B, CD1A, KIT, IL7R, CD1E, CD1D, CD19, DNTT, GP1BB, ITGA5, CD5, CD7, ITGA2B	3.84	0.000
GOTERM_MF_DIRECT	GO:0003779~actin binding	4.92E-07	ABLIM1, ENAH, WASF3, MYO7B, MARCKSL1, WASF1, UTRN, GCSAM, TPM2, DAAM2, SYNPO2L, MACF1, GSN, FBXO25, SNTB1, MICAL1, PHACTR1, CLMN, MYO1F, EVL, COTL1, TNNI3, MYOZ3, HOMER2, SHANK3, MSRB2, TNS4, SYNE1, CORO1A, SYNE2, PLEKHH2, GMFG, CAPG, SPTBN2, TMOD2, SPTBN1, DBN1, EPS8L1, MYLK, SNTG2, SNTA1	2.39	0.001

GOTERM_CC_DIRECT	GO:0042105~alpha-beta T cell receptor complex	6.77E-05	PTPN6, CD3G, CD3D, CD3E, CD247	16.24	0.006
GOTERM_CC_DIRECT	GO:0042101~T cell receptor complex	5.71E-05	CD3G, CD3D, CD3E, CD247, ZAP70, CD6, SKAP1, TRAT1	7.22	0.006
GOTERM_CC_DIRECT	GO:0009897~external side of plasma membrane	4.79E-05	CD244, RTN4RL1, KIT, ABCA1, IL7R, ADA, PDCD1, NRCAM, CD69, IGLL1, CHRNA7, RTN4RL2, FCGR3A, KLRD1, CD5, LAG3, SPN, CD28, PTPRC, SELL, CD3E, TNFRSF13B, ABCG1, CD19, ITGA5, AMOT, ANTXR2, CD79A, ANTXR1, ITGA2B	2.29	0.006
GOTERM_CC_DIRECT	GO:0016323~basolateral plasma membrane	4.23E-05	PALM, ORAI1, FXYD2, SLC38A3, SLC7A8, KCNJ10, ADORA1, AQP3, DISP1, DLG3, STK39, SLC4A4, MYO1A, VANGL2, ANXA1, LIN7B, CTNNA2, CD1D, SLC4A11, CA9, SLC26A7, OTOF, DSP, HPGD, SLC40A1, PIANP, ABCC6	2.44	0.007
GOTERM_CC_DIRECT	GO:0005622~intracellular	1.15E-04	ADCY4, HKR1, ZNF823, EFNA1, SH2D6, MAP3K9, ZNF773, ARL15, ZNF772, ZNF107, ZNF43, ZNF595, TRAT1, DEPDC4, PITPNM2, SIRPG, ZNF439, TRIM36, RAB15, RAB13, NYAP1, SLC40A1, DBN1, PTGDR2, UNC13B, ZNF519, ADORA2B, ASB13, ZNF615, ADCYAP1, RASAL1, ZNF429, OSBPL1A, PLCH2, NKX3-1, ZNF74, RHOBTB1, ZNF599, RHOBTB3, MDFI, ZNF624, VAV3, KCNB1, DOCK7, ZNF320, SHANK3, DOCK3, ZNF419, PLCG2, SYTL1, KRBOX1, ZFP14, PREX2, RHOU, STAC2, TRIM6,	1.41	0.008

			ZNF726, RASL10B, ZNF681, GUCY1A3, ZNF540, ARL5C, ZNF493, ANO9, RHOH, PRKCA, ZNF283, STX3, MPP1, PTGER4, ZNF91, RPH3AL, PRKCH, PRKCG, ZNF549, SH2D3C, KSR2, PLXDC1, CAND2, GUCY1B3, ZNF480, KCNH2, ZNF33B, RASD2, GSTP1, ZNF485, TBC1D8, ABI3, PI4KAP1, TRIM6-TRIM34, ZNF169, PLCL1, RAB44, STK32B, RASGRP2, HSD17B6, ZSCAN25, TBC1D4, SH2B2, CAPN5, ZNF569, EXPH5, RCAN1, COTL1, ZNF662, CD19, TOM1L1, GMFG, MMP23B, ZSCAN32, ZNF461, CHN2, GFRA1, EVI5L, ZNF571, SNTA1		

Table S9. GSEA Enrichment analysis in GSEA of Immune genes for the upregulated DEGs in the Jurkat cell line

NAME	SIZE	ES	NES	NOM p-val
GSE2405_0H_VS_1.5H_A_PHAGOCYTOPHILUM_STIM_NEUTROPHIL_DN	184	0.67	1.46	4.88E-03
GSE37532_WT_VS_PPARG_KO_VISCERAL_ADIPOSE_TISSUE_TREG_UP	171	0.67	1.45	0.00E+00
GSE27241_CTRL_VS_DIGOXIN_TREATED_RORGT_KO_CD4_TCELL_IN_TH17_POLARIZING_CONDITIONS_UP	169	0.67	1.44	9.26E-03
GSE17301_ACD3_ACD28_VS_ACD3_ACD28_AND_IFNA5_STIM_CD8_TCELL_UP	182	0.65	1.39	4.72E-03
GSE11057_NAIVE_VS_EFF_MEMORY_CD4_TCELL_UP	169	0.64	1.39	9.62E-03
GSE17301_CTRL_VS_48H_ACD3_ACD28_IFNA2_STIM_CD8_TCELL_DN	179	0.64	1.38	2.62E-02

GSE1460_DP_VS_CD4_THYMOCYTE_UP	172	0.62	1.36	1.32E-02
GSE2405_HEAT_KILLED_LYSATE_VS_LIVE_A_PHAGOCYTOPHILUM_STIM_NEUTROPHIL_9H_DN	178	0.62	1.35	1.87E-02
GSE33425_CD8_ALPHAALPHA_VS_ALPHABETA_CD161_HIGH_TCELL_DN	182	0.61	1.33	1.97E-02
GSE2405_S_AUREUS_VS_UNTREATED_NEUTROPHIL_DN	185	0.61	1.32	9.30E-03
GSE11057_EFF_MEM_VS_CENT_MEM_CD4_TCELL_DN	157	0.62	1.31	3.85E-02
GSE30962_PRIMARY_VS_SECONDARY_ACUTE_LCMV_INF_CD8_TCELL_UP	182	0.61	1.31	2.65E-02
GSE21670_IL6_VS_TGFB_AND_IL6_TREATED_CD4_TCELL_DN	170	0.60	1.30	4.30E-02
GSE15750_DAY6_VS_DAY10_EFF_CD8_TCELL_UP	182	0.61	1.30	3.11E-02
GSE22886_NAIVE_CD4_TCELL_VS_MONOCYTE_UP	175	0.59	1.29	4.04E-02
GSE11057_NAIVE_VS_MEMORY_CD4_TCELL_UP	163	0.60	1.29	2.37E-02
GSE40274_CTRL_VS_FOXP3_TRANSDUCED_ACTIVATED_CD4_TCELL_UP	168	0.58	1.27	3.49E-02
GSE14769_UNSTIM_VS_120MIN_LPS_BMDM_UP	160	0.59	1.26	4.08E-02

Table S10. Functional enrichment analysis of the downregulated DEGs in the MT-2 cell line.

Category	Term	PValue	Genes	Fold Enrichment	Benjamini
KEGG_PATHWAY	hsa04514:Cell adhesion molecules (CAMs)	1.01E-14	CLDN4, CADM1, CLDN5, LRRC4B, HLA-DMB, SDC4, HLA-DMA, SDC2, NEGR1, LRRC4, ICAM1, PTPRM, NRXN3, HLA-A, HLA-C, NRXN1, CD40, HLA-B, HLA-E, HLA-DQA2, HLA-DQA1, HLA-F, SDC1, CD86, CD80, CLDN1, VCAN, HLA-DPA1, JAM2, CD226, HLA-DRA, HLA-DQB1, HLA-DRB1, CDH1, CDH3, VCAM1, ALCAM, ITGB8, CD22, HLA-DRB5, CD4, HLA-DPB1, HLA-DOA, MAG, CD276, CTLA4, NLGN2, ITGA9, CD274, CD58	3.32	2.72E-12

GOTERM_CC_DIRECT	GO:0005886~plasma membrane	3.72E-13	SGMS2, OSMR, SLC9A3, MYLIP, CTNNB1, APP, DIRAS2, AQP11, SERPINE1, OGT, FAS, ADAM8, GNG4, PTPRJ, ARC, GRIN2A, PTPRR, ZHX2, CDHR3, SPIRE1, CDHR5, HLA-DQA2, MARK1, HLA-DQA1, THY1, F5, RYR1, TFPI, RYR2, ROR2, HLA-DPA1, DOC2B, CD226, HLA-DRA, IL1R2, HLA-DRB1, CYP51A1, GNAI1, ARHGEF28, NFKBIA, DAAM1, DAGLA, CDC42EP1, UPK1A, OR56B1, HLA-DRB5, HLA-DPB1, CDC42EP4, HLA-DOA, CDC42EP5, TECTA, IL23R, PCDH15, RGS1, RGS2, ERVK13-1, PKP2, RGS3, RGS6, SYTL2, MARCKS, SYTL3, CHRNE, CDC42BPB, GPR85, MSR1, KCNAB1, ATP10A, TRPV3, PDIA6, ST6GALNAC6, SLC22A4, GUCY1A2, SNAP47, CEACAM6, DPP6, IL13RA1, CEACAM1, KCNG1, FMNL1, AR, LYN, CFTR, MMP16, IL6R, RFTN1, NAALAD2, CARD14, CLIC6, TNFSF12-TNFSF13, THEM4, RASD1, KCNH4, ENOX1, HLA-DQB1, HLA-DQB2, C3, FPR1, DISP2, ACSL1, DAPP1, SOS1, DGKG, VMP1, SNAP25, FLRT3, OR2I1P, CTLA4, DGKH, CAPN2, TMPRSS6, LSP1, RASSF3, LRP1, PTP4A3, PERP, SLC8A3, KCNC3, GRIK2, SLC6A20, TNFSF14, RRAD, TNFSF12, SLC2A6, ANK1, UNC5B, ANK2, ANK3, SLC2A3, RALB, IL15RA, SLC51A, EBI3, CIB1, IRS2, DYX1C1, HLA-A, HLA-C, HLA-B, HLA-E, HLA-F, THBD, VAMP8, REM2, C2CD4C, NEU1, NFE2L2, PTGFRN, DCUN1D3, LRRK2, XRN1, PAM, ACHE, LITAF, DRD2, TNFRSF12A, PANX2, MRAS,	1.32	2.49E-10

			LRIG3, ABCA3, SLCO2A1, IL12RB2, AMER2, ADAM28, B4GALT1, CACNA2D1, TLR10, MYO1C, ADAM23, SYT11, SLC6A12, MET, NTN4, FURIN, TRADD, MYO10, P2RX6, GPR37, FREM2, SLC7A2, PRKAR1B, CXCL16, CACNA1G, ADAM20, CACNA1E, CACNA1F, CACNA1C, TRIP6, SYT17, CACNA1A, CACNA1B, CLDN4, PREX1, UCHL1, DUOX2, CLDN5, SDCBP2, SDC4, SKAP2, SDC2, SLC1A3, FRMD6, GPC6, RHOB, CSF2RB, ANO7, NRG2, AHNAK, VASN, EVA1A, TRPM8, CNTN5, ESR1, PCDH8, PCDH7, SIGLEC14, VASP, SDC1, ADRB2, CHRM4, STXBP6, CHRM2, SLC41A2, CA2, JAM2, VIM, C2CD4D, OXTR, CDH1, CDH3, ALDH3A1, CDH8, VCAM1, CD9, CHD9, ANXA8, STK32A, TSPAN33, CD4, NFATC2, EHD4, TCIRG1, SLC2A9, MAG, TNFSF4, SLC12A1, KL, SAMHD1, TNFSF9, TAB2, ANXA2, HSP90B1, DSC3, SCN4B, PLEKHN1, OPRD1, SYT1, ADCY1, ATP1B1, CADM1, IL6ST, ADCY6, SYT8, LPAR3, AQP7, SYT7, S1PR1, CD44, S1PR4, ISLR2, PLD2, MDGA1, EFNB2, PIM1, GPR132, CD40, LILRB1, CCR9, CCR7, HAS1, CCR4, EFNA5, ADD2, ENPP2, GSDMB, CATSPERG, AKAP12, IFI30, CD70, AFAP1L2, CD74, AGRN, STX11, GAD1, CNKSR2, KLF5, TRPC1, IKZF3, LGALS3, PLB1, KLF9, IL1RL1, CFB, SMAD7, OSBPL6, PTPN13, SMAD3, MCAM, EPHA2, GPR153, ABCG2, NOTCH2, EPHA7, CD55, SEMA6D, SFRP1, NTRK1, CD58, PON2, PDZK1,		

			TAS2R4, SLC15A1, BCAR1, TAS1R3, CD151, TAS1R2, FCRL5, FCRL3, TNFRSF11A, SLC24A3, FAM129A, KCNMA1, ICAM1, GPR176, ASTL, ICAM5, ACKR3, NFAM1, ACKR4, STX1B, TNFRSF10A, TNFRSF10C, TNFRSF10B, TNFRSF10D, GRM7, PSEN2, SEMA4B, SGCE, SUSD3, PRF1, SLC39A14, SHROOM2, TPCN1, FNDC4, PLIN2, CLEC2D, CD22, ETV5, FAM127A, TES, IL18R1, BAIAP2L1, HSPG2, ATP1A4, GRIA3, FZD3, GRIA4, MAPK10, NPR3, FZD5, FZD4, STAT3, CISH, SLC16A3, TENM4, TJP1, SLC16A7, TENM2, ABCC4, PTCH1, SLC22A16, TLR1, ILDR1, TLR6, WNT2, ROBO1, CSF3R, MCOLN2, NALCN, NEGR1, PCDHGA12, PCDHGA10, PCDHGA11, MYO6, TNFRSF14, BASP1, NPEPPS, IGSF8, PLCE1, EMP2, EMP1, WNT5A, CAV2, TNFAIP8L3, SNX9, CAV1, SNAP91, TBC1D3F, PAQR7, MRAP2, ITGAX, ITGB8, CNR1, ENTPD8, SCARB1, NDRG1, TBC1D30, CSF1R, LPP, AK1, CPNE6, LIN7A, ITGA9, SLC4A10, CD274, CPNE2, PCDHGA9, PCDHGA8, NCS1, PCDHGA7, PCDHGA6, CXCR3, PCDHGA5, PCDHGA4, PCDHGA3, DDR2, PCDHGA2, LGR4, PCDHGA1, KCNQ5, PCDHGB1, CTTN, KCNQ3, PAK3, MGLL, SV2B, KCNQ2, SV2C, BOC, LTA, AKT3, EFR3B, ATP8B4, IRAK2, CTSZ, CAMK1G, PIGY, PCDHGB7, PCDHGB6, WLS, NRXN1, PCDHGB3, LDLRAD3, PCDHGB2, UBE2B, PRKCD, NCR1, PCDHGB4, CD86, BVES, CD80, PSD, PRKCZ, TNF, PPP1R12B, CALD1, PCDHGC5,		

			PCDHGC4, PCDHGC3, GPRC5B, TNFRSF4, GPRC5A, TNFRSF1B, RASGRP3, RASGRP4, PSTPIP1, TNFRSF18, TGM2, TNFRSF19, GP1BA, PIK3R5, CAMK2B, PIK3R6, MYOF, CRIM1, SLC39A1, IL2RB, LTK, CR2, IL2RA, MAP1B, EDA2R, RGS16, CYSTM1, KCNN4, RGS20, SULF2, KCNN3, SULF1, SLC5A9, ATP8A1		
GOTERM_BP_DIRECT	GO:0006955~immune response	8.76E-14	TLR1, IL13, TNFSF14, TNFSF13, TNFSF12, HLA-DMB, TLR6, HLA-DMA, IL10, TAPBP, S1PR4, IL4R, FAS, NFIL3, IL1A, LTA, LY75-CD302, CIITA, NCF4, HLA-A, HLA-C, TNFRSF14, HLA-B, ACKR4, GEM, CD40, HLA-E, HLA-DQA2, HLA-DQA1, HLA-F, TNFRSF10A, CCR9, SERPINB9, TNFRSF9, CD86, CCR7, TNFRSF10C, TNFRSF10B, CCR4, TNFRSF10D, HLA-DPA1, TNFSF12-TNFSF13, MAP3K14, GBP2, HLA-DRA, HLA-DQB1, IL1R2, HLA-DQB2, CSF2, CCL3, CCL2, TNF, HLA-DRB1, C3, ENPP2, TNFRSF8, CD70, CCL5, TNFRSF4, CD74, LIF,	2.27	4.41E-10

			CCL22, TNFRSF1B, OTUD7B, TNFRSF18, HLA-DRB5, CD22, CD4, HLA-DPB1, HLA-DOA, LY75, IL18R1, TLR10, CRIP1, IL6, IL2RA, TNFSF4, CR2, IL1RL1, CTLA4, CD276, SMAD3, SAMHD1, TNFSF9, TRIM22, AIM2, TNFSF8, RGS1, CD274, TGFBR3, ADAMDEC1, OPRD1		
KEGG_PATHWAY	hsa05166:HTLV-I infection	1.81E-11	TSPO, ADCY1, PDGFB, PDGFA, STAT5A, ADCY6, NFKB2, HLA-DMB, HLA-DMA, CTNNB1, WNT2, FOS, CDKN2A, IL15RA, FOSL1, AKT3, LTA, ZFP36, EGR1, ICAM1, WNT10B, EGR2, RELB, HLA-A, HLA-C, HLA-B, CD40, HLA-E, HLA-DQA2, HLA-DQA1, HLA-F, CCND1, CCND2, JUN, HLA-DPA1, WNT9A, MAP3K14, HLA-DRA, HLA-DQB1, WNT5A, IL1R2, CSF2, TNF, HLA-DRB1, MRAS, NFKBIA, VCAM1, HLA-DRB5, PIK3R5, HLA-DPB1, HLA-DOA, NFATC2, PIK3R2, IL6, IL2RB, IL2RA, SMAD3, FZD3, FZD5, FZD4, CDKN1A, ATF3, BAX, JAK1, JAK3	2.39	8.14E-10
GOTERM_CC_DIRECT	GO:0009986~cell surface	5.89E-12	PDGFB, PDGFA, STRC, SLC9A3, CSPG4, APP, CD44, ANK3, ROBO1, AQP11, RARA, FAS, ADAM8, ISLR2, CIITA, PTPRJ, SCUBE1, PLXNB2, HLA-A, GRIN2A, HLA-C, CD40, HLA-B, HLA-E, HLA-F, ADAMTS7, CCR9, CCR7, TNS1, THBD, TFPI, HLA-DPA1, PTGFRN, INTU, CD226, EMP2, HLA-DRA, WNT5A, PAM, ACHE, HLA-DRB1, TNFRSF12A, CLU, CD74, ITGAX, ITGB8, SCARB1, HLA-DPB1, CSF1R, PLAT, BMP2, LGALS1, MET, HILPDA, TPBG, FURIN, SLAMF1, EPHA2, NOTCH2,	2.02	1.97E-09

			CD55, SFRP1, NTRK1, CD58, TGFBR3, ARSB, SDC4, HLA-DMA, SDC2, CLEC17A, KCNQ3, SLC1A3, CEACAM1, VASN, ICAM1, CFTR, MMP16, ACKR3, NFAM1, IL6R, NRXN1, TNFRSF10A, SDC1, CD86, TNFRSF10B, CD80, TF, TNF, GPRC5B, TNFRSF4, VCAM1, ANXA9, TSPAN33, TNR, CLEC2D, GP1BA, TNFSF4, NLGN2, FZD3, FZD5, FZD4, ANXA2, SULF2, SULF1		

Table S11. Functional enrichment analysis of the downregulated DEGs in the Jurkat cell line.

**Category**	**Term**	**PValue**	**Genes**	**Fold Enrichment**	**Benjamini**
GOTERM_CC_DIRECT	GO:0009986~cell surface	9.60E-16	PDGFB, PDGFA, MMP7, APP, CD44, ROBO1, AQP11, RARA, ROBO2, FAS, ADAM8, ADAM9, ISLR2, CIITA, PTPRJ, C5AR1, PLXNB2, GRIN2A, HLA-B, CD40, HLA-F, CCR7, TNS1, THBD, TFPI, HLA-DPA1, PTGFRN, CD226, EMP2, HLA-DRA, WNT5A, PAM, ACHE, FGFR3, WNT5B, HLA-DRB1, CLU, DSCAML1, CD74, ITGAX, ITGB8, DMD, SCARB1, HLA-DPB1, CSF1R, PLAT, BMP2, LGALS1, MET, GRIN1, ABCB1, TPBG, FURIN, SLAMF1, EPHA2, NOTCH2, CD55, NLGN4Y, SFRP1, NTRK1, CD58, SFRP4, TGFBR3, SCARA5, SLC46A1, ARSB, CORIN, TRPV2, SDC4, HLA-DMA, SDC2, KISS1R, SLC1A2, SLC1A3, ICAM1, CFTR, MMP16, ACKR3, NFAM1, IL6R, NRXN1, AMBP, TNFRSF10A, CD86,	2.30	0.000

			TNFRSF10B, CD80, OTOA, TNF, ADAMTS13, CD109, GPRC5B, TNFRSF4, VCAM1, ANXA9, TSPAN33, CLEC2D, TNFSF4, NLGN2, MXRA8, ANXA4, FZD4, ANXA2, SULF2, SULF1		
GOTERM_CC_DIRECT	GO:0005886~plasma membrane	5.36E-16	SGMS2, MYLIP, CTNNB1, APP, DIRAS2, AQP11, SERPINE1, FAS, OGT, ADAM8, GNG4, PTPRJ, C4B, GRIN2A, PTPRR, LIFR, SPIRE1, CDHR5, HLA-DQA2, MARK1, HLA-DQA1, THY1, F5, RYR1, TFPI, ROR2, HLA-DPA1, CD226, HLA-DRA, NCR3LG1, IL1R2, HLA-DRB1, CYP51A1, HMGCS1, NFKBIA, CDC42EP1, DUSP15, UPK1A, OR56B1, HLA-DRB5, HLA-DPB1, CDC42EP4, HLA-DOA, CDC42EP5, PCDH12, PCDH15, PCDH18, RGS1, CD207, ERVK13-1, PKP2, RGS3, SYTL2, MARCKS, SYTL3, CDC42BPB, LIMA1, MSR1, TRPV2, TRPV3, DPP10, ATP10A, PDIA6, ST6GALNAC6, KISS1R, SNAP47, DPP6, IL13RA1, KCNG1, FMNL1, AR, ZP2, LYN, MMP16, CFTR, IL6R, RFTN1, ELMO1, GNAL, NAALAD2, CLIC6, DOK7, SCN11A, TNFSF12-TNFSF13, THEM4, RASD1, KCNH4, ENOX1, HLA-DQB1, HLA-DQB2, REPS1, FPR2, GNG12, UNC79, ACSL1, DAPP1, SOS1, VMP1, PHLDA3, SNAP25, CTLA4, DGKI, STAB2, CAPN2, TMPRSS6, RASSF3, LSP1, PTP4A3, PRSS27, PERP, SLC8A3, KCNC4, KCNC3, TNFSF14, TNFSF12, SLC2A6, ANK1, UNC5B, ANK2, SLC2A3, GALR2, RALB, IL15RA, UNC5C, EBI3, IRS2, HLA-B, HLA-F, THBD, GPR55, VAMP8, RELN, PTGFRN, DCUN1D3, XRN1, PAM, ACHE, LITAF, DRD2, MRAS, DRD4, LRIG3, ABCA3, SLCO2A1, IL12RB2, COL26A1, AMER2, ADRA2B, ADAM28, B4GALT1, TLR10, CACNA2D1, MSMO1, MYO1C, ADAM23,	1.38	0.000

			SYT11, SLC6A12, GRIN1, MET, SYT12, SLC6A15, ABCB1, CACNA2D3, SLCO2B1, SHANK2, FURIN, MYO10, GPR37, FREM2, PRKAR1B, CXCL16, CACNA1F, TRIP6, MERTK, CACNA1C, SYT17, CACNA1D, CACNA1B, IGDCC4, CLDN9, CORIN, PREX1, UCHL1, GABBR1, SDC4, SKAP2, SDC2, SLC1A2, SLC1A3, FRMD6, CSF2RB, RHOB, RAET1L, TAS2R20, ANO7, NRG2, AHNAK, EVA1A, CNTN5, ESR1, PCDH7, SIGLEC14, STXBP6, STXBP5, SLC41A2, JAM2, PHLPP1, VIM, CD109, C2CD4D, CDH8, VCAM1, CD9, CHD9, STK32A, TSPAN33, CD4, NEDD4L, NFATC2, SCNN1A, TCIRG1, SLC2A9, MAG, TNFSF4, KL, TNFSF9, TAB2, ANXA4, ANXA2, HSP90B1, CYBB, DSC3, DSC2, SCN4B, OPRD1, RAB3GAP2, SYT1, ADCY1, ATP1B1, CADM1, IL6ST, ADCY6, SYT8, LPAR3, PRKG1, S1PR1, CD44, S1PR4, CHRNA6, ISLR2, PLD2, C5AR1, MDGA1, EFNB2, PIM1, GPR132, CD40, LILRB1, CCR7, HAS1, CCR4, CD33, EFNA5, ADD2, ENPP2, GSDMB, AKAP12, IFI30, CD70, AFAP1L2, CD74, DMD, STX11, GAD1, CNKSR2, TRPC1, IKZF3, LGALS3, KLF9, IL1RL1, SMAD7, OSBPL6, PTPN13, SMAD3, MCAM, EPHA2, ABCG2, NOTCH2, EPHA7, CD55, SEMA6D, SFRP1, NTRK1, CD58, PON2, SLC46A1, SLC15A1, BCAR1, SUSD2, TAS1R3, CD151, FCRL4, FCRL3, TNFRSF11A, SLC24A3, CALN1, FAM129A, KCNMA1, ICAM1, GPR176, ICAM5, ACKR3, NFAM1, ACKR4, AMBP, TNFRSF10A, TNFRSF10C, TNFRSF10B, TNFRSF10D, PSEN2, SEMA4B, RIN1, SLC27A6, SGCE, SUSD3, ADAM12, PRF1, DCHS2, SLC39A14, GPR183, SHROOM2, FNDC4, FAT3, PLIN4, IL10RA, CLEC2D, CD22, UGT8, FAM127A, TES, IL18R1,		

			BAIAP2L1, NPR1, MAPK10, FZD4, STAT3, CISH, TJP1, ZACN, TENM2, ABCC4, PTCH1, TLR1, ILDR1, TLR6, WNT2, ROBO1, CSF3R, MCOLN2, NEGR1, PCDHGA12, PCDHGA10, PCDHGA11, MYO6, TNFRSF14, BASP1, PLCE1, LSAMP, EMP2, WNT5A, CAV2, SNX9, CAV1, WNT5B, SNAP91, FGFR3, DSCAML1, PAQR7, ITGAX, ITGB8, CNR1, SCARB1, CSF1R, LPP, AK1, CPNE6, LIN7A, ITGA9, NEDD4, CD274, CPNE2, OPCML, PCDHGA9, PCDHGA8, NCS1, PCDHGA7, PCDHGA6, CXCR3, PCDHGA5, PCDHGA4, LSR, DDR2, PCDHGA3, PCDHGA2, PCDHGA1, KCNQ5, PCDHGB1, TMEM173, PAK3, CXCR6, MGLL, SV2B, KCNQ2, BOC, LTA, ATP8B4, IRAK2, CTSZ, CAMK1G, NOXO1, PIGY, NRXN2, RIMBP2, PCDHGB7, PCDHGB6, WLS, NRXN1, PALLD, LDLRAD3, PCDHGB3, PRKCD, NCR1, PCDHGB2, PCDHGB4, CD86, BVES, XPC, CD80, MDM2, PRKCZ, TNF, PCDHGC5, PCDHGC4, PCDHGC3, GPRC5B, TNFRSF4, MTM1, RASGRP3, SYN2, PSTPIP1, TNFRSF18, TGM2, CAMK2D, TNFRSF19, CAMK2B, PIK3R6, MYOF, CRIM1, SLC39A1, IL2RB, CR2, IL2RA, MAP1B, NFASC, EDA2R, RGS16, KCNN4, RGS20, SULF2, KCNN3, SULF1, SLC5A9, MGST2, ATP8A1		
GOTERM_BP_DIRECT	GO:0006955~immune response	8.17E-15	SUSD2, TLR1, TNFSF14, TNFSF13, TNFSF12, HLA-DMB, TLR6, HLA-DMA, IL10, TAPBP, CXCL10, S1PR4, IL4R, IFNG, FAS, IL1A, LTA, LY75-CD302, CIITA, C5AR1, NCF4, TNFRSF14, HLA-B, ACKR4, GEM, CTSS, CD40, HLA-DQA2, HLA-DQA1, HLA-F, OSM, TNFRSF10A, SERPINB9, TNFRSF9, CD86, CCR7, TNFRSF10C, TNFRSF10B, CCR4,	2.42	0.000

			TNFRSF10D, HLA-DPA1, TNFSF12-TNFSF13, GBP2, HLA-DRA, HLA-DQB1, IL1R2, HLA-DQB2, GPR183, CCL3, TNF, HLA-DRB1, ENPP2, TNFRSF8, CD70, CCL5, TNFRSF4, CD74, LIF, OTUD7B, TNFRSF18, HLA-DRB5, CD22, CD4, HLA-DPB1, HLA-DOA, SBSPON, LY75, IL18R1, TLR10, CRIP1, IL6, IL2RA, TNFSF4, CR2, IL1RL1, CTLA4, CD276, SMAD3, TNFSF9, TRIM22, AIM2, TNFSF8, TNFSF10, RGS1, CD274, TGFBR3, OPRD1, BMP6		
KEGG_PATHWAY	hsa05332:Graft-versus-host disease	8.09E-13	HLA-DQB1, PRF1, IL6, TNF, HLA-DRB1, HLA-B, HLA-DMB, HLA-DMA, HLA-DQA2, HLA-DQA1, HLA-F, CD86, CD80, IFNG, HLA-DRB5, HLA-DPA1, FAS, HLA-DPB1, HLA-DOA, IL1A, HLA-DRA	6.50	0.000
GOTERM_BP_DIRECT	GO:0045944~positive regulation of transcription from RNA polymerase II promoter	6.68E-13	MEF2C, ARNT2, FSTL3, FOXO1, PDX1, RORA, IL10, IL11, CXCL10, CTNNB1, BATF, WNT2, GATA2, NLRC5, APP, CDKN2A, S1PR1, GATA6, GATA4, IFNG, GALR2, SERPINE1, RARA, OGT, IL1A, CIITA, RARG, MYO6, RXRA, RELB, SIX3, MLXIPL, ZNF649, CD40, AHR, JUNB, LILRB1, ASCL1, JUN, TFAP2A, RIPK2, CAMK1, WNT5A, DRD2, TFEB, NFKBIA, ELK3, SOX9, MEIS1, SOX8, LIF, PLAGL1, VDR, MEIS3, MEIS2, HOXA10, MAML3, ARNTL2, TCF4, IKZF4, IKZF3, BMP2, EPAS1, SMAD7, ESRRB, GRIN1, ASXL1, MAML2, MET, NR4A2, SMAD3, NR4A1, TEAD2, NEUROG3, FOXP3, NKX6-1, SHOX2, ATF3, EBF4, TRPS1, EBF2, EBF1, HOXB9, RFX3, RBPJ, BMP7, KLF4, BMP6, NAMPT, ZNF292, ELF3, FOXA3, PPARG, TP63, PAX5, ZEB2, NFKB2, CXCR3, PAX2, GLI2, ZBTB38, TMEM173, BARX2, POU5F1, PAX8, HSF4, FGF1, TNIP2, ALX4, FOSL1, FGF2, TNIP1, NR2F1, EGR1, AR, EGR2, CTBP2, FOXJ1, ESR1, NCOA7, CDK7, NLRP3, NRIP1, OSM,	1.80	0.000

			NCK2, HIPK2, FOXC1, TBX19, TNF, NR3C1, STAT4, REL, NFAT5, ETV1, BCL3, NFATC2, ETV4, PIK3R2, MAF, IL6, AHI1, PARK2, STAT1, ISL1, TMPRSS6, STAT3, TP73, TNFSF8, IRF5, RPS6KA1, IRF4, NFIB		

Table S12. Downregulated genes enriched in the apoptosis pathway for the MT-2 and Jurkat cell lines.

Sample	Genes
**MT-2 (446 genes)**	AATK,ABCG2,ACHE,ACSL4,ADAM8,ADCY6,ADRB2,AFAP1L2,AGRN,AHR,AIFM2,AIM2,AKAP12,AKT3,ALCAM,ALDH3A1,ALKBH5,ALX4,ANGPTL4,ANPEP,ANXA2,ANXA5,APOBEC3B,APP,AQP11,AQP7,AR,ARC,ASB2,ATF3,ATXN1,B4GALT5,BASP1,BAX,BBC3,BCAR1,BCL2,BCL2A1,BCL3,BCL6,BDNF,BIN1,BIRC3,BMP2,BMP7,BTG1,BTG2,C1QTNF1,C3,C5,CABLES1,CACNA1C,CADM1,CAPN2,CARD14,CASP4,CAV1,CCDC6,CCL2,CCL3,CCL5,CCND1,CCND2,CCR7,CD151,CD22,CD274,CD4,CD40,CD44,CD55,CD70,CD74,CD80,CD9,CDH1,CDKN1A,CDKN2A,CEACAM1,CEACAM5,CEACAM6,CFLAR,CFTR,CIITA,CLU,CNR1,COL1A1,COL2A1,COL4A1,COL4A2,CR2,CRADD,CSF1R,CSF2,CSF2RB,CSF3R,CSPG4,CSRNP1,CTBP2,CTLA4,CTNNB1,CTSL,CTTN,CXCR3,CYP1B1,DAPK2,DDIT3,DDIT4,DRAM1,DRD2,DUSP1,DUSP10,DUSP2,DUSP4,EBF1,EDA2R,EDIL3,EEF1A2,EFNB2,EGR1,EGR2,EGR3,EPAS1,EPHA2,ESR1,ETV5,FANK1,FAS,FBN1,FDXR,FGF1,FGF12,FGL2,FOS,FOSL1,FOXO1,FOXO6,FSTL3,FURIN,GAB1,GADD45A,GADD45B,GADD45G,GAS5,GAS6,GATA4,GATA6,GEM,GLI2,GLIS2,GLIS3,GPR132,GPR37,GPX1,GRIA3,GRIK2,HAS1,HERPUD1,HEY1,HIP1,HIPK2,HIST1H1C,HLA-B,HLA-DMA,HLA-DQB1,HPCA,HSPA1A/HSPA1B,HSPB6,HSPG2,HTATIP2,ICAM1,ID2,IER3,IGFBP3,IKZF3,IL10,IL13,IL15RA,IL1A,IL1RL1,IL23R,IL2RA,IL4R,IL6,IL6R,IL6ST,IL9R,IRAK3,IRF4,IRF5,IRS2,ITPK1,JAK3,JMY,JUN,JUNB,KAZN,KDM3A,KL,KLF10,KLF4,KLF5,KLF6,KLF7,LAMA3,LGALS1,LGALS3,LGR4,LHX2,LIF,LILRB1,LMNA,LOC102724428/SIK1,LRP1,LRRK2,LTK,LYL1,LYN,MAGEH1,MAP1B,MAP3K14,MAP3K8,MAPK10,MAPK13,MAPKAPK2,MCAM,MCL1,MEF2C,MET,mir-146,mir-24,MITF,MMP11,MOB3B,MSH2,MSR1,MT2A,MYRF,NACC1,NAMPT,NDRG1,NEK6,NFAT5,NFATC2,NFE2L2,NFKB2,NFKBIA,NFKBID,NFKBIZ,NINJ1,NLRC4,NME3,NOL3,NOTCH2,NOV,NPTX1,NQO1,NR2F1,NR3C1,NR4A1,NR4A2,NRG2,NTF4,NTN1,NTN4,NTRK1,NTRK2,NTRK3,NUAK2,OG

	T,OPTN,OSMR,PAK3,PAX5,PCDHGA1,PCDHGA10,PCDHGA11,PCDHGA12,PCDHGA2,PCDHGA3,PCDHGA4,PCDHGA5,PCDHGA6,PCDHGA7,PCDHGA8,PCDHGA9,PCDHGB1,PCDHGB2,PCDHGB3,PCDHGB4,PCDHGB6,PCDHGB7,PCDHGC3,PCDHGC4,PCDHGC5,PDCD4,PDE4D,PDGFB,PERP,PGF,PIM1,PIM2,PIM3,PKP2,PLAGL1,PLAT,PLD1,PLD2,PLK2,PLK3,PMAIP1,PON2,POU5F1,PPARA,PPARG,PPP1R15A,PPP2R2B,PRF1,PRKAA2,PRKCD,PRKCZ,PSEN2,PTCH1,PTGS2,PTHLH,PTPN13,RALB,RARA,RARG,RASSF3,RBPJ,RCAN2,REL,REM2,RGS3,RGS6,RHOB,RIPK2,ROR2,RPS6KA1,RRAD,RRM2B,RUNX1T1,RUNX3,RXRA,RYBP,RYR2,S100A1,S100A11,S100A4,S100A6,S1PR1,S1PR4,SCARB1,SEMA3B,SERPINB9,SERPINE1,SFRP1,SGMS2,SGPP2,SIGLEC5,SKIL,SLAMF1,SLC1A3,SMAD3,SMAD7,SMN1/SMN2,SNX7,SOCS1,SOCS3,SOX5,SPECC1,SQSTM1,SRCIN1,SRI,ST3GAL1,ST8SIA1,STAT1,STAT3,STAT4,STAT5A,STC1,STEAP3,SULF1,TAB2,TAGLN2,TCF4,TEC,TF,TFAP2A,TFEB,TFPI,TGFBR3,TGM2,THBD,THY1,TIMP1,TLR1,TLR6,TNC,TNF,TNFAIP2,TNFAIP3,TNFRSF10A,TNFRSF10B,TNFRSF10C,TNFRSF10D,TNFRSF11A,TNFRSF12A,TNFRSF14,TNFRSF18,TNFRSF19,TNFRSF1B,TNFRSF4,TNFRSF8,TNFRSF9,TNFSF12,TNFSF13,TNFSF14,TNIP1,TNIP2,TP63,TP73,TRADD,TRAF1,TRIB1,TRIM29,TRPS1,TSC22D3,TSPO,TYMP,UCHL1,UNC5B,USP18,USP2,VASP,VCAN,VDR,VIM,WISP1,WNT5A,XCL1,ZEB2,ZFAND5,ZFP36,ZHX2
**Jurkat (409 genes)**	ABCB1,ABCG2,ACHE,ACSL4,ADAM12,ADAM8,ADAMTS13,ADCY6,AFAP1L2,AGA,AHR,AIFM2,AIM2,AKAP12,ALCAM,ALX4,ANXA2,ANXA5,APOBEC3B,APP,AQP11,AR,ASB2,ASS1,ATF3,ATXN1,B4GALT5,BASP1,BAX,BBC3,BCAR1,BCL2,BCL2A1,BCL3,BCL6,BDNF,BHLHE41,BIN1,BIRC3,BMP2,BMP6,BMP7,BTG2,C1QTNF1,C5AR1,CACNA1C,CADM1,CAMK2D,CAPN2,CASP4,CAV1,CCDC6,CCL3,CCL5,CCND1,CCND2,CCR7,CD151,CD22,CD274,CD33,CD4,CD40,CD44,CD55,CD70,CD74,CD80,CD9,CDK3,CDKN1A,CDKN2A,CERS1,CFLAR,CFTR,CIITA,CLU,CNR1,COL1A1,COL2A1,COL4A1,COL4A2,CR2,CRADD,CSF1R,CSF2RB,CSF3R,CTBP2,CTLA4,CTNNB1,CTSL,CTSS,CXCL10,CXCR3,CYBB,CYP19A1,CYP1B1,DEPDC1,DHCR24,DMGDH,DRAM1,DRD2,DUSP1,DUSP2,DUSP4,EBF1,EBF4,EDA2R,EDIL3,EEF1A2,EFNB2,EGR1,EGR2,ELMO1,EPAS1,EPHA2,ESR1,FANK1,FAS,FBN1,FDXR,FGF1,FGF12,FGF2,FGF5,FGFR3,FGL2,FN1,FOSL1,FOXO1,FSTL3,FURIN,GABBR1,GADD45A,GADD45B,GADD45G,GALR2,GAS5,GATA4,GATA6,GEM,GLI2,GLS2,GNAL,GPR132,GPR37,GPX1,GRIN1,HAS1,HIPK2,HIST1H1C,HLA-B,HLA-DMA,HLA-DQB1,HOXA9,HSPA1A/HSPA1B,HTATIP2,ICAM1,ID2,IER3,IFNG,IGFBP3,IKZF3,IL10,IL10RA,IL11,IL15RA,IL1A,IL1RL1,IL2RA,IL4R,IL6,IL6R,IL6ST,IL9R,IRAK3,IRF4,IRF5,IRS2,ITPK1,JAK3,JMY,JUN,JUNB,KAZN,KL,KLF4,LAMA3,LGALS1,LGALS3,LIF,LILRB1,LMNA,LYL1,LYN,MAGEH1,

	MAP1B,MAP3K8,MAPK10,MAPK13,MAPK8,MAPKAPK2,MCAM,MDH1,MDM2,MEF2C,MERTK,MET,mir-146,mir-24,mir-29,MMP11,MMP7,MOB3B,MSR1,MTM1,NAMPT,NEK6,NFAT5,NFATC2,NFIB,NFKB2,NFKBIA,NFKBIZ,NLRP3,NME3,NOTCH2,NOV,NPM1,NPR1,NPTX1,NR2F1,NR3C1,NR4A1,NR4A2,NRG2,NTF4,NTRK1,NTRK2,NTRK3,NUAK2,OGT,OPTN,OSM,PAK3,PALLD,PAX2,PAX5,PCDHGA1,PCDHGA10,PCDHGA11,PCDHGA12,PCDHGA2,PCDHGA3,PCDHGA4,PCDHGA5,PCDHGA6,PCDHGA7,PCDHGA8,PCDHGA9,PCDHGB1,PCDHGB2,PCDHGB3,PCDHGB4,PCDHGB6,PCDHGB7,PCDHGC3,PCDHGC4,PCDHGC5,PDCD4,PDE4D,PDGFB,PDX1,PERP,PGF,PIM1,PIM2,PKP2,PLA2G7,PLAGL1,PLAT,PLD1,PLD2,PLK2,PLK3,PMAIP1,PON2,POU5F1,PPARG,PPP1R15A,PPP2R2B,PRF1,PRKCD,PRKCZ,PRKN,PSEN2,PTCH1,PTGS2,PTHLH,PTPN13,RALB,RARA,RARG,RASSF3,RBPJ,RCAN2,REL,RGS3,RHOB,RIPK2,ROR2,RPS6KA1,RRM2B,RTKN,RXRA,S100A11,S100A4,S100A6,S1PR1,S1PR4,SCARB1,SEMA3B,SERPINB9,SERPINE1,SFRP1,SFRP4,SGMS2,SGPP2,SIGLEC5,SKIL,SLAMF1,SLC1A3,SLC46A1,SMAD3,SMAD7,SMN1/SMN2,SNX7,SOCS1,SOCS3,SORCS2,SOX5,SRCIN1,SRI,SRXN1,ST8SIA1,STAT1,STAT3,STAT4,STAT5A,STC1,STEAP3,SULF1,TAB2,TAGLN2,TBX3,TCF4,TEC,TFAP2A,TFEB,TFPI,TGFBR3,TGM2,THBD,THG1L,THY1,TIMP1,TLR1,TLR6,TNC,TNF,TNFAIP2,TNFAIP3,TNFRSF10A,TNFRSF10B,TNFRSF10C,TNFRSF10D,TNFRSF11A,TNFRSF14,TNFRSF18,TNFRSF19,TNFRSF4,TNFRSF8,TNFRSF9,TNFSF10,TNFSF12,TNFSF13,TNFSF14,TNIP1,TNIP2,TP63,TP73,TRIB1,TRPS1,TRPV2,TSC22D3,TYMP,UBD,UCHL1,UGT8,UNC5B,UNC5C,USP2,VCAN,VDR,VIM,WNT5A,XPC,ZEB2,ZFAND5
**Overlap of MT-2 and Jurkat (345 genes)**	ABCG2,ACHE,ACSL4,ADAM8,ADCY6,AFAP1L2,AHR,AIFM2,AIM2,AKAP12,ALCAM,ALX4,ANXA2,ANXA5,APOBEC3B,APP,AQP11,AR,ASB2,ATF3,ATXN1,B4GALT5,BASP1,BAX,BBC3,BCAR1,BCL2,BCL2A1,BCL3,BCL6,BDNF,BIN1,BIRC3,BMP2,BMP7,BTG2,C1QTNF1,CACNA1C,CADM1,CAPN2,CASP4,CAV1,CCDC6,CCL3,CCL5,CCND1,CCND2,CCR7,CD151,CD22,CD274,CD4,CD40,CD44,CD55,CD70,CD74,CD80,CD9,CDKN1A,CDKN2A,CFLAR,CFTR,CIITA,CLU,CNR1,COL1A1,COL2A1,COL4A1,COL4A2,CR2,CRADD,CSF1R,CSF2RB,CSF3R,CTBP2,CTLA4,CTNNB1,CTSL,CXCR3,CYP1B1,DRAM1,DRD2,DUSP1,DUSP2,DUSP4,EBF1,EDA2R,EDIL3,EEF1A2,EFNB2,EGR1,EGR2,EPAS1,EPHA2,ESR1,FANK1,FAS,FBN1,FDXR,FGF1,FGF12,FGL2,FOSL1,FOXO1,FSTL3,FURIN,GADD45A,GADD45B,GADD45G,GAS5,GATA4,GATA6,GEM,GLI2,GPR132,GPR37,GPX1,HAS1,HIPK2,HIST1H1C,HLA-B,HLA-DMA,HLA-DQB1,HSPA1A/HSPA1B,HTATIP2,ICAM1,ID2,IER3,IGFBP3,IKZF3,IL10,IL15RA,IL1A,IL1RL1,IL2RA,IL4R,IL6,IL6R,IL6ST,IL9R,IRAK3,IRF4,IRF5,IRS2,ITPK1,JAK3,JMY,JUN,JUNB,KAZN,KL,KLF4,LAMA3,LGALS1,LGAL

	S3,LIF,LILRB1,LMNA,LYL1,LYN,MAGEH1,MAP1B,MAP3K8,MAPK10,MAPK13,MAPKAPK2,MCAM,MEF2C,MET,mir-146,mir-24,MMP11,MOB3B,MSR1,NAMPT,NEK6,NFAT5,NFATC2,NFKB2,NFKBIA,NFKBIZ,NME3,NOTCH2,NOV,NPTX1,NR2F1,NR3C1,NR4A1,NR4A2,NRG2,NTF4,NTRK1,NTRK2,NTRK3,NUAK2,OGT,OPTN,PAK3,PAX5,PCDHGA1,PCDHGA10,PCDHGA11,PCDHGA12,PCDHGA2,PCDHGA3,PCDHGA4,PCDHGA5,PCDHGA6,PCDHGA7,PCDHGA8,PCDHGA9,PCDHGB1,PCDHGB2,PCDHGB3,PCDHGB4,PCDHGB6,PCDHGB7,PCDHGC3,PCDHGC4,PCDHGC5,PDCD4,PDE4D,PDGFB,PERP,PGF,PIM1,PIM2,PKP2,PLAGL1,PLAT,PLD1,PLD2,PLK2,PLK3,PMAIP1,PON2,POU5F1,PPARG,PPP1R15A,PPP2R2B,PRF1,PRKCD,PRKCZ,PSEN2,PTCH1,PTGS2,PTHLH,PTPN13,RALB,RARA,RARG,RASSF3,RBPJ,RCAN2,REL,RGS3,RHOB,RIPK2,ROR2,RPS6KA1,RRM2B,RXRA,S100A11,S100A4,S100A6,S1PR1,S1PR4,SCARB1,SEMA3B,SERPINB9,SERPINE1,SFRP1,SGMS2,SGPP2,SIGLEC5,SKIL,SLAMF1,SLC1A3,SMAD3,SMAD7,SMN1/SMN2,SNX7,SOCS1,SOCS3,SOX5,SRCIN1,SRI,ST8SIA1,STAT1,STAT3,STAT4,STAT5A,STC1,STEAP3,SULF1,TAB2,TAGLN2,TCF4,TEC,TFAP2A,TFEB,TFPI,TGFBR3,TGM2,THBD,THY1,TIMP1,TLR1,TLR6,TNC,TNF,TNFAIP2,TNFAIP3,TNFRSF10A,TNFRSF10B,TNFRSF10C,TNFRSF10D,TNFRSF11A,TNFRSF14,TNFRSF18,TNFRSF19,TNFRSF4,TNFRSF8,TNFRSF9,TNFSF12,TNFSF13,TNFSF14,TNIP1,TNIP2,TP63,TP73,TRIB1,TRPS1,TSC22D3,TYMP,UCHL1,UNC5B,USP2,VCAN,VDR,VIM,WNT5A,ZEB2,ZFAND5

Table S13. Upstream regulator prediction using the DEGs of HIV+ samples of the MT-2 cell line.

**Upstream Regulator**	**Expr Log Ratio**	**Molecule Type**	**Predicted Activation State**	**Activation z-score**	**p-value of overlap**
TNF	-4.547	cytokine	Inhibited	-7.586	3.25E-53
IFNG		cytokine	Inhibited	-6.575	1.56E-37
TGFB1		growth factor	Inhibited	-3.527	1.29E-34
lipopolysaccharide		chemical drug	Inhibited	-7.909	5.25E-33

IL2		cytokine	Inhibited	-4.6	2.45E-32
dexamethasone		chemical drug		-0.259	1.44E-30
IL1B		cytokine	Inhibited	-6.003	5.23E-30
ID2	-5.779	transcription regulator		-1.467	1.48E-28
filgrastim		biologic drug		0.906	2.59E-28
ID3	2.528	transcription regulator		-1.627	4.34E-26
CD40LG	∞	cytokine	Inhibited	-5.066	4.59E-26
IL4	2.757	cytokine	Inhibited	-5.787	6.65E-26
TCR		complex	Inhibited	-4	1.97E-24
IL5		cytokine	Inhibited	-5.161	4.22E-24
Immunoglobulin		complex		-1.204	1.82E-23
NFkB (complex)		complex	Inhibited	-7.289	1.92E-23
poly rI:rC-RNA		biologic drug	Inhibited	-6.717	1.88E-22
CD3		complex	Inhibited	-6.12	2.45E-22

Table S14. Upstream regulator prediction using the DEGs of HIV+ samples of the Jurkat cell line

Upstream Regulator	Expr Log Ratio	Molecule Type	Predicted Activation State	Activation z-score	p-value of overlap
TNF	-3.621	cytokine	Inhibited	-6.606	2.52E-46
TGFB1		growth factor		-1.491	7.91E-38
IFNG	-5.153	cytokine	Inhibited	-5.703	5.89E-37
dexamethasone		chemical drug		-0.793	8E-33
lipopolysaccharide		chemical drug	Inhibited	-7.422	2.69E-30
filgrastim		biologic drug		-0.374	2.34E-27
IL2		cytokine	Inhibited	-4.725	3.63E-27
ID2	-2.892	transcription regulator		-1.29	1.1E-24
IL4		cytokine	Inhibited	-5.009	1.39E-24
ID3		transcription regulator	Inhibited	-2.094	5.43E-24
CD40LG		cytokine	Inhibited	-5.09	9.51E-24
Immunoglobulin		complex		-1.677	2.01E-23
IL1B		cytokine	Inhibited	-5.788	3.2E-23
NFkB (complex)		complex	Inhibited	-6.842	3.38E-23
CD40	-5.778	transmembrane receptor	Inhibited	-4.008	1.26E-21
IL15		cytokine	Inhibited	-3.944	4.84E-21
TCR		complex	Inhibited	-3.595	1.23E-20
IL5		cytokine	Inhibited	-4.257	2.32E-20

Table S15. DAVID functional enrichment for genes with differentially expressed exons in the Jurkat cell line.

Category	Term	Count	p-value	Benjamini
GOTERM_BP_DIRECT	antigen processing and presentation of endogenous peptide antigen via MHC class I via ER pathway, TAP-independent	3	5.60E-04	5.50E-01
GOTERM_BP_DIRECT	apoptotic process	18	2.20E-03	8.00E-01
GOTERM_BP_DIRECT	interferon-gamma-mediated signaling pathway	6	2.90E-03	7.60E-01
GOTERM_BP_DIRECT	fat cell differentiation	6	3.30E-03	7.00E-01
GOTERM_BP_DIRECT	antigen processing and presentation of exogenous peptide antigen via MHC class I, TAP-independent	3	6.40E-03	8.40E-01
GOTERM_BP_DIRECT	membrane protein proteolysis	3	7.90E-03	8.50E-01
GOTERM_BP_DIRECT	positive regulation of ATPase activity	4	8.70E-03	8.30E-01

Table S16. Upstream regulator prediction using overlapped genes between DEGs and DMGs in HIV+ samples of the MT-2 cell line

Upstream Regulator	Expr Log Ratio	Molecule Type	Predicted Activation State	Activation z-score	Flags	p-value of overlap
IFNG		cytokine	Inhibited	-2.374		5.87E-09
IL4		cytokine	Inhibited	-2.017		1.58E-08
TGFB1		growth factor		-0.744		2.11E-08

lipopolysaccharide		chemical drug	Inhibited	-4.369		2.75E-08
CD40		transmembrane receptor		-1.101		5.48E-08
tetradecanoylphorbol acetate		chemical drug		-1.429		7.26E-08
ID2		transcription regulator		-1.633		7.92E-08
Tcf7		transcription regulator		0.2		0.00000013
ID3		transcription regulator		-0.563		0.000000147
GATA3	3.783	transcription regulator		0.736		0.000000228
TLR7/8		group	Inhibited	-2.646	bias	0.000000243
NFkB (complex)		complex	Inhibited	-3.329	bias	0.000000244
TNF	-4.547	cytokine	Inhibited	-4.421		0.000000644
ionomycin		chemical reagent		-0.651	bias	0.000000945
Immunoglobulin		complex		-0.464		0.00000111
IL15		cytokine		-1.371		0.00000141
cyclosporin A		biologic drug		1.509		0.00000191
IL7		cytokine	Inhibited	-2.757	bias	0.00000239

Table S17. Upstream regulator prediction using overlapped genes between DEGs and DMGs in HIV+ samples of the Jurkat cell line

Upstream Regulator	Expr Log Ratio	Molecule Type	Predicted Activation State	Activation z-score	Flags	p-value of overlap
TGFB1		growth factor		-1.098		3.24E-08
ID3		transcription regulator		-0.213		3.96E-08
ID2		transcription regulator		-1.134		1.33E-07
Histone h3		group				1.46E-06
PRDM5		transcription regulator				1.70E-06
COMMD3-BMI1		transcription regulator		0.692	bias	1.87E-06
IFNG		cytokine		-1.36		2.41E-06
Tcf7		transcription regulator		-0.218		3.60E-06
IL5		cytokine		-1.921	bias	3.80E-06
SLC30A3		transporter		-1.982	bias	9.00E-06
tetradecanoylphorbol acetate		chemical drug		-1.807		1.12E-05
CD38		enzyme		-1.673	bias	1.19E-05
GATA3	4.162	transcription regulator		0.513		1.71E-05
HHIP	7.758	other				1.82E-05
IRS1		enzyme				2.52E-05
Nr1h		group		1.373		3.11E-05
ABCA1		transporter		0.038		3.12E-05

OSM		cytokine		-1.314		3.18E-05

Table S18. Demethylation treatment using 5-azac

	Promoter DNA methylation level (RPKM)		Gene expression level (RPKM)	
	HIV^+	HIV5aza	HIV^+	HIV5aza
LGALS3	1.18	0.07	0.96	202.98
TRAF1	2.93	0.37	4.87	95.35
NLRP3	0.42	0.13	0.07	0.72
APP	0.65	0.25	4.45	10.18
TBX3	1.37	0.10	0.00	1.64
NTRK2	1.14	0.00	0.00	14.99

Table S19. Expression levels of *CPT1A*, *TNF*, and *LEF1* in HIV infected and uninfected PBMC samples

	**Y**		**Z**	
	**HIV infected**	**Uninfected**	**HIV infected**	**Uninfected**
**CPT1A**	40.18	20.47	36.45	17.85
**LEF1**	73.15	32.18	80.94	136.95
**TNF**	17.03	73.00	21.33	37.33

Footnote: The unit of numbers in this table is “Fragments Per Kilobase of transcript per Million mapped reads”.
