# Supplementary material for: Genome-Wide Characterization of Host Transcriptional and Epigenetic Alterations During HIV Infection of T Lymphocytes
Source: Front Immunol. 2020 Sep 10;11:2131. doi: 10.3389/fimmu.2020.02131 (PMC7511662; doi:10.3389/fimmu.2020.02131)
Supplement: Supplementary file 3 [file Data_Sheet_3.pdf]

Supplemental Text

Table of Contents

Supplemental Results 2

Differentially methylated regions (DMRs) were enriched in specific genomic regions 2

Alternative splicing in HIV infected T cells 2

Detailed description and discussion of proposed candidate genes 3

Reference 7

Supplemental Results

Differentially methylated regions (DMRs) were enriched in specific genomic regions

In contrast, both hyper- and hypo-methylated DMRs had a significant depletion in long interspersed nuclear elements (LINEs) for both cell lines and the p-value for the enrichment is the lowest among all the genomic regions (Fisher's exact test; P<2.20E-16, hyper-methylated DMRs, MT-2 cell line; P=<2.20E-16, hypo-methylated DMRs, MT-2 cell line; P=8.19E-16, hyper-methylated DMRs, Jurkat cell line; P<2.20E-16, hypo-methylated DMRs, Jurkat cell line). When comparing hyper- with hypo-methylated DMR, we found in pseudo region were more enriched in hyper- than hypo-methylated DMRs (Fisher's exact test, P=9.59E-03, MT-2 cell line; P=2.26E-04, Jurkat cell line).

Alternative splicing in HIV infected T cells

Then we moved to investigate the alternative splicing of transcript during HIV infection. In Jurkat cell line, we identified 282 genes that contained differentially expressed exons on their transcripts, among which only 23 and 87 showed significant upregulation and downregulation, respectively, in gene expression, suggesting that the remaining 172 genes (172=282-23-87) were alternative spliced genes without expression changes. To further investigate the affected cellular functions, we used these 282 genes to perform functional enrichment analysis and found that "apoptotic process" was enriched (p = 2.20E-03, S16 Table). These results suggested

the alternative splicing as an additional mechanism that may contribute to the T cell apoptosis during HIV+ infection. Genes related to this biological process included FAM3B, PLAGL1, LGALS1, GADD45G and NR4A1, which not only contained differentially expressed exons, but also showed lower expression by more than 5 folds at the whole gene levels in HIV+ Jurkat cell Line. Specifically, PLAGL1, also had hyper-methylated DMRs located in its promoter region in HIV infected sample, which indicated the potential regulation between DNA methylation and transcription on this particular gene. Notably, PLAGL1 can act as a growth suppressor and apoptotic process regulator. Moreover, alternative splicing on the 5′-UTR of PLAGL1 was found in peripheral blood leucocyte and expression of this gene was closely associated with a CpG island on promoter region [28]. LGALS1, which codes for a protein that is important in cell to cell adhesion, is a strong inducer of T-cell apoptosis. In our study, LGALS1 was downregulated by 15.6 folds at the gene level following HIV-1 infection and was alternatively spliced, where only two transcripts (in red box) were upregulated and the other transcripts were downregulated after infection by HIV-1 (Additional file 1: Fig S7).

Detailed description and discussion of proposed candidate genes

Regarding the candidate genes related to apoptosis we proposed for further research (**Table 2**), for MT-2 cell line, *APP* contained hypo-methylated DMR in gene body and was downregulated by 18.5 folds. *CD38* contained hyper-DMR in gene body and was upregulated by 5.24 folds. Increased *CD38* expression in T lymphocytes is a marker of HIV dissemination into the central nervous system [1]. *SMAD3* contained hypo-

methylated DMR in gene body and was downregulated by 7.55 folds. *SMAD3* was reported to regulate *MCP-1* gene transcription with HIV-1 Tat in human glial cells [2]. Activation of *SMAD3* could promote *HIPK2*-induced cell apoptosis in kidney fibrosis [3]. Our study first reported the correlation between gene body DNA methylation and gene expression of *SMAD3* after HIV infection. *MGMT* contained hyper-methylated DMR in gene body and was upregulated (FPKM of *MGMT* was 77 and 0 respectively in HIV-infected and uninfected samples). *MGMT* and *DAPK* were both reported to be hyper-methylated in lymphomagenesis of immunodeficient hosts [4]. *HIPK2* contained hypo-methylated DMR in gene body and was downregulated by 20.1 folds. HIV-1 had been reported to induce *HIPK2* expression in kidney cells. HIV infection increased the protein concentrations of *HIPK2* by promoting oxidative stress, which inhibited the seven in absentia homolog 1 (SIAH1)-mediated proteasomal degradation of *HIPK2* [5]. *LEF1* contained hyper-methylated DMR in gene body and upregulated in terms of gene expression by 32.8 folds. For *GATA3*, we identified one DMR significantly hypo-methylated in HIV infected sample near the transcription start site and found that expression level of *GATA3* was significantly increased by 13.8 folds. *GATA3* is one of the important targets of HIV-1 replication often expressed in T lymphocytes. IL-4+ phenotype was closely related with the expression of *GATA3* in HIV-schistosome co-infected mice[6]. As for the other genes that were selected in MT-2 cell line, *LGALS3* contained hyper-methylated DMR in promoter and was surprisingly downregulated by 202 folds. Partially consistent with our data, a previous study reported downregulation of Galectin-3 was observed in HIV-infected human oral epithelial cells [7]. In addition, *LGALS3* was reported to facilitate HIV-1 budding via Alix and was also identified as one of the Th17-specifc HIV dependency factors by a meta-analysis [8, 9]. In our data, *LGALS3* was downregulated and contained a hyper-methylated DMR in HIV-1 infected

MT-2 cell, suggesting that T cell might be combating with HIV to avoid cell death. To our best knowledge, there is no previous study that has reported the observation of DNA methylation regulation on promoter region of *LGALS3* in HIV infected T cells. *CDKN2A* contained hypo-methylated DMR in gene body and was downregulated. A case-control study in South Africa reported that telomere length and *CDKN2A* expression were both consistent with increased biological ageing in HIV-infected individuals [10]. *PDX1* contained hyper-methylated DMR in promoter and was downregulated (The expression level in 42.25 in control cell and 0 in HIV^+ cell). *TRAF1* contained hyper-methylated DMR in promoter region and was downregulated by 18 folds. *TRAF1* expression negatively correlates with programmed death expression and HIV load, and knockdown of *TRAF1* in CD8 T cells from viral controllers resulted in decreased HIV suppression *ex vivo* [11]. Therefore, *TRAF1*, a potent co-stimulator of immunological responses, could be an attractive candidate for immunotherapy of AIDS. *RYR2* contained hypo-methylated DMR in gene body and was downregulated by 20.1 folds. It has been demonstrated that Snapin, a positive regulator of stimulation- induced Ca^{2+} release through RyR, is necessary for HIV-1 replication in T cells [12]. NLRP3 was hypo-methylated in gene body and downregulated in HIV+ MT-2 cell. Notably, the effect of DNA. HIV-1 had been reported to induce the expression of NLRP3 and IL-1β in healthy controls and initiate activation of the *NLRP3* inflammasome in monocyte-derived macrophages [13].

For Jurkat cell line, several well-known apoptosis-related overlapped genes between DEGs and DMGs were also selected for further analysis. They have negatively correlated relationships between gene expression and promoter DNA methylation, or

positive correlated relationships between gene expression and gene body DNA methylation. Several well-known apoptosis-related genes were shared by both MT-2 and Jurkat cell lines: *APP* (hypo-methylated DMR in gene body and was downregulated by 24.3 folds), *CD38* (hyper-methylated DMR in gene body and was upregulated by 4.2 folds), *SMAD3* (hyper-methylated DMR in promoter, hypo-methylated DMR in gene body and was downregulated by 5.5 folds), *MGMT* (hyper-methylated DMR in gene body and was upregulated by 54 folds), *HIPK2* (hypo-methylated DMR in gene body and was downregulated by 14.9 folds), *LEF1* (contain hypo-methylated DMR in promoter and was upregulated by 33.2 folds), and *GATA3* (hypo-methylated DMR in promoter and was upregulated by 17.9 folds). The methylation effect on *APP* was confirmed by demethylated agent 5azaC. *PDX1* contained hyper-methylated DMR in promoter and was downregulated by 366.7 folds. PDX1, an islet transcription factor, could be fused with protein transduction domains to facilitate the entry into cells. The methylation and expression regulations of this gene during HIV infection so far have not been reported. *RYR2* contained hypo-methylated DMR in gene body and was downregulated. In addition to the genes described above, many other possible apoptosis related genes also showed correlation between DNA methylation on promoter and gene expression. For example, TBX3, CPT1A and NTRK2 also have potential to be involved in the process of HIV infection of T cell in the context of their changes in both gene expression and DNA methylation validated in this study. These genes would be worth undergoing further investigations. These genes would be worth undergoing further investigations.

Reference

1. Dentone C, Fenoglio D, Schenone E, Cenderello G, Prinapori R, Signori A, et al. Increased CD38 expression on T lymphocytes as a marker of HIV dissemination into the central nervous system. HIV clinical trials. 2015;16(5):190-6.
2. Abraham S, Sawaya BE, Safak M, Batuman O, Khalili K, Amini S. Regulation of MCP-1 gene transcription by Smads and HIV-1 Tat in human glial cells. Virology. 2003;309(2):196-202.
3. Jin YM, Ratnam K, Chuang PY, Fan Y, Zhong YF, Dai Y, et al. A systems approach identifies HIPK2 as a key regulator of kidney fibrosis. Nat Med. 2012;18(4):580-8.
4. Rossi D, Gaidano G, Gloghini A, Deambrogi C, Franceschetti S, Berra E, et al. Frequent aberrant promoter hypermethylation of O6-methylguanine-DNA methyltransferase and death-associated protein kinase genes in immunodeficiency-related lymphomas. British journal of haematology. 2003;123(3):475-8.
5. Jin Y, Ratnam K, Chuang PY, Fan Y, Zhong Y, Dai Y, et al. A systems approach identifies HIPK2 as a key regulator of kidney fibrosis. Nature medicine. 2012;18(4):580-8.
6. Yin J, Dai A, Arango T, Kasinathan RS, Greenberg RM, Boyer JD. IL-4 and IFN-gamma induced by human immunodeficiency virus vaccine in a schistosome infection model. Human vaccines & immunotherapeutics. 2012;8(11):1555-63.
7. Yohannes E, Ghosh SK, Jiang B, McCormick TS, Weinberg A, Hill E, et al. Proteomic Signatures of Human Oral Epithelial Cells in HIV-Infected Subjects. PloS one. 2011;6(11).
8. Cleret-Buhot A, Zhang YW, Planas D, Goulet JP, Monteiro P, Gosselin A, et al. Identification of novel HIV-1 dependency factors in primary CCR4(+)CCR6(+)Th17 cells via a genome-wide transcriptional approach. Retrovirology. 2015;12.
9. Wang SF, Tsao CH, Lin YT, Hsu DK, Chiang ML, Lo CH, et al. Galectin-3 promotes HIV-1 budding via association with Alix and Gag p6. Glycobiology. 2014;24(11):1022-35.
10. Pathai S, Lawn SD, Gilbert CE, McGuinness D, McGlynn L, Weiss HA, et al. Accelerated biological ageing in HIV-infected individuals in South Africa: a case-control study. Aids. 2013;27(15):2375-84.
11. Wang C, McPherson AJ, Jones RB, Kawamura KS, Lin GH, Lang PA, et al. Loss of the signaling adaptor TRAF1 causes CD8+ T cell dysregulation during human and murine chronic infection. The Journal of experimental medicine. 2012;209(1):77-91.
12. Kinoshita SM, Kogure A, Taguchi S, Nolan GP. Snapin, positive regulator of stimulation- induced Ca(2)(+) release through RyR, is necessary for HIV-1 replication in T cells. PloS one. 2013;8(10):e75297.
13. Hernandez JC, Latz E, Urcuqui-Inchima S. HIV-1 induces the first signal to activate the NLRP3 inflammasome in monocyte-derived macrophages. Intervirology. 2014;57(1):36-42.
